# Supplementary material for: Development and Evaluation of a Five-Component Toolkit for Internal Medicine Residents Applying for Subspecialty Fellowships
Source: MedEdPORTAL. 2022 Mar 14;18:11228. doi: 10.15766/mep_2374-8265.11228 (PMC8918571; doi:10.15766/mep_2374-8265.11228)
Supplement: Supplementary file 1 — Elements of the Fellowship Application Toolkit.docxFellowship Application Guide.docxFellowship Application Information Night.pptxSubspecialty Breakout Room Questions.docxPreparing for Virtual Interviews.pptxMock Virtual Interview.docxSurvey Instrument.docx [file mep_2374-8265.11228-s001.zip › A. Elements of the Fellowship Application Toolkit.docx]

**Appendix A: Elements of the Fellowship Application Toolkit**

| **Fellowship application advising resource** | **Year introduced** | **Explanation of resource, materials, and/or training needed** | **Approximate time to create/plan** | **Approx cost^a^** |
| --- | --- | --- | --- | --- |
| 1. Fellowship Application Guidebook | 2019 | - Created a Fellowship Application Guidebook with detailed logistics of the fellowship application process including sections on resources, timeline, costs, short tracking, getting information on programs, letters of recommendation, personal statements, ERAS nuts and bolts, interviews, and post-interview communication. - Solicited input from fellowship program directors (PDs) during the development of the content and we update it annually based on resident feedback. | - 30 hours to create the first year - 5 hours to update in subsequent years | $0 |
| 2. Fellowship Application Information Night | 2019 | - Hosted a two-hour in-person Fellowship Application Information Night. The first hour was a general overview of the fellowship application process and the second hour was a subspecialty-specific breakout room with the fellowship PD, associate PD, and/or fellows. - Residents were asked to submit questions in advance and all rooms had an identical list of anonymous questions for the fellowship PDs to address (Supplementary Table 1). | - 20 hours to plan the first year - 5-10 hours to organize in subsequent years | $400 if dinner provided |
| 3. Alumni Contact List | 2019 | - Created an Alumni Contact List from the prior ten years that included residency graduation year, fellowship specialty and location, current position, and clinical interests. - A residency administrator helped update and maintain this list annually. The list was distributed as a Excel file by email and stored on Box. | - 5-10 hours to create the first year - 5 hours to update annually | $0 |
| 4. Personal Statement Resources and Coaches | 2020 | - Developed Personal Statement Resources which comprised a personal statement template (structured guidance by paragraph, see Supplementary Table 2) and a repository of example personal statements representing different specialties and career goals. - Paired all interested residents with a Faculty Personal Statement Coach (in a different subspecialty to that in which they were applying, to preserve confidentiality) to review and give input on their personal statement. | - 20 hours to plan the first year | $0 |
| 5. Virtual Interview Workshop and Mock Virtual Interviews | 2020 | - Hosted a one-hour Interview Information Night that covered interview preparation, tips for virtual interviewing, and post-interview communication. - Paired interested residents with a faculty member (in a different subspecialty to that in which they were applying) for mock interviews. These were structured as 30-minute interviews with 20 minutes for mock interview questions (at least one behavioral question) and 10 minutes for feedback. - All faculty were provided sample questions and an evaluation rubric (Supplementary Table 3). | - 15-20 hours to plan the first year | $0 |

^a^ Costs do not include time/efforts spent planning/executing the resource/event
