# Supplementary material for: Development and Evaluation of a Five-Component Toolkit for Internal Medicine Residents Applying for Subspecialty Fellowships
Source: MedEdPORTAL. 2022 Mar 14;18:11228. doi: 10.15766/mep_2374-8265.11228 (PMC8918571; doi:10.15766/mep_2374-8265.11228)
Supplement: Supplementary file 1 — Elements of the Fellowship Application Toolkit.docxFellowship Application Guide.docxFellowship Application Information Night.pptxSubspecialty Breakout Room Questions.docxPreparing for Virtual Interviews.pptxMock Virtual Interview.docxSurvey Instrument.docx [file mep_2374-8265.11228-s001.zip › D. Subspecialty Breakout Room Questions.docx]

**Appendix D: Subspecialty breakout room sample questions**

| **Subspecialty breakout room sample questions** |
| --- |
| - - **Letters of recommendation:**     - Is it preferable to have a letter from a well-known senior faculty member who doesn’t know me well or from a junior faculty member who knows me well?     - What about a letter from a hospitalist or someone outside of the subspecialty?   - **Research/Academic areas of interest:**     - What do programs look for in terms of research (involvement vs abstracts vs papers)? How important is it to have a poster vs. a manuscript? How do you view being a first author vs. middle author?     - What advice do you have for someone interested in this subspecialty who has minimal research experience?     - Should I take a year off if I haven’t done “enough” research? What weaknesses or gaps, if any, make you suggest that someone should take a year off?   - **Number of Programs:**     - How many programs do applicants from our program in this subspecialty typically apply to?     - What are strong programs in this specialty, including academic and community programs?   - **Interviews:**     - Will interview be in person or virtual this year? - **Overall assessment**    - - What are the main factors that go into your ranking of an applicant?     - What do you consider negative qualities in a candidate?     - Do you recommend that we tell our number one program that they’re number one?   - **Preparation:**     - What electives do you recommend to improve my knowledge base for this field?     - What should interns and rising R2s do to prepare themselves to apply in terms of clinical and research opportunities? |
