# Supplementary material for: Development and Evaluation of a Five-Component Toolkit for Internal Medicine Residents Applying for Subspecialty Fellowships
Source: MedEdPORTAL. 2022 Mar 14;18:11228. doi: 10.15766/mep_2374-8265.11228 (PMC8918571; doi:10.15766/mep_2374-8265.11228)
Supplement: Supplementary file 1 — Elements of the Fellowship Application Toolkit.docxFellowship Application Guide.docxFellowship Application Information Night.pptxSubspecialty Breakout Room Questions.docxPreparing for Virtual Interviews.pptxMock Virtual Interview.docxSurvey Instrument.docx [file mep_2374-8265.11228-s001.zip › F. Mock Virtual Interview.docx]

**Mock Virtual Interview for Fellowship - Feedback Form for Faculty Interviewer**

Duration: 30 minutes (~20 minutes for the mock interview, ~10 minutes for feedback)

Sample Questions (Feel free to use your own, but please ask at least one "behavioral" question)

(1) Standard interview questions:

- What makes you interested in this subspecialty?
- Where do you see yourself in 5 to 10 years?
- How much research vs. clinical time do you anticipate in your career?
- What are you looking for in a fellowship program?

(2) Behavioral interview questions:

- Tell me about a difficult patient interaction you had and how you handled that.
- Tell me about a time that you had a conflict with a team member and how you handled that.
- Provide an example of constructive feedback you received and how you responded to it.

Please use this rubric for feedback and review with the applicant at the end of the interview:

| **Physical Space** | **Comments** |
| --- | --- |
| Camera angle is correct (face level, head centered) |  |
| Lighting is optimal (avoid back lighting) |  |
| Background is professional and not distracting |  |
| Noise level is minimal (including alert messages) |  |

| **Technology Set-up** | **Comments** |
| --- | --- |
| Internet connection is stable |  |
| Voice is clear and volume is appropriate |  |
| Name on virtual platform is correct |  |

| **Virtual Communication Skills** | **Comments** |
| --- | --- |
| Good posture (avoid swiveling or fidgeting) |  |
| Eye contact is with the camera |  |
| Hand gestures are visible and not off screen |  |
| Pace of speaking was not too slow or too fast |  |

| **Answer Content** | **Comments** |
| --- | --- |
| Level of detail was appropriate |  |
| Length of response was appropriate |  |
| Applicant was engaged/enthusiastic |  |
