# Supplementary material for: Development and Evaluation of a Five-Component Toolkit for Internal Medicine Residents Applying for Subspecialty Fellowships
Source: MedEdPORTAL. 2022 Mar 14;18:11228. doi: 10.15766/mep_2374-8265.11228 (PMC8918571; doi:10.15766/mep_2374-8265.11228)
Supplement: Supplementary file 1 — Elements of the Fellowship Application Toolkit.docxFellowship Application Guide.docxFellowship Application Information Night.pptxSubspecialty Breakout Room Questions.docxPreparing for Virtual Interviews.pptxMock Virtual Interview.docxSurvey Instrument.docx [file mep_2374-8265.11228-s001.zip › G. Survey Instrument.docx]

**Appendix G: Survey instrument (2020)**

1. Did you use the UCSF Fellowship Application Guidebook? (yes/no)
2. How many times did you refer to the UCSF Fellowship Application Guidebook?
   1. Once
   2. Twice
   3. Three-five times
   4. More than 5 times
3. How effective was the UCSF Fellowship Application Guidebook during the fellowship application process?
   1. Extremely effective
   2. Very effective
   3. Moderately effective
   4. Slightly effective
   5. Not effective at all
4. If you did NOT use the UCSF Fellowship Application Guidebook, what were the main reasons (check all that apply)?
   1. I did not know about it
   2. I did not know how to access it
   3. I did not think it would be helpful
   4. I did not find the information I was looking for
5. What were the strengths of the UCSF Fellowship Application Guidebook? [free text response]
6. What suggestions do you have to improve the UCSF Fellowship Application Guidebook? [free text response]
7. Did you attend the UCSF Fellowship Application Information Night? (yes/no)
8. How effective was the UCSF Fellowship Application Information Night during the fellowship application process?
   1. Extremely effective
   2. Very effective
   3. Moderately effective
   4. Slightly effective
   5. Not effective at all
9. If you did NOT attend the UCSF Fellowship Application Information Night, what were the main reasons (check all that apply)?
   1. I did not know about it
   2. I was not able to attend due to my call schedule
   3. I did not think it would be helpful
   4. I was not able to attend due to a personal obligation or vacation
10. What were the strengths of the UCSF Fellowship Application Information Nights? [free text response]
11. What suggestions do you have to improve the UCSF Fellowship Application Information Nights? [free text response]
12. Did you use the UCSF Young Alumni Contact List? (yes/no)
13. How many times did you refer to the UCSF Young Alumni Contact List?
    1. Once
    2. Twice
    3. Three-five times
    4. More than 5 times
14. How effective was the UCSF Young Alumni Contact List during the fellowship application process?
    1. Extremely effective
    2. Very effective
    3. Moderately effective
    4. Slightly effective
    5. Not effective at all
15. If you did NOT use the UCSF Young Alumni Contact List, what were the main reasons (check all that apply)?
    1. I did not know about it
    2. I did not know how to access it
    3. I did not think it would be helpful
    4. I did not find the information I was looking for
    5. I already had contacts who are current or recent fellows at other institutions
    6. I did not apply to fellowships at other institutions
16. What were the strengths of the UCSF Young Alumni Contact List? [free text response]
17. What suggestions do you have to improve the UCSF Young Alumni Contact List? [free text response]
18. Did you participate in the UCSF Virtual Personal Statement Workshop? (yes/no)
19. How effective were each of the following elements in helping you to write your personal statement:

- Repository of personal statements (e.g., examples from prior applicants)
- Tip sheet for personal statements
- 1:1 review with a personal statement coach
- Overall workshop effectiveness
  1. Extremely effective
  2. Very effective
  3. Moderately effective
  4. Slightly effective
  5. Not effective at all

1. Please rate how much you agree or disagree with the following statements about your overall experience participating in the Virtual Personal Statement Workshop:

- The Virtual Personal Statement Workshop helped me write a stronger personal statement
- The Virtual Personal Statement Workshop helped me better articulate my fellowship/career goals
- Participating in this workshop reduced my stress/anxiety about writing my fellowship personal statement
- Participating in this workshop provided a timeline to help me finish my personal statement
- The edits/advice that I received from my Coach improved my personal statement
- I would have preferred a Coach in my subspecialty
- I would recommend this workshop to residents applying next year
  1. Strongly agree
  2. Somewhat agree
  3. Neither agree nor disagree
  4. Somewhat disagree
  5. Strongly disagree

1. If you did NOT participate in the Virtual Personal Statement Workshop, what were the main reasons (check all that apply)?
   1. I did not know about it
   2. I did not think it would be helpful
   3. My personal statement was not ready in time
   4. I had other mentors/colleagues that were willing to read my personal statement
   5. Other
2. What were the strengths of the UCSF Personal Statement Workshop? [free text response]
3. What suggestions do you have to improve the UCSF Personal Statement Workshop? [free text response]
4. Did you attend the UCSF Virtual Interview Workshop? (yes/no)
5. How effective was the Virtual Interview Workshop in helping you prepare for virtual interviews in the following ways:

- Preparing you for interviews generally
- Preparing you for virtual interviews
- Preparing you for post-interview communication
- Overall effectiveness
  1. Extremely effective
  2. Very effective
  3. Moderately effective
  4. Slightly effective
  5. Not effective at all

1. If you did NOT attend the Virtual Interview Workshop, what were the main reasons (check all that apply)?
   1. I did not know about it
   2. I was not able to attend due to my call schedule
   3. I did not think it would be helpful
   4. I was not able to attend due to a personal obligation or vacation
   5. Other
2. What were the strengths of the UCSF Virtual Interview Workshop? [free text response]
3. What suggestions do you have to improve the UCSF Virtual Interview Workshop? [free text response]
4. Did you participate in the virtual mock interviews? (yes/no)
5. Please rate how much you agree or disagree with the following statements about your overall experience participating in the Virtual Mock Interviews:

- I received helpful guidance about my Zoom background or presence on camera
- I received helpful guidance about my responses to interview questions
- The virtual mock interview helped me prepare for my actual virtual interviews
- I had less anxiety about virtual interviews after this experience
- I would have preferred to have a mock interviewer in my subspecialty
- I would recommend mock interviews (virtual or in person) to applicants applying next year
  1. Strongly agree
  2. Somewhat agree
  3. Neither agree nor disagree
  4. Somewhat disagree
  5. Strongly disagree

1. If you did NOT participate in the virtual mock interviews, what were the main reasons (check all that apply)?
   1. I did not know about it
   2. I did not think it would be helpful
   3. I had other mentors/colleagues who were willing to do mock interviews with me
   4. Other
2. What were the strengths of the virtual mock interview experience? [free text response]
3. What suggestions do you have to improve the virtual mock interview experience? [free text response]
4. How effective was our residency program at advising you about the following aspects of the fellowship application process during the cycle that you applied to fellowship?

- The timeline of the fellowship application process
- Deciding which and how many programs to apply to
- Completing the ERAS application
- Writing a personal statement
- Scheduling interviews
- Post-interview communication
- Getting information about fellowship programs from current or recent fellows
- Providing subspecialty-specific information about the fellowship application process
- Overall effectiveness
  1. Extremely effective
  2. Very effective
  3. Moderately effective
  4. Slightly effective
  5. Not effective at all

1. Please indicate how much you agree or disagree with the following statements about the experience of applying to fellowship:

- The fellowship application process was stressful
- I felt alone in navigating the fellowship application process
- I felt supported by our residency program during the fellowship application process
- I felt confident that was I putting together the strongest fellowship application possible
- I knew who I could turn to if I had questions about the fellowship application process
  1. Strongly agree
  2. Somewhat agree
  3. Neither agree nor disagree
  4. Somewhat disagree
  5. Strongly disagree

1. What year were you when you applied to fellowship?
   1. R2
   2. R3
   3. One year after graduating from residency
   4. Two or more years after graduating from residency
2. Gender
   1. Female
   2. Male
   3. Non-binary
   4. Prefer to self-describe
   5. Prefer not to answer
3. How do you self-identify?
   1. Hispanic, Latino, or Spanish Origin
   2. American Indian or Alaskan Native
   3. Asian
   4. Black or African American
   5. Native Hawaiian or Pacific Islander
   6. While
   7. Other
   8. Prefer not to answer
4. What subspecialty did you apply to?
   1. Addiction Medicine
   2. Allergy/Immunology
   3. Cardiology
   4. Endocrinology
   5. Gastroenterology
   6. Geriatrics
   7. Hematology/Oncology
   8. HIV Primary Care
   9. Hospice and Palliative Care Medicine
   10. Infectious Diseases
   11. Nephrology
   12. Pulmonary/Critical Care Medicine
   13. Rheumatology
   14. Clinical Informatics
