# Supplementary material for: Development and Evaluation of a Five-Component Toolkit for Internal Medicine Residents Applying for Subspecialty Fellowships
Source: MedEdPORTAL. 2022 Mar 14;18:11228. doi: 10.15766/mep_2374-8265.11228 (PMC8918571; doi:10.15766/mep_2374-8265.11228)
Supplement: Supplementary file 1 — Elements of the Fellowship Application Toolkit.docxFellowship Application Guide.docxFellowship Application Information Night.pptxSubspecialty Breakout Room Questions.docxPreparing for Virtual Interviews.pptxMock Virtual Interview.docxSurvey Instrument.docx [file mep_2374-8265.11228-s001.zip › B. Fellowship Application Guide.docx]

**Internal Medicine Fellowship**

**Application Guidebook**

**Last Updated January 2022**

TABLE OF CONTENTS

[Introduction 2](#_Toc93830504)

[Important Websites 3](#_Toc93830505)

[Fellowship Application Timeline 4](#_Toc93830506)

[Cost/Finances 7](#_Toc93830507)

[Short Tracking 8](#_Toc93830508)

[Getting Information about Programs 10](#_Toc93830509)

[Letters of Recommendation 11](#_Toc93830510)

[Personal Statement 14](#_Toc93830511)

[ERAS Application 16](#_Toc93830512)

[Interview Day 18](#_Toc93830513)

[Post-Interview Communication 24](#_Toc93830514)

# Introduction

Dear Residents,

Welcome to the **Internal Medicine Subspecialty Fellowship Application Guidebook**! The key to success in the fellowship application process is thoughtful preparation, so this guide will walk you through the process.

There are several important caveats to state up front. First, while much of the information about the fellowship process is generalizable, it is important that you tailor any of these suggestions toward **your** specific needs and goals. Second, we have attempted to include accurate dates and websites, but please be aware that changes occur annually and you are ultimately responsible for your application content and its timeliness so please double check all application deadlines. Third, this guide provides timeline/application tips for ACGME fellowships, but does not specifically address the timeline for non-ACGME fellowships. Therefore, for non-ACGME fellowships, please refer to program websites or contact the UCSF program directors to learn more about their application process. Finally, the COVID-19 pandemic continues to impact the fellowship application process, and it remains to be seen how it will impact this cycle. We do not yet know whether interviews will be conducted virtually, in person, or a combination, but we will work with you to prepare no matter the final format.

We are here for you throughout the process and feel free to reach out with any questions. Best of luck in this exciting journey – you will do great!

Sincerely,

Laura Huppert, UCSF Hematology/Oncology Fellow

Jen Babik, UCSF Internal Medicine Residency Associate Program Director

Rebecca Berman, UCSF Internal Medicine Residency Program Director

# Important Websites

**ERAS/NMRP resources:**

- **ERAS:** Residents apply to most medicine subspecialty fellowships through the Electronic Residency Application Service (ERAS). ERAS is the electronic platform to complete and submit your application.

<http://students-residents.aamc.org/training-residency-fellowship/applying-fellowships-eras/>

- **ERAS Fellowship Documents Office (EFDO):** The site where you upload your transcript, LORs, etc.

<https://www.erasfellowshipdocuments.org/>

- **ERAS Tools:** This site has a user guide, timeline, applicant checklist, and other helpful resources. <https://students-residents.aamc.org/training-residency-fellowship/applying-fellowships-eras/tools-fellowship-applicants/>
- **NMRP:** Applicants must also enroll in the “Match” or the National Resident Matching Program/Subspecialty Matching Services (NMRP/SMS). This is not open when you first submit ERAS, but instead it opens in the late summer/early fall. You use this system to complete and certify your rank list in November, and this is the organization that tells you where you match. <http://www.nrmp.org/intro-fellowship-matches/>

# Fellowship Application Timeline

**PGY-1**

**June - December**

- Enjoy the beginning of intern year and focus on learning clinical medicine! As you admit patients and call consults, think about which subspecialties (if any) are appealing to you.
- If you are very excited about a subspecialty or project, reach out to potential mentors, but it is not expected at this point in the year.

**January - June**

- - Attend the Fellowship Application Information Night in Jan/Feb to get a sense of the fellowship application process if you are potentially interested in applying to subspecialty fellowship in the future. Interns are welcome and encouraged to attend!
  - Start thinking about mentorship/scholarly opportunities during the second half of intern year. Meet with your advisors or other faculty members to discuss your interests and help you formulate a list of faculty members in your field of interest to contact. Send an email introducing yourself, stating your interest in the field, and asking to meet to discuss research opportunities. Ideally set up meetings during the spring of your intern year so you have a tentative project set up by the beginning of R2 year.
  - Scheduling: Consider what inpatient and elective experiences will be helpful to make fellowship decisions and plan your R2 schedule accordingly.
  - If you have significant research experience (e.g. molecular medicine residents or other residents with significant research), you can consider short tracking and applying to fellowship at the end of your intern year. See separate section about short tracking in this document. Meet with your mentors in Jan/Feb to discuss whether this is the right decision for you.

**PGY-2 (or year in which you will apply)**

**June - December**

- - Get involved with scholarly activities!
  - If possible, it is helpful to submit an abstract or other scholarly piece by the end of R2 year or early R3 year. Scholarship takes many forms, including not only clinical/basic research, but also case reports, clinical vignettes, perspective pieces, review articles, letters to the editor, clinical images, etc. While this is an ideal goal, please keep in mind that many residents successfully apply to fellowship without completing significant scholarly work during residency: the need for this really depends on your individual application and your intended specialty.
  - In addition to core residency requirements and research, other aspects of professional development to cultivate during R2 and R3 years are leadership, citizenship, and advocacy.
  - Consider which faculty members may be able to write you strong letters of recommendation.

**January - March**

- - Attend the Fellowship Application Information Night in Jan/Feb. This evening event will provide an overview of the fellowship application process and an opportunity to ask subspecialty-specific questions during the subspecialty break-out sessions.
  - Reflect on your goals. Meet with your mentors to decide if you want to apply to fellowship this cycle.
  - Draft a personal statement and CV (see more on these topics later in this document).

**April**

- Update your personal statement and CV to provide to your letter writers.
- Formally meet with your residency program director to get your program letter.
- Request letters of recommendation from your other letter writers.
- Arrange a meeting with your subspecialty mentor +/- the fellowship program director.

**May**

- Finish asking for letters of recommendation by **May 15^th^ at the latest**. ERAS will not be open at this point, but let them know that you will follow-up with an individual Letter Request Form which provides instructions about how to upload the letter. Ask your writers to submit the letter by **June 20^th^ (or at the latest July 6^th^)** so that there is time for the letters to be processed. After the letters are submitted, it takes five additional days for them to be certified and released.
- Get a head shot photo taken if you don’t have one already (not a cropped vacation head shot!).

**June - July**

- Finalize your fellowship program list by talking with mentors, co-residents, alumni, etc (see the section on Getting Information on Programs later in this guide).
- **Register for ERAS and complete your application** **– usually opens around June 6th** (also see ERAS section of this document)
  - Confirm that you have an active AAMC account.
  - Purchase a valid ERAS token from the current season from the ERAS Fellowship Document Office (EFDO) (June 7) <https://www.erasfellowshipdocuments.org/>
  - Register for ERAS (June 7) <https://students-residents.aamc.org/training-residency-fellowship/applying-fellowships-eras/>
  - Contact your medical school to upload medical school transcripts and your Medical Student Performance Evaluation (MSPE).
  - Send your letter writers the individual letter request forms with upload instructions. Ask them to submit by late June (or at the latest July 6^th^) so that there is time for the letters to be processed.
  - Complete, submit and upload your MyERAS application, personal statement, and photo. The goal is to complete the entire application by **July 6^th^, at the very latest July 14^th^**. Applications will be released to July cycle fellowships on July 21^st^.
  - Check off everything on the ERAS fellowship applicant checklist! <https://aamc-orange.global.ssl.fastly.net/production/media/filer_public/10/4c/104cc6f5-86e1-4391-af8e-0273bbb7a5a4/aamc-eras-fellowship-applicant-checklist.pdf>

**PGY-3 (or year in which you will apply)**

**Late August**

- Interview invitations will begin to arrive. Try to schedule interviews during your outpatient/block time. Let your clinic know ASAP if you need to miss clinic
- Prepare for interviews (see interview section of this document). We will also host an interview preparation session and offer mock interviews with faculty members.
- Stay in touch with your advisors/mentors/program leadership during the process.
- Apply for NRMP Match in late August. Of note, this is a separate process that must be done IN ADDITION to ERAS: <http://www.nrmp.org/intro-fellowship-matches/>

**August - Early November:**

- Interviews

**Mid November:**

- Input and certify your rank list using the NMRP system (not in ERAS)

**Late November - Early December:**

- Match Day!
- Don’t forget to let your mentors/letter writers know where you matched and to write thank you notes!

**Other fellowship application timeline FAQs:**

Can I take a year "off" from training? Does it matter what I do during that time?

- Some residents take a "gap year" prior to applying to fellowship, usually to work as a general or specialty hospitalist (e.g. nocturnist, hospitalist, BMT hospitalist, CHF hospitalist, etc) or be a Chief Resident. This is now quite standard and does not usually raise any questions in terms of commitment to subspecialty training. If you choose to take a year away from training, try to stay involved in academic projects to demonstrate your commitment to the field and so you can talk about them during your interviews.
- If you want to take a year away from academia (e.g. to travel or work in private practice), talk to your mentors ahead of time about how this would fit into your application narrative.
- It is less common to take more than one year away, but it has been done for particular circumstances (e.g. to work at the Indian Health Services for two years, to align timing with a significant other); we recommend that you talk to your advisors if you are considering multiple gap years.

Should I delay my application for a year to do more research to strengthen my application?

- This is very specialty and program dependent. If your career goal is research or you are applying in a more research-heavy field, then it is advisable to have some kind of publication or abstract/poster to show that you can take a project to completion. The importance of this being a manuscript (versus a poster) or a first-author publication (versus middle author) is dependent on the field and the programs that you are applying to. As mentioned above, many residents successfully apply to fellowship without significant scholarly output during residency: the need for this really depends on your individual application and your intended specialty Talk to your career mentors to discuss the specifics of your application and this decision.

# Cost/Finances

Applying to fellowship can be expensive, particularly if interviews are conducted in person and require domestic travel (TBD for 2022 cycle). Here are some of the costs to consider:

**If virtual interviews only:**

** Fees below subject to change in 2022*

- ERAS token $105
- ERAS fee for first 10 programs $99
- ERAS fee per program, 11**-**20 $17 each
- ERAS fee per program, 21-30 $21 each
- ERAS fee per program, 31 or more $26 each
- Med school transcript/letter processing fee: Variable
- USMLE transcript fee $80
- NRMP/SMS match fee $75
- Interview clothing: Variable

**If in-person interviews:**

- Same expenses as above, plus interview travel expenses
  - Flights: Variable
  - Car rental: Variable
  - Hotels: Variable

**Advice from 2018/2019 fellowship applicants (in-person interview years):** *How much did you spend in total on applications and interviews? Any advice about how to finance this process?*

- $300ish. I didn’t travel (only applied locally), but spent about $300 in fees.
- $1500 (applied to seven programs). Not much advice, just to stay with friends in other cities!
- $2000 (or more... applied to 11 programs). I would try to group your interviews as much as you can so you are not going back and forth across the country.
- $2000-3000 (applied to 12 programs).
- Budget accordingly if needed in the months prior to interviews- I spent a lot of money in Sept and Oct and was glad I had planned to have extra funds available!

# Short Tracking

**What does “short tracking” mean?**

- If you have significant research experience (typically MD-PhD, multiple years of research prior to residency, or the equivalent), you can consider short tracking into a subspecialty by applying at the end of your R1 year and completing two years of internal medicine training instead of three years. Please note that short tracking does not "shorten" your overall training, but in general allows you to exchange the third year of IM residency for an additional research year in your fellowship. The ABIM refers to short tracking as the "ABIM Research Pathway." Please see here for additional requirements for the ABIM Research Pathway, including requirements by subspecialty and implications for changing out of the research pathway once you are in fellowship: <https://www.abim.org/certification/policies/research-pathway/policies-requirements.aspx>

**Who usually short tracks?**

- Typically, residents who short track are those with significant research experience. This includes residents with a basic science background as well as those with significant research experiences in other fields (e.g., significant research in health policy, epidemiology, clinical research, etc.). If you are unsure whether your research experience is “enough” to consider short tracking, talk to your mentors.
- Whether or not to short track is a very individualized decision. Many research-bound residents (with successful lab careers) have decided not to short track for various reasons, including uncertainty about subspecialty choice, lining up fellowship timing with a partner, uncertainty about future research plans, or a desire to complete 3 years of IM training. Please talk to your mentors about this decision.

**Are there requirements for short tracking in our residency program and when will I know if I am able to short track?**

- In general, in order to be eligible to short track you need to be meeting or exceeding milestones in all 6 competencies (medical knowledge, patient care, practice-based learning, systems-based practice, interpersonal and communication skills, and professionalism). If your evaluations suggest concerns in any of these domains, or you are not sure about this, please talk to your mentors as soon as possible.

**How does short tracking affect the timing of my Internal Medicine Board Exam?**

- If you choose to short track, you cannot take your IM Boards until you have completed three years of training in total. This means that you will take your IM Boards *after* your first year of fellowship, once you have completed two years of IM Residency and one year of Fellowship training,

**When do I have to decide whether I want to short track?**

- If you are considering short tracking, please talk to the residency program director by Feb/March of your intern year.

**If I am short tracking, how does this impact my fellowship application?**

- **Most of the advice in this guide still applies, with two notable exceptions:**
  - Letters of recommendation: Typically, your research letter is from your med school/PhD mentor rather than a residency research mentor. Your clinical letter may be from an individual in your subspecialty of interest, but it is also ok for a clinical letter to come from a hospitalist or clinic preceptor instead if you have not had the opportunity to work clinically with a subspecialist in your field during intern year.
  - Research/Experiences: The research/experiences you discuss in your application will likely be those from your MD or PhD only, rather than during residency. It is not generally expected that you will have any new research or extracurricular work from residency, since fellowship programs know you don’t have as much time during your intern year!

**Advice from a 2018-2021 fellowship applicants:**

- For short trackers, make sure to identify letter writers early. You can and should carry over at least one letter from your residency app, most likely a research mentor's (PhD advisor or other). Just reach out to that/those mentor(s) and ask them if you can update it for you. However, you most likely will need to identify at least one other non-departmental letter. If you are short tracking and already have a guaranteed spot here, it really doesn't matter who does this, but probably better if it's someone who knows you somewhat well (e.g., an attending you've worked with for at least 2 weeks, or a research mentor if you're a super-person and have started on this already in residency). You will likely have to ask, at the very latest, by 3/4 the way through *intern* year. So, keep your eyes out early, and ask that person as soon as you finish working with them, when they remember you best.

# Getting Information about Programs

**How do you choose what programs to apply to?**

- This is an integrative process that incorporates a number of personal and professional factors. Some common considerations include:
- Personal
  - Geography
  - Family considerations (proximity to family, spousal/partner employment, cost of living)
  - Characteristics of your application and personal/professional career goals
- Professional
  - Program focus (clinically focused, academic/research training, clinician-educator pathway, etc.)
  - Clinical experience (unique patient populations, procedural exposure, etc.)
  - Sub**-**subspecialty focus
  - Potential mentorship
  - Culture of the program
  - Competitiveness of the field that you are applying to
- We generally recommend starting by looking at program websites, talking to your mentor/advisor, and talking to recent residency grads in your field (see the young alumni contact list!)
- Another useful resource is FREIDA (Fellowship and Residency Electronic Interactive Database): <https://www.ama-assn.org/life-career/freida>
- When evaluating a program, consider the following questions:
  - What are the training and research opportunities for fellows at your program?
  - Is funding guaranteed for fellows or competitive? What is the set up for training grants at your institution?
  - Are there opportunities for further course work, methodological training, certificate or degree programs, etc.? How are these opportunities funded? (e.g., does the program pay for Master’s degrees for fellows?)
  - What is the clinical and research mentorship structure?
  - Where do graduates of their program end up for their careers?

**How many programs should I apply to?**

- This is also a highly personal decision, which requires you to consider the factors above and also the following: How competitive is my application? How competitive is my desired subspecialty? Do I have any geographic or personal limitations?
- Often it is helpful to talk to residents who applied in the cycle before you to get a sense of how many programs they applied to, and then your mentors.

**Advice from 2018-2021 fellowship applicants:** *Where did you get the best advice during this process?*

- Subspecialty mentor for sure. Also our APDs.
- The UCSF Fellowship program director and new fellows were extremely helpful.
- I talked to lots of friends who are currently fellows! Also talk to your co-residents who are applying--it is not a competition, you all want different things so use each other as resources!
- I talked to A LOT of people - former fellows and other program directors
- Talk to a mentor within the field who can give you honest advice. Talk to lots of former UCSF residents who are current fellows.
- Make connections early in your department and use them—not necessarily formal phone calls/emails but just knowing some names can go a long way. Reach out to Dr. Berman—she is your advocate in this process.
- Current fellows are an invaluable resource – ask them about their experience applying, programs they liked, how many programs they applied to, etc.

# Letters of Recommendation

**What types of letters of recommendation do I need?**

Every program will require three letters of recommendation (LOR) and some programs will accept a fourth. Here is what we recommend:

1. **Program Director letter (required)**: Required for all applicants.
2. **Clinical letter from a faculty member in your field of interest (essential):** Consider a faculty member you worked with in clinic, on consults, or on the wards in your area of interest. It is better to get a LOR from a faculty member who you worked with during R2 or R3 year, but a stellar intern letter is ok if needed. If you are short-tracking, it is expected that all of your letters will come from intern year.
3. **Research mentor (essential if research is a significant component of your application)**: If you performed significant research during residency, it is standard to have your mentor write a letter of support. If you worked very closely with a research mentor, the absence of a letter from this mentor may raise questions. If most of your research was conducted *before* residency, then consider the quantity/quality of research: for example, if you earned a PhD, then a letter from your PhD mentor is standard, whereas a medical school research letter is not necessary for less significant research endeavors.
4. **Fourth letter (optional, but encouraged):** The fourth letter can be another clinical letter OR a letter that highlights a different skill set (i.e., leadership, QI, etc.). If you get another clinical letter, this can be from another faculty member in your field of interest or from someone outside your field (e.g. a hospital medicine ward attending or ICU attending). Choosing between these will depend on who you think can write you a strong letter and also your subspecialty, as some subspecialties put more emphasis on having multiple subspecialty letters. See below for more discussion on three vs four letters.

**Who should write my letters?**

- After you review the letter requirements for your programs, think about who can write meaningful LORs about your clinical/research abilities, rather than someone who will rehash your CV. Since some measures of performance can be subjective (e.g. clinical judgment, research potential, intellectual curiosity), a strong letter of recommendation endorsing your knowledge, skills, and attitudes can carry considerable weight.
- We recommend that you make a list of potential letter writers and review it with your subspecialty mentor. These individuals may be able to provide insight into which writers have a track record of writing strong letters of recommendation.
- It is often difficult to know whether an enthusiastic letter from a junior faculty is more or less valuable than a less-personal letter from an esteemed faculty member. This preference may differ across specialties, so ask your subspecialty-specific mentors for advice here if you are not sure.

**How many letters?**

- There are no rules for deciding on three vs. four LORs unless an institution specifically states a preferred number. Generally, we recommend getting four strong letters if possible.
- Please note: A few programs cap the number of LORs that can be submitted! ERAS does not consider these caps and allows you to submit up to four LORs and then the program randomly reads three of them. So if the program has a cap, abide by the rule and only assign the three most important letters to that program.

**When should I ask for letters?**

- Ask for your letter by early May, and by **May 15th AT THE LATEST**. This gives your writers more than a month to write your letter and get it uploaded. Request that they submit the letter by June 20^th^ (July 6^th^ at the latest) so there is time for the letter to be certified and released to ERAS.

**How do I ask for a letter?**

- If possible, try to arrange a brief **in-person/zoom meeting** to ask your potential letter writer for the letter, but also okay to do this over email if needed.
  - Make sure to first ask something like “Do you know me well enough to write me a strong letter” or “do you feel comfortable writing me a strong letter for my fellowship application?” This gives the faculty member an “out” if they don’t feel like they can (for whatever reason) write you a strong LOR and saves you from a potentially sub-optimal letter.
  - Meeting in person allows you to refresh their memory about their experience with you and make sure they are up to date with your life, career goals, etc. This not only works toward giving your entire application more thematic coherence (i.e. your LOR authors are not saying different things about your anticipated career pathway) but also generally leads to a more personalized letter.
  - When you meet with them (or email them) provide them with:
    - Personal statement draft
    - CV
    - A descriptive summary of any relevant research experience
  - Let your letter writers know that the letters should be submitted by late June and that you will send them upload instructions in June once ERAS is open.
- ERAS will reject letters that mention a specific program. Ask your letter writers NOT to mention names of programs specifically, even if you have a clear top choice.
- Follow up via email in early June to provide the ERAS upload instructions.
- Follow up in mid-June before the letter is due to give them a polite reminder.
- Send a thank you note afterwards to all letter writers.

**What if I am asked to draft a letter for myself?**

- Occasionally, faculty members ask you to draft a letter of recommendation for yourself, which can be a daunting task. The best thing to do in this situation is to make sure *you* write yourself a strong letter – the tendency is to undersell yourself when you are writing your own letter so you have to overcome that!
- One strategy is to have a colleague/friend review it to make sure it is strong enough. Another strategy is to look at that person’s evaluation of you in the past, if you have it, and use their own words in the letter.
- When you email it back to the letter writer, it is ok to acknowledge “I was advised to write the strongest letter possible for myself so please feel free to change any of the adjectives or edit it liberally”. He/she will hopefully edit it in a lot of detail. This person probably wants you to fill in all the details about yourself and the rotation, etc. But in the off chance he/she doesn’t, you want the letter to be as strong as possible.

**How do I upload my letters of recommendation to ERAS?**

- You will need to use ERAS to generate a Letter Request Form for **EACH** letter writer that includes instructions for the Letter of Recommendation Portal for your letter authors. Email this form to your letter writers. Letter Request Forms should not be duplicated and distributed to multiple LOR Authors. They are customized for each letter and contain a unique Letter ID that can only be used once.
- When you complete your ERAS form, it is standard practice to **waive** your right to review your letter. This ensures your letter writers will be as honest as possible, and the writers will include a standard phrase (e.g., "Laura has waived her right to review this letter...") in their LORs.
- You should ask your letter writers to write a LOR on your behalf in May, and then let them know that you will send upload instructions in early June when it is available.
- Don’t forget that even after a LOR has been uploaded, **YOU must assign it** to any individual program before it will be released to the program.

**Advice from 2018-2021 fellowship applicants:** *Any advice about letters of recommendation?*

- Don’t be afraid to ask. People like to help. If someone has given you particularly positive feedback, that’s a good person to ask and let them know why you think the program will benefit from hearing from them so they know what to include.
- I applied to fellowship very last minute but luckily already had a letter that had been drafted by our program director for a different purpose, and was easily adapted for this. It's helpful if you have in mind who you want to write letter just in case something unexpected comes up!
- Letters from higher-ranking faculty are more valued than from junior faculty (as long as you think they will still be strong), in cardiology at least.
- Because my advisor knew I was mainly interested in one program, he addressed the letter to that program but I ended up applying to two programs- fortunately, ERAS rejected the letter but the process was stressful. It is probably better that advisors are always under the impression that you may investigate several programs and don’t list program specifics in the letter. If you want to add program specifics you can do that by uploading separate personal statements if it is really important, but that is not true for the letters.
- Ask in May and give a June deadline even though it isn't actually due until July. Think about quality of the letters more than prestige from your letter writer.

# Personal Statement

**Personal Statement Tips**

- **Main goals:** The main goals of the fellowship application personal statement are to convey (a) why you are interested in the specialty, (b) what you have done to prepare yourself for practice in the specialty, and (c) what you are interested in doing in your career and what you want to get out of fellowship.
- **Structure**: The structure of the PS should reflect the above goals. Most personal statements will follow the outline below. Limit your PS to one page if possible

Paragraph 1 (Introduction):

- Why are you interested in the specialty? The PS often starts with a case or personal anecdote but this should be brief (shorter than what is usually done for a residency application). Be sure to mention your interest in patients (not just pathophysiology or research).
- Also include here any personal narrative that won’t be obvious in your application – distance traveled, contribution to diversity, etc.

Paragraph 2 (Clinical Experiences)

- What were the clinical experiences in residency that helped affirm this decision?
- If you had any other relevant experiences (e.g., volunteer experiences, service, leadership, etc.), this is a good place to include that.
- Include experiences in med school (or other) if very relevant or significant

Paragraph 3 (Research/Scholarly Activity)

- What research or other scholarly activities did you do during residency that have prepared you for subspecialty practice and influenced your career path?
- Include experiences in med school (or before) if very relevant or significant

Paragraph 4 (Career Plans)

- What do you see yourself doing in the future (research, clinical ,education, QI, etc)? It’s okay not to be totally sure but you should have some sense of where you are going (and remember, no one will hold you to this afterward). Choose your narrative and stick to it, even if things may change down the line.
- What do you want to get out of fellowship (strong clinical training, research opportunities, mentorship)?

**Other personal statement advice?**

- **What the personal statement can do for you**
  - Let programs know why you are interested in their specialty, what you want in a training program, and what you ultimately hope to accomplish in your career.
  - Highlight major items from your CV.
  - Highlight life experiences or skills that may not be adequately reflected in your CV.
  - Explain any lapses in training, unusual circumstances, etc. It is often best to address these issues outright, but you should discuss this with your mentor first.
- **What the personal statement can do for the program**
  - Assist an interviewer in preparing targeted questions/”breaking the ice” for your interview
  - Allow them to see how you will fit in to their departments overall goals
- **What if I am interested in a career goal that is *not* research? Can I say this?**
  - This is highly dependent on the subspecialty and program. Some fellowship programs have clinical or clinician-educator tracks so it is clear that you can state these non-research goals explicitly. If you are unsure, talk to your mentors about the best way to frame your application to be a competitive candidate while staying true to your career goals. If you are concerned about the confidentiality of discussing this with a mentor.
- **Why can I upload multiple personal statements?**
  - ERAS lets you upload separate personal statements for each program. Usually you only need one personal statement and can upload the same one to all programs- this is the most common approach. In unique circumstances, you may consider writing a separate personal statement for your top program if you have a particularly compelling/unique reason that you hope to match there or if you are applying to a specific track within a program (e.g., a personal statement for a clinician-educator track might look different than your general personal statement). Be careful if you decide to write multiple personal statements when you upload them- make sure that you have assigned the correct personal statement to the desired programs.
- **Other general tips**
  - Use active voice/action verbs. Instead of saying “Directing a QI program at a cancer center would be a rewarding career pathway,” say “I hope to direct a QI program at a cancer center."
- Maintain a consistent story line (in your personal statement and throughout your application).
- Connect your thoughts and passions with concrete examples from your experiences- do not be vague. In general, depth is better than breadth.
- It is good to talk about issues or experiences that have shaped you, but avoid controversial topics (e.g., avoid drama, religion, politics, etc.). It is better for your personal statement to "fly under the radar" than attract negative attention, so do not take risks with the narrative.
  - Ask friends and mentors to edit it to ensure that it is clear, organized, and error-free.
- **When should I start working on my personal statement?**
  - Start working on your personal statement in the spring before you apply. You should have a fairly complete draft by **late April /** **early May** to provide to the individuals who write your letters.

**How do I upload my personal statement into ERAS?**

- We recommend that you do NOT just cut and paste your personal statement from Microsoft Word directly into ERAS because some special characters and formatting may change. Instead, paste it into a copy editor like Notepad and then into ERAS. Re-read it again in ERAS again before submitting.
- Then **YOU must assign your personal statement** **to all programs**

**Advice from 2018-2021 fellowship applicants:** *Any advice about writing an effective personal statement?*

- Think about where you want to be in 5 years first and work backwards to create a consistent story line or arch to your statement.
- Personal statement should truly allow the reader to get to know the applicant. This is the time to share background story, try to explicitly share why you chose a specific specialty and where you are heading.
- Keep the personal statement simple and direct so that it clearly communicates your career goals. No need for personal vignette or extended story like you may have done for med school/residency.
- It is important to have a “story” that weaves throughout your application including your personal statement and interviews. Even if you have seemingly random activities/interests, try to weave together into a more linear narrative for the personal statement.
- Have at least one mentor read over your PS to get at least a second set of eyes.
- It is helpful to see examples from other people who have applied about how to structure it.

# ERAS Application

**ERAS: What do I need to do again?**

- **ERAS FAQs:** <https://students-residents.aamc.org/training-residency-fellowship/faq/faq-eras-fellowship-applicants/>

**Specific ERAS Details:**

- Who do I put as my “program supervisor”? Your residency program director.
- What type of medical license do I have? Full

**Uploading my LOR and Personal Statements into ERAS?**

See the LOR and Personal Statement sections above for instructions

**Do I need to update my CV?**

- Yes, you should update your CV to provide to your letter writers, but you will never submit a Word/PDF version on ERAS. Instead you’ll upload the elements of your CV into the ERAS Experiences section (see more on this below).
- There are lots of CV examples and templates available; one resource is the AAMC: <https://www.aamc.org/members/gfa/faculty_vitae/150034/preparing_your_curriculum_vitae.html>

**What do I need to know about the Experiences section of ERAS?**

- In ERAS, you will input information into the experiences section and it will create a standardized CV for you, so you will never need to upload your actual CV. It looks similar to the ERAS you used to apply to residency – you can copy forward information from your residency info if you would like.
- Types of Experiences:
  - **Research**: Include projects from residency, med school, and college if relevant. For each research experience provide a brief 1-2 sentence summary of your responsibilities and what you gained from each experience. If it is listed here, be sure you are able to discuss it articulately.
  - **Work:** Include work experiences from college and medical school. Most people do not have additional work experiences from residency.
  - **Volunteer:** Not as important as for residency applications, but you should any major contributions from medical school and then activities done during residency (e.g. residency committees, volunteering at the homeless clinic, med school mentoring roles, etc.)
- When it asks “reason for leaving experiences”: You can leave this blank if there was a natural reason for leaving an activity, like finishing college or medical school. It is intended to address things you left early. When in doubt, just state a reason.

**What publications should I include?**

- Include all publications that have been published, are in press, or have been submitted (there is a way to specify these separately on ERAS). Do **not** include manuscripts in progress.
- Include poster presentations and oral abstracts in the relevant ERAS sections. If you have a lot of these from college/med school that are not relevant to your current subspecialty, it is ok to limit to the most important/largest impact conferences.

**How do I get my medical school transcript or Medical School Performance Evaluation (MSPE)?**

- You are also responsible for obtaining a copy of your medical school transcript and MSPE to be uploaded to the ERAS Fellowship Documents Office (EFDO). There are two mechanisms by which this can happen so please contact your medical school and ask which they prefer:

1. Medical Institution Document Upload Service (MIDUS) is a service that allows medical schools to directly upload your MSPE and transcript to the EFDO. In order for a medical institution to upload a document on your behalf using MIDUS, you must send an electronic request to the medical institution using EFDO Online Services via MyERAS. Most schools do it this way.
2. Your school of graduation may choose to release your MSPE(s) or medical school transcripts directly to you and you will have to scan and upload.

- It takes 2-4 weeks for many schools to upload these documents, so request them in early June!
- Once your medical school has uploaded these documents, you need to assign them to each program.

**How do I upload my USMLE transcript?**

- When you submit your ERAS application, you will check a box to “authorize” your USMLE transcripts to be released, and they will charge you an $80 processing fee at that time. You do not have to look on other websites or do anything else before you submit.

**What about the photograph?**

- A professional head shot is not necessary but it is advisable that the picture appears intended for the purpose of a professional application (i.e. avoid cropping from a recent vacation picture). Dress should be somewhere between a business suit to clinic-appropriate professional attire.

**Can I couples match for fellowship?**

- Yes you can! See here for more information: <http://www.nrmp.org/match-process/couples-in-the-match/>
- Couples matching can be more logistically complicated, so we recommend talking to your residency program advisors in advance.
- Consider geographic areas with several training program options.
- Fellowship PDs will often coordinate with each other in order to recruit you as a couple

**Supplementary Applications?**

- Make sure you check out the website for each program to see if they have a supplementary application. These are relatively uncommon.

**Why don’t I have the NRMP number before I apply to ERAS in mid-July?**

- You cannot get an NRMP number until late August, so leave this section blank in ERAS when you submit in July.

**Advice from 2018-2021 fellowship applicants:** *Any advice about ERAS?*

- You don't need to include all the stuff from med school or college (i.e. president of X club, coordinator for Y elective). Do include any research, work experience. I didn't include the social justice work I did in college but I probably could have.
- Get started VERY early. There are a bunch of things you might not anticipate that you need to get (like your Deans letter from Med school??) and it takes a long time for the system to process each submission.
- It takes time to fill in al review all the CV (activities), start early. You can also copy forward the activities from residency. Make sure to review writing style as character count requirements is different.
- It makes it much easier to fill out ERAS if you are updating your CV at regular intervals. So do so as you go throughout residency.
- Start early. Don’t apply to programs you realistically have little interest in attending.
- Start the process early! Some steps require verification from your med school which can take a while
- Have a fellowship application buddy. This can help make sure you have the right ERAS formatting and that you are not missing anything important during the process.
- Mark your calendar NOW with the date ERAS opens- you will not get a reminder so you need to be on top of it. You will want to send your LOR letter requests when it opens as soon as you can.

# Interview Day

**It is unclear whether interviews will be conducted virtually, in-person, or a combination for the 2022 application cycle – Stay tuned and we will certainly work with you to prepare no matter the final format!**

**How many days off can I take for interviews? What about missing clinic?**

- The average number of days that residents take off for fellowship interviews is 7-10 days at our program. Talk to your program leadership to learn their policies about missing residency days/clinic and how this will be covered.

**How do I arrange interviews?**

- Interview dates range from late-August to early November with most in Sept/Oct. Most programs have 4-8 interview days, but some only have one or two. Some programs schedule interviews in random order and getting slots early/late in the season isn’t a marker of your “desirability” as a candidate.
- It is better to schedule interviews as you get them, then reschedule if an unavoidable scheduling conflict arises (but don’t do this often, and ensure you reschedule at least two weeks in advance).
- Please let your subspecialty mentor know if you are declined an interview at a top choice program so that they can help advocate on your behalf.

**What is the purpose of the interview?**

- A means for the program to assess how well you’d fit in with their people, culture, vision, mentors (most interviewees will fall into the acceptable range) vs. those who wouldn’t fit (less common).
- A way for you as an applicant to learn about the program in person – try to gain insight about whether the fellows are happy and feel supported, research/scholarly opportunities and potential mentors, etc.
- A chance for the program to try to recruit you to join their fellowship.

**How do fellowship interviews differ from IM residency interviews?**

- Generally there will be fewer candidates applying and applicants will generally have a better sense of their specific career goals.
- As the applicant field is far smaller than for residency, expect that the interviewers will know much more about you and your accomplishments.
- You may interview with more faculty members during fellowship interviews than IM interviews.
- Depending on the specialty and program, some interviews really feel more like an opportunity for them to recruit you! Others may feel more like a true interview.

**Individual preparation and practice questions**

- General tips:
  - Think about concrete strengths in each of these categories:
- Scholarly work. If you haven’t thought about a project in a while, review the data and be able to speak eloquently about it. Anything on your ERAS application is fair game!
- Areas of clinical care which you particularly enjoy and why.
- Teaching that you’ve done that’s been rewarding
- Leadership roles you’ve had during medical school and residency (committees, etc.)
  - Be able to articulate why you’d be a great fellow (at this program or any other).
  - It is okay not to be 100% differentiated, but try to avoid too much indecision or lack of a career plan. You should at least have some sense of where you want to be in ten years. As per your personal statement, choose your narrative and stick to it, even if things may change down the line.
  - If there are any unusual aspects of your background (e.g. gaps in training, interesting research, travel, etc.), expect to be asked questions about these topics. You may even want to highlight these issues if you have good explanations for them.
  - Be very familiar with your field of interest – in particular new directions or challenges. Also know the buzzwords, especially as they apply to you (e.g., for research: T32, K award, R01, etc.)
  - Practice answering common questions such as those below. Learn how to pivot to your talking points - “Yes, good question. I think this relates to x, y and z…”.
- **Practice questions:**
- Tell me a little bit about yourself.
- What makes you interested in this subspecialty?  When did your interest in this field begin (i.e. any seminal events - clinical, research, other)?
- What will you be able to contribute to our program/community?
- Where do you see yourself in 5-10-15 years?
- What are you looking for in a fellowship program?
- Why are you interested in our program?
- What do you want to do during your research year(s)? Who do you want to work with at our program?
- How much research vs. clinical time do you anticipate in your career?
- What is an example of adversity that you have overcome?
- How has the COVID-19 pandemic affected your training?
- What are you passionate about outside of medicine?
- What was the last book you read?
- What are the greatest challenges facing our field now?
- What have been the greatest advances in our field?
- What are you passionate about outside of medicine?
- **Practice “behavioral questions:”**
  - Tell me about a time where you had a difficult patient interaction.
  - Tell me about a stressful situation you experienced in residency and how you handled it.
  - Tell me about a time you made a mistake and how did you handled the situation.
  - Tell me about a challenge in your life outside of medicine and how you addressed it.
  - Tell me about a time that you had a conflict with a team member and how you handled it.
  - How do you resolve conflict/Tell me about a conflict that you have resolved?
  - Provide an example of constructive feedback that you received and how you responded.
- **Questions that are NOT allowed:**
  - Your interviewer should NOT ask you about personal characteristics/family planning (e.g. sexual orientation, religion, whether you plan to have children, etc.). If you are asked, you can politely decline to answer or answer in whatever way you feel comfortable.
  - We hope that you do not encounter these questions, but good to consider what to do just in case. Here are some examples of inappropriate questions and how you could respond in the moment:
    - “I see that you are couple’s matching. Are you planning on having a family soon?” 🡪 possible response: “Yes, I am couple’s matching, but we are not sure about our family plans and this decision won’t affect my training.”
    - “I see that you are married. Do you want to have kids during fellowship?” 🡪 Actually since you ask that, what provisions does this program have in place in general if a fellow has a family or personal emergency or requires parental leave.
    - “What other programs are you interviewing at?” 🡪 I’m interviewing broadly to find the best fit for my clinical and research goals. I am very happy to be interviewing here today. How long have you worked at this institution and what do you like best about the culture here?
    - “Are you religious?” 🡪 My spiritual beliefs will not affect my training.
  - Please let your mentors know if inappropriate questions come up so that they can discuss how this should be addressed.

**Program-specific preparation**

- Know the nuts and bolts of the programs to which you apply. Insights can be gained by:
  - Reviewing their website - note major clinical and/or research strengths and key faculty.
  - See if any faculty, fellows or grads have gone to that program to ask them more about it.
- If you happen to know the interviewer’s name prior to the session (even 10 minutes), it might help to look them up quickly to gain a little insight about their background.
- Develop a list of potential mentors whose interests align with yours at that institution. You may be asked to provide a list of people you’d like to meet when you schedule the interview. If not and there is someone you’d particularly like to talk to, politely ask if there is a potential time to talk to her/him while you’re there or see if you can set up a phone meeting afterwards. In general, it is advisable to do some homework and know who you may want to work with at each institution.
- You will be asked, "What questions do you have?" Be prepared with at least three specific questions to ask throughout the day. This is your chance to show (a) you have insightful questions about the training program and (b) you have done your homework and are prepared for the interview. It is okay to repeat some of the same questions in your interviews. Examples of general questions:
  - What do you think are your program’s greatest strengths and weaknesses?
  - What are your plans for the program’s future? Do you anticipate any major changes in the program over the next few years?
  - What is the clinical rotation structure? What proportion of time is inpatient vs. outpatient? Is there a continuity clinic for fellows?
  - How is the research portion of fellowship structured?
  - How are the senior fellows funded? Do you have T32 tracks or are they funded through the department? Are senior fellows guaranteed funding or is their position dependent upon grant funding? How successful are fellows at getting career development awards?
  - What is the mentorship structure?
  - Are their opportunities to take courses or gain additional training in research methods?
  - Where do graduates take jobs? What percentage go to academia, private practice, or industry?

**General Interview day advice?**

- - - Don’t forget the interview BASICS:
      - Dress professionally- usually business attire. Know where you are going and get there early.
      - Be nice to everyone you meet. Anticipate that anyone with whom you interact (administrators, MDs, RNs, LVNS, residents, fellows, staff, etc.) will have an opportunity to comment on you and your behavior. Word travels quickly if someone is perceived as rude or arrogant.
      - Don’t fidget, look at your paperwork, check your phone or otherwise appear distracted.
      - Focusing on the call schedule and workload of the program can be perceived negatively, and it may appear that you are more concerned about call/scheduling details than learning about the goals of the program in general. Instead, consider asking these questions before or after the interview day, particularly if there are alumni you know and trust.
      - You will likely see or work with people from the interview trail during your career. Recognize that first impressions made during the interview process can have a lasting effect, even if you don’t go to that program. This is true of other applicants as well– they will be your colleagues soon!

**In person day advice?**

For in person interviews, they are typically structured with a social happy hour or dinner with residents the night prior and then a program overview, tour, and interviews during the actual interview day.

- - - Interview dinner:
      - Check the timing of this before scheduling flights or travel, as this may be the night before the interview day.
      - Try to attend this event if possible – it is often the best opportunity to speak to fellows and learn about the program from their perspective.
    - Interview day:
- Be prepared to have 4-10 interviews/day, depending on the program. Most interviews are 1:1 but occasionally there are panel interviews. It is OK to repeat yourself in terms of responses to questions if you have many 1:1 interviews.
- You are entitled to rights/accommodations on your interview days as needed. For example, for mothers who are breast feeding, you are entitled to ask the program coordinator for dedicated time/space for pumping during your interview day.

**Virtual Interview Tips?**

Here are six tips for how to specifically prepare for virtual interviews (also see Huppert et. al., *Journal of Cancer Education* 2021)

1. **Test your hardware**

- Use a reliable laptop or desktop with functioning audio/video
- Ensure you have a strong internet connection (ethernet preferred)
- Check the quality of your microphone in your computer vs. headphones
- If you do not have an adequate computer set up at home, ask your program
- Tech fails and glitches will still happen, don’t stress! Always have a cell phone back-up

1. **Test your software**

- Download the proper virtual platform
- Complete your profile with your name and a professional photograph
- Perform an audiovisual test using that software (can adjust manually for light)
- Note: Video conferencing platforms are often optimized for lighter skin tones, so those with darker skin tones may unfortunately need to go to extra efforts to set up lighting manually

1. **Choose your physical space**

- Background
  - Choose a space at home or on campus with a neutral, non-distracting background.
  - Tidy your surroundings and know what is visible
  - Virtual background generally not recommended. If you use one, choose a simple background or one provided by your home institution
- Close windows and doors to reduce noise
- Children, roommates, or pets at home? Make a plan for care to avoid distractions
- If you don’t have a good space at home, ask your program for a place on campus

1. **Optimize your physical set-up**

- Center your face in the screen with space above the top of your head
- Camera should be at eye level
- Place the interviewer near the camera so you are looking right at them when talking (drag and drop in Zoom)
- Turn off notifications on your computer and silence your cell phone
- Position lighting behind the camera, illuminating your face

1. **Practice interview questions**

- Review practice questions including behavioral questions [see example questions above]
- Practice mock interviews
- Record yourself
- Practice with a peer
- Practice with a mentor/faculty member

1. **Do your homework about each program**

- Research each program in advance, including:
- Structure of curriculum, clinical training, and research
- Strengths/weaknesses of the program
- Brainstorm questions that you want to ask
- Look up your interviewers in advance (if known)

1. **Ace the virtual interview day**

- Recheck your technology and physical set up – see Steps 1-4!
- Plug in your computer and check your battery
- Dress professionally, as you would for an in-person interview
- Be polite to everyone with whom you interact

1. **Gather information from the virtual “interview day”?**

- Ask questions to your interviewers and the program director
- Talk to current residents at virtual social events and/or contact them afterwards to learn more about the program 1:1
- Recreate a network of other applicants applying this cycle to compare notes
- Reflect and take notes after each virtual interview day

**What should I do immediately after the interview?**

- Write down your impressions. Try to cover the same ground for each program so that you can more easily make comparisons as the interview season rolls along.
- Reflect honestly on your performance and use this information to guide additional preparation.
- Pay attention to any vibe that the trainees aren’t happy and/or that a place isn’t a good fit for you. Try to identify precisely what doesn’t feel right. Circle back and ask more questions afterwards if needed.

**Advice from 2018-2021 fellowship applicants:** *Any advice about interviews?*

- Recommend creating bullet point answers to many of the common questions, that way you have a sketched out script of what to talk about. It helps not to have everything scripted out so it doesn’t sound canned, and gives opportunity for regular speech variation.
- Remember to create a consistent story about your experiences and goals. It is better to have a cohesive narrative, even if your plans might change a bit. You will have time to explore in fellowship! But best to try to have a clear story for the interview.
- I thought interviews were very helpful to get a sense of the programs, especially if you aren’t planning to rank based on prestige alone. If you have the time, go to that extra interview even if you aren’t sure about a certain program because it may surprise you.
- The interviews were very personal and allow to get to know potential mentors. If an interview makes you feel uncomfortable, it is probably a sign that the program is not the right program for you.
- This year they were all zoom interviews, so I'd suggest finding a cozy space at home to have them all. They are long so prepared to be at home the entire day.
- I wish I had explored possible research mentors at each institution before the interview day. This might have helped me make my decision because I could have setup meetings on the day of my interview.
- I was surprised by how time-consuming the interview process was! It feels like a full-time job so prepare to put your research/other projects on hold while you are applying particularly between Sept-October.
- Go to the pre-interview dinner. They were extremely helpful to get a sense of the ethos of the program. The real resource is talking to the fellows and your friends who interviewed the year before, they will tell you about all of the programs with some perspective. The interview trail can be fun, you see people over and over again you actually become friends with them.
- The only thing I wish I had known at the outset is that you can proactively reach out to “lower tier” programs if you have a particular interest in their program. Sometimes they don’t routinely offer interviews if they don’t think you will come there, so I wish I had reached out specifically to the PDs/APDs at that program early on and/or asked Rebecca and others to reach out on my behalf to express my interest in the program. I didn’t get an interview at one local program even though I was very interested and wanted to stay in the Bay Area, so I wish I had known that and maybe this would have led to a different outcome.
- Trust the process. Email programs yourself or have someone email on your behalf if you really want an interview somewhere (success rate with this tactic will be much higher with non top-tier programs). Try to get something published before applying if you really want to go to a top-tier program.

# Post-Interview Communication

**Program to Applicant communication**

- The Match is designed to discourage any communication from the program to the applicant after the day of the interview. Therefore, if you do not hear from the program, it does not indicate a lack of interest in you but instead that they may be believers in the purity of the Match system.
- However, post-interview communication happens MUCH more frequently in the fellowship interview process than for residency interviews. Fellowship programs are not prohibited from contacting you so you may get a call or an email, often just to check if you have any additional questions.
- Programs can tell you where you are ranked on their list but they cannot ask you how you are going to rank them - this is an NRMP Match Violation. Unfortunately, we’ve heard this occasionally still happens which can be stressful. If this happens to you, you can politely say that you loved visiting their program and are still deciding on your final rank list, and then take the opportunity to ask any other questions you may have. Do not tell a program they are #1 unless they are truly #1. Please let your mentor know if a program asks you for your rank list – with your permission, it is important for our program to anonymously give feedback to the NRMP after the match is over so that we can help protect future applicants and respect the match process.
- Example outreach and responses
  - Program: “We just want to touch base and see if you have any questions”. Example response: “Thank you so much for reaching out. All of your faculty and fellows were so informative during the interview day that I don’t have any questions right now. I will definitely reach back out though if any additional questions come up.”
  - Program: “We are planning to rank you to Match.” Thank you so much for this email. I am truly honored to be considered so highly by your program. I was very impressed with your fellowship and all that it has to offer. I am still finalizing my rank list and will certainly reach out if I have any additional questions about your program.” [If that last sentence isn’t true, you can cut it.].

**Applicant to Program communication**

- Post-interview communication can be tricky. Don’t be afraid to ask for help! We are all here for you!!!
- It is fine to send a simple thank you note or e-mail to the Program Director or any of your interviewers, but this is not usually required (see advice from applicants, below).
- If there have been major updates to your application (e.g. a major publication), it is ok to let the program know the update to add to your file.
- Consider sending communication to your top program telling them your intention to rank them #1. This is not a requirement. Some programs may use this information to alter their rank list but most programs do not. Importantly, only tell a program that you are ranking them #1 if you actually are going to do so. Dishonesty will reflect poorly on you and may follow you in the future as the fellowship world is a small community and program directors talk to one another. Before you tell a program that they are #1, talk to your mentors and make sure you are sure about this decision.
- It is usually not necessary to tell the other programs near the top of your list (#2 and #3) that you have "ranked them highly" because they know that means you are not ranking them number 1. It probably doesn't hurt, but it doesn't really help either.

**Rank List**

- After talking to your mentors, loved ones, contacts from your residency program who trained at the fellowship program, etc… make a rank list and submit it in mid-November!
- Pay close attention to the NRMP program number, as some programs have multiple numbers listed for different research tracks within the program.

**Taking a position outside of the Match**

- Programs in some specialties are allowed to take applicants outside of the Match. This is rarely done, but it does happen especially when smaller programs are trying to secure top candidates.
- If you are offered a position outside of the Match, we recommend that you discuss this ASAP with your subspecialty mentors and residency leadership to make sure that all critical issues are addressed before formally accepting the position.

**Bottom line:**

- You are terrific applicants and have so much to offer to fellowship programs. Your subspecialty mentors and advisors are all here to help during the application process, so don’t hesitate to reach out. You will do great!!!

**Advice from 2018-2021 fellowship applicants:** *Any advice about post-interview communication and creating your rank list?*

- I still briefly sent thank you emails to all of my interviewers. It's unclear if you need to do this, though.
- My mentor recommended letting the first choice program know your intentions. This is a bit different than the direction IM match is going so good to know. If a program contacts you letting you know you’re high on their list, but they’re not your first choice, write back letting them know all the reasons you agree their program would be a great match for you. Avoid making it sound like you are ranking them first too. Programs want people who will be happy at their program, so just show them why you would be even if it’s not your first choice.
- I would email your number 1 to tell them. I decided not to email 2 and 3 but it's really a toss up because if you do email them and don't say they are your number 1, they will know it. I did however do thank you notes (emails) after the interviews to each of my interviewers which was definitely time consuming but I thought that it made a difference (and the UCSF cardiology fellowship leadership recommends this). As for ranking your final list--unlike residency I would think more about the location and your personal life. Also think about call schedule and what program has the best balance of your personal life priorities with a program that provides you the exposure/volume of patients with good mentorship. You may have to weigh which of these is more important than the others.
- For Cardiology, there was more pre-interview and post-interview communication than I expected (which is a broader problem). You can and should use your mentors in the field as a resource in thinking about how to handle this.
- My subspecialty mentor was really helpful when it came time to make my final rank list (it’s easier to talk to your UCSF mentors if you ranked UCSF first, but these conversations may be more difficult if not). For a lot of people, fellowship starts to be the time that you make decisions thinking about your personal life too - taking more into account partners, kids, family, etc. Make sure you include these people in the process as well, of course.
- Post interview communication is very stressful! I don’t think there’s a great way, but people recommended letting calls go to voicemail, and then formulating a response to call back.
- Felt very awkward- received emails from 3 programs and I didn't know how to respond. It was helpful to reach out to mentors. Even if the program is not your top choice, you don't want to close any doors pre-maturely and you may work with people from this program in the future.
- Wide variety of communication--emails and even phone calls. The most frustrating were all the "bonus interviews" that would arise after interview day that I felt I could not say no to. I found the Guidebook with sample responses very helpful with how to respond to some types of communication.
- Don't read into presence or absence of communication from the program! My first-choice program was totally silent and I matched there
- Reach out to residency leadership for help with your rank list. It never hurts to talk to someone else who can serve as on objective sounding board
- **Bottom line:** You are terrific applicants and have so much to offer to fellowship programs. Your subspecialty mentors and program leadership are all here to help during the application process, so don’t hesitate to reach out. You will do great!!!
