# Supplementary material for: Development and Evaluation of a Five-Component Toolkit for Internal Medicine Residents Applying for Subspecialty Fellowships
Source: MedEdPORTAL. 2022 Mar 14;18:11228. doi: 10.15766/mep_2374-8265.11228 (PMC8918571; doi:10.15766/mep_2374-8265.11228)
Supplement: Supplementary file 1 — Elements of the Fellowship Application Toolkit.docxFellowship Application Guide.docxFellowship Application Information Night.pptxSubspecialty Breakout Room Questions.docxPreparing for Virtual Interviews.pptxMock Virtual Interview.docxSurvey Instrument.docx [file mep_2374-8265.11228-s001.zip › C. Fellowship Application Information Night.pptx]

## Slide 1
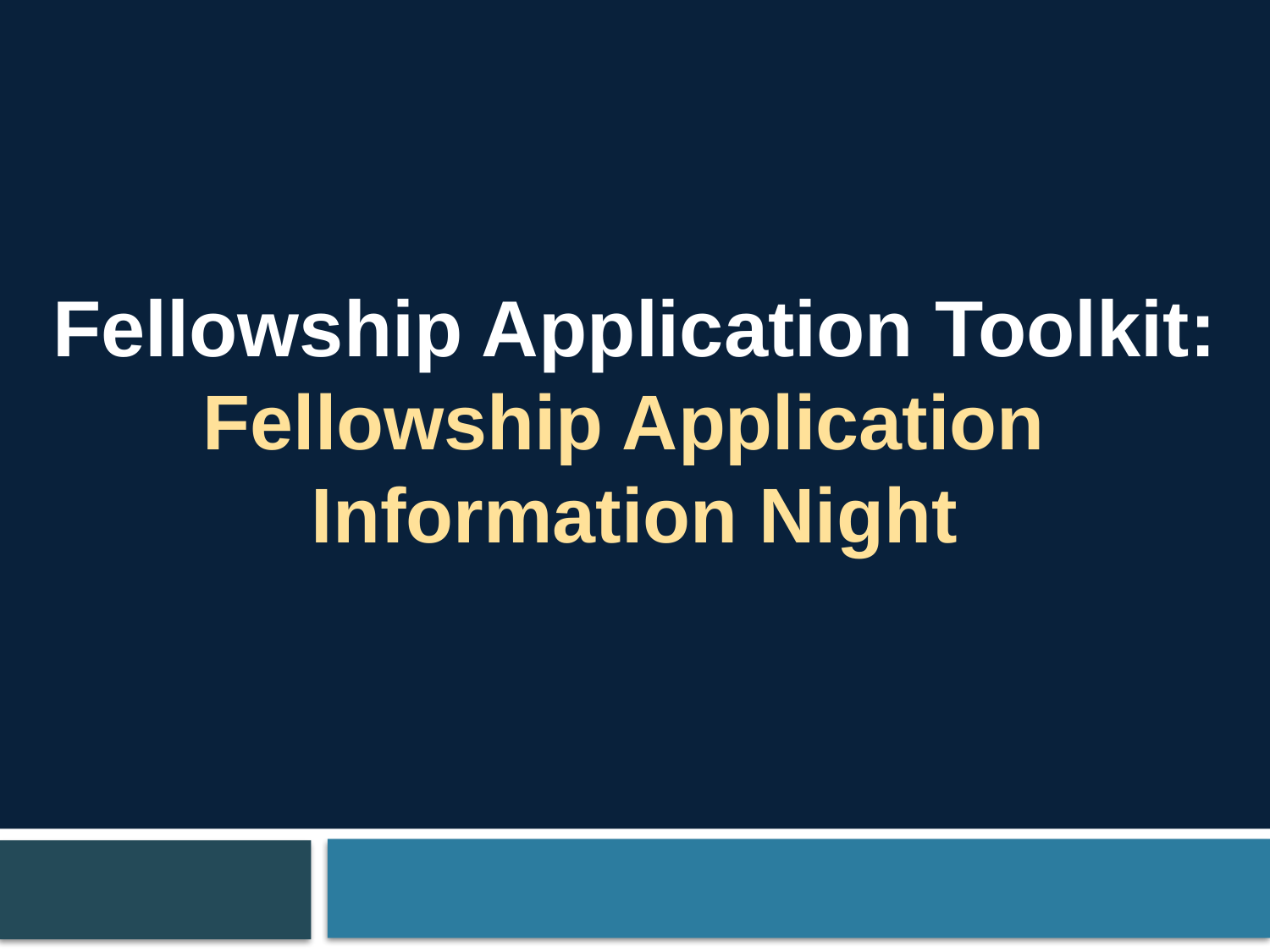

Fellowship Application Toolkit:
Fellowship Application
Information Night

## Slide 2
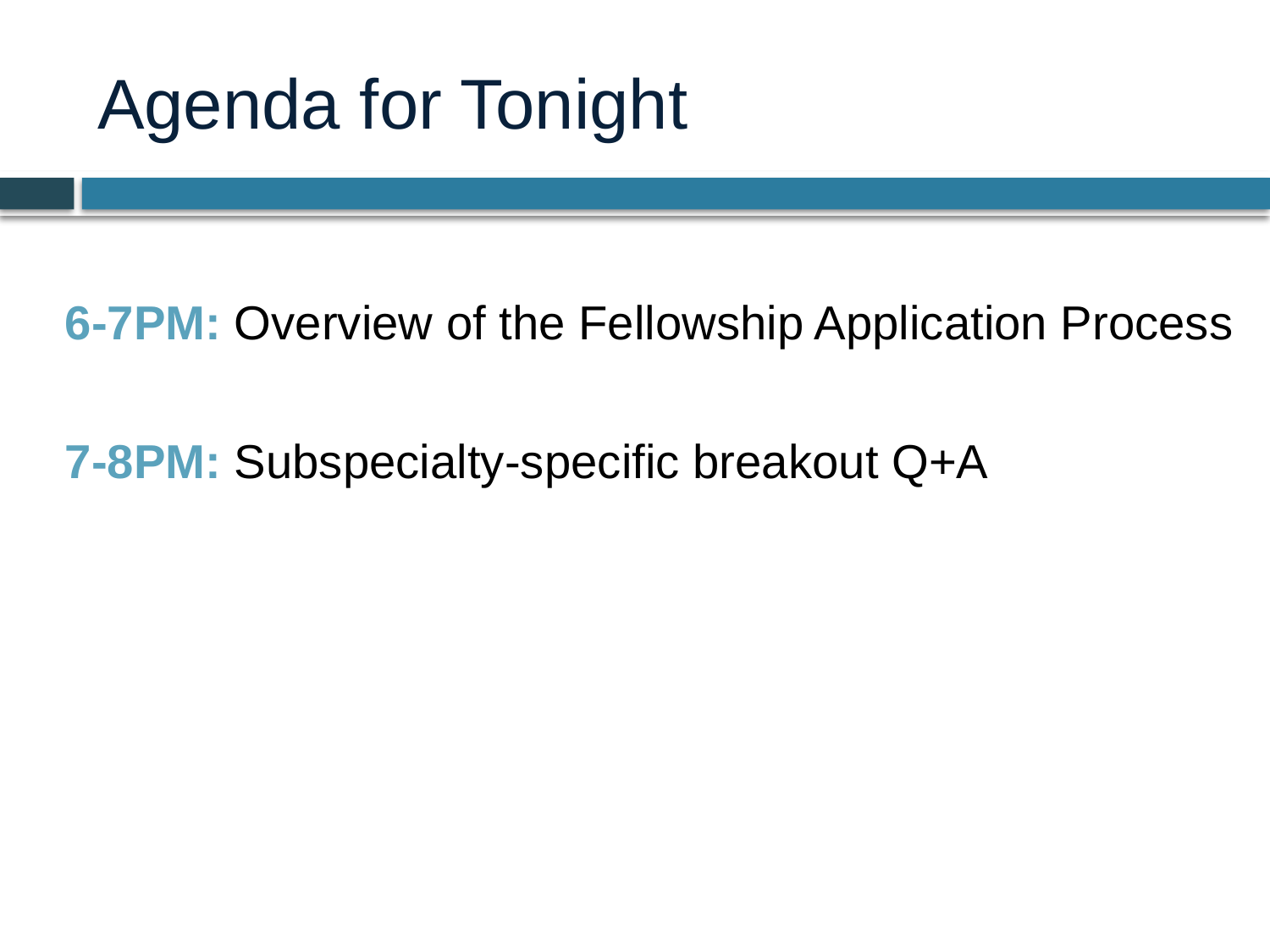

# Agenda for Tonight
6-7PM: Overview of the Fellowship Application Process
7-8PM: Subspecialty-specific breakout Q+A

## Slide 3
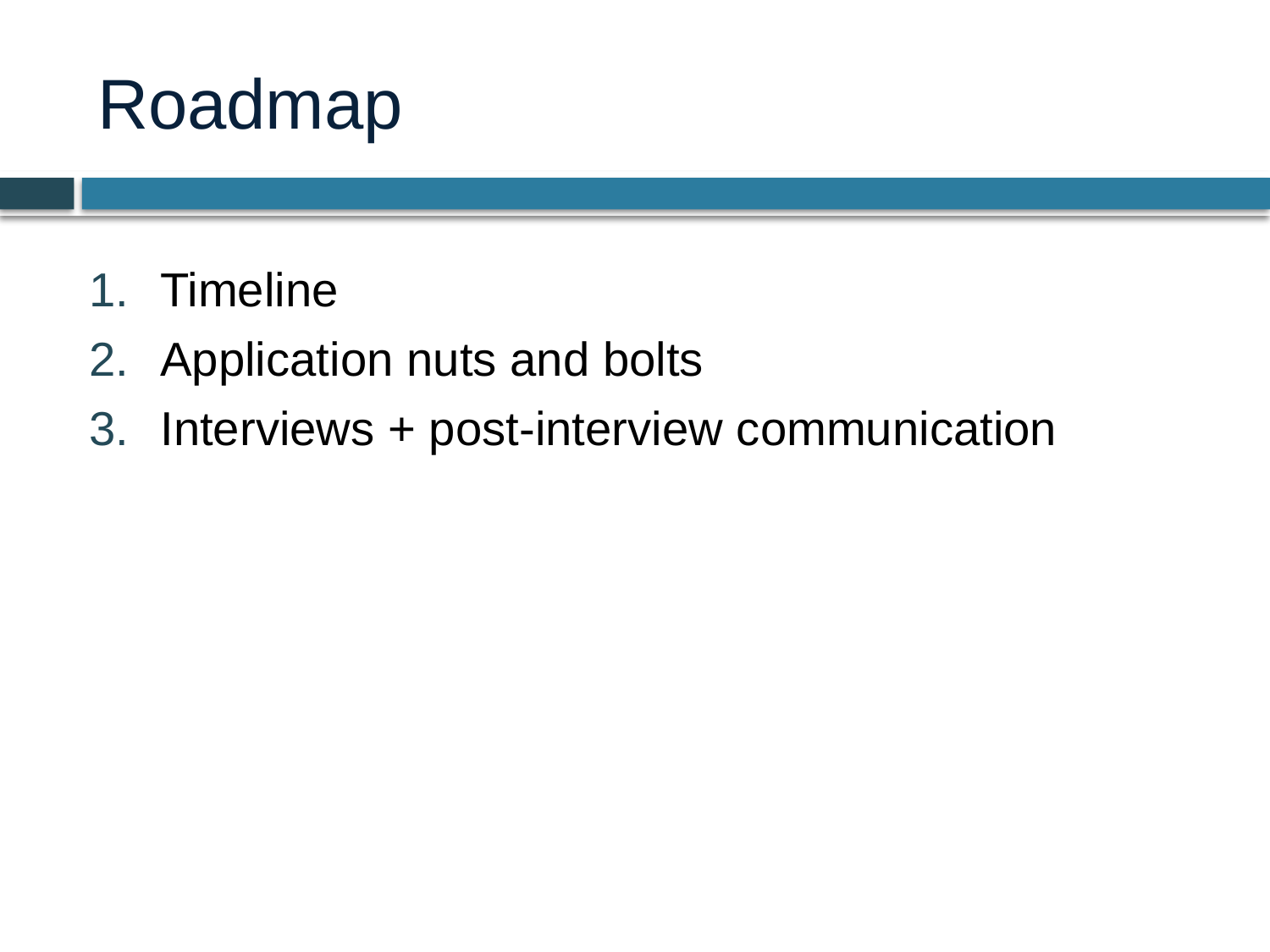

# Roadmap
Timeline
Application nuts and bolts
Interviews + post-interview communication

## Slide 4
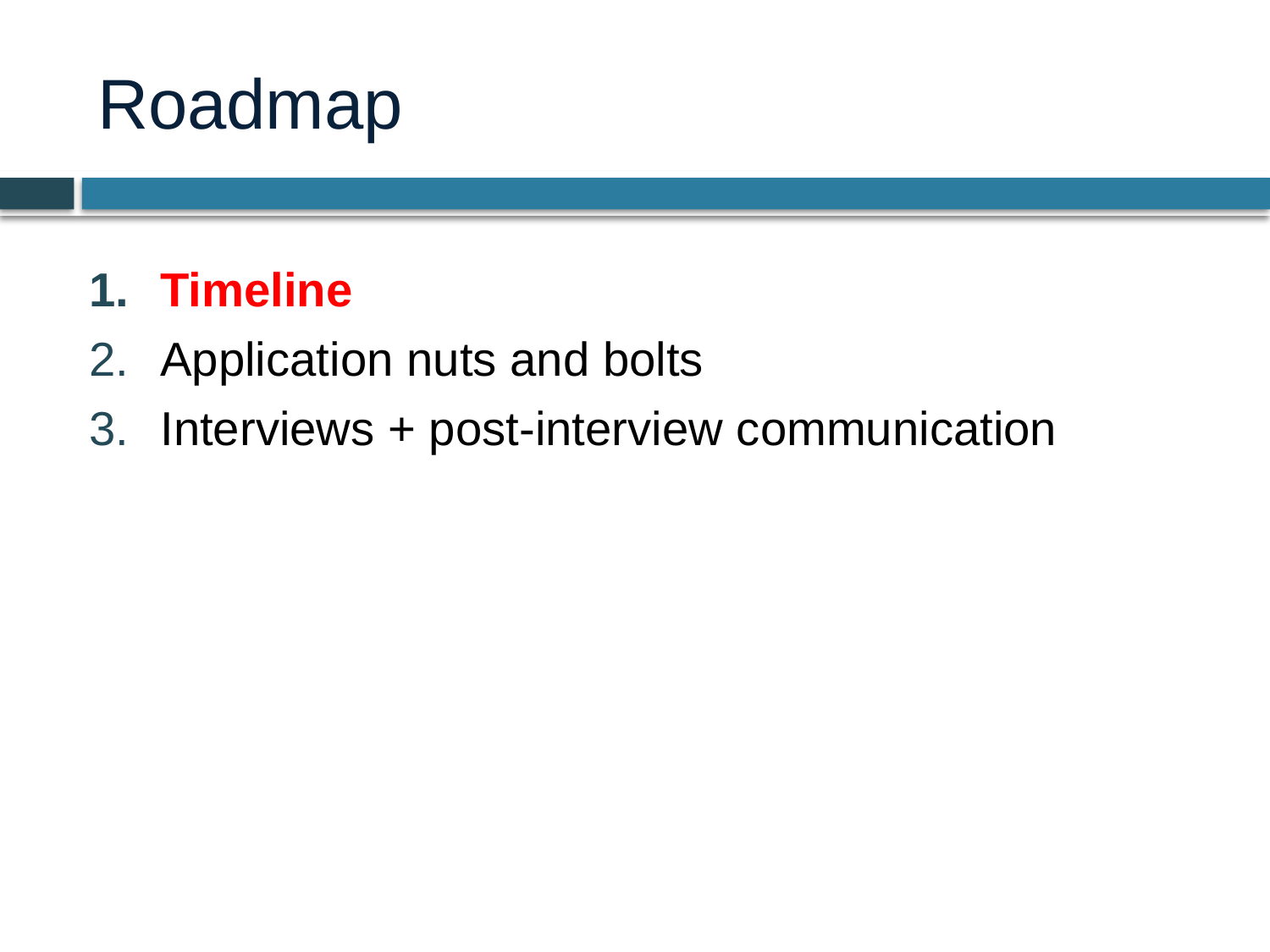

# Roadmap
Timeline
Application nuts and bolts
Interviews + post-interview communication

## Slide 5
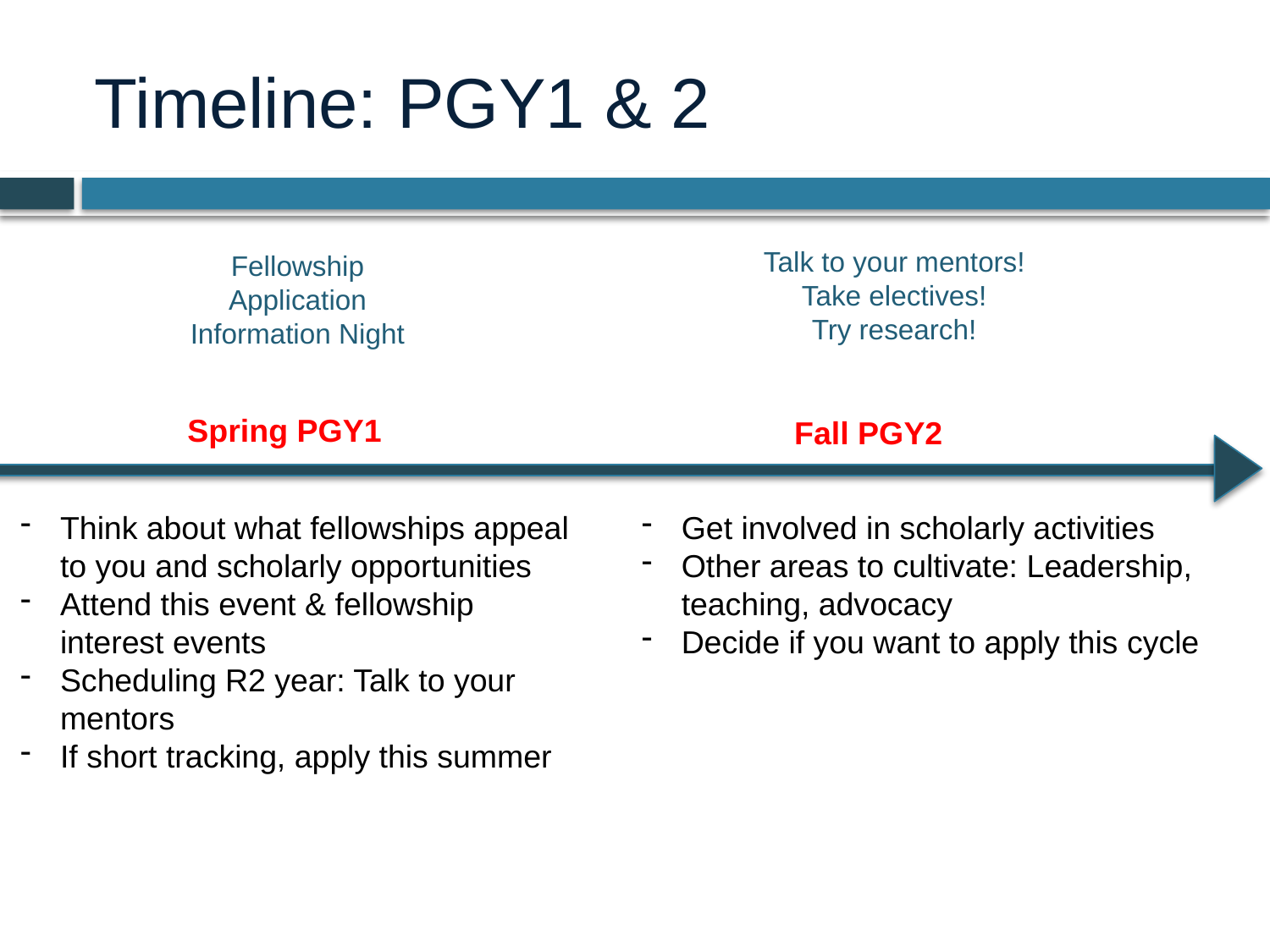

Timeline: PGY1 & 2
Talk to your mentors!
Take electives!
Try research!
Fellowship Application Information Night
Spring PGY1
Fall PGY2
Think about what fellowships appeal to you and scholarly opportunities
Attend this event & fellowship interest events
Scheduling R2 year: Talk to your mentors
If short tracking, apply this summer
Get involved in scholarly activities
Other areas to cultivate: Leadership, teaching, advocacy
Decide if you want to apply this cycle

## Slide 6
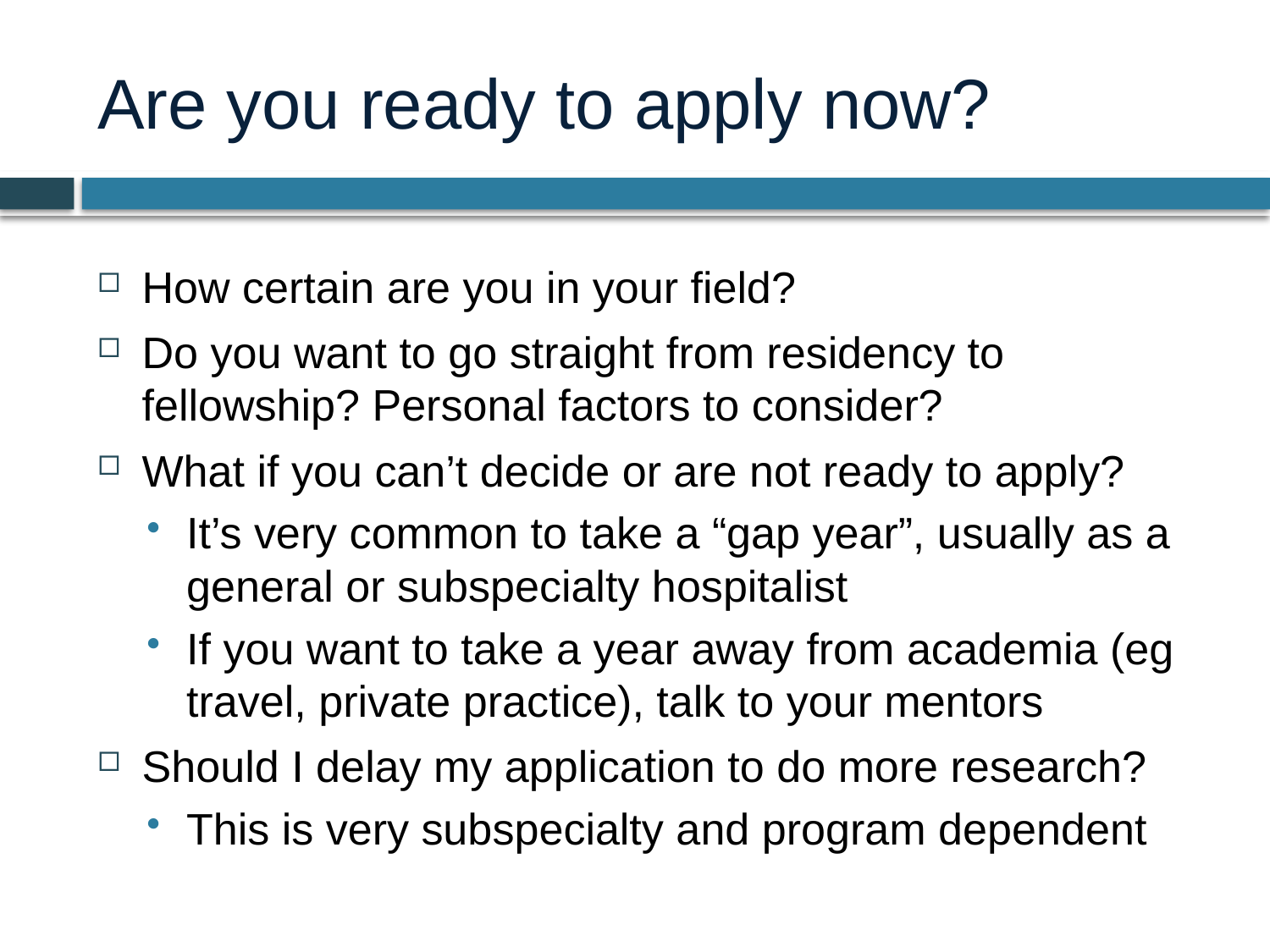

# Are you ready to apply now?
How certain are you in your field?
Do you want to go straight from residency to fellowship? Personal factors to consider?
What if you can’t decide or are not ready to apply?
It’s very common to take a “gap year”, usually as a general or subspecialty hospitalist
If you want to take a year away from academia (eg travel, private practice), talk to your mentors
Should I delay my application to do more research?
This is very subspecialty and program dependent

## Slide 7
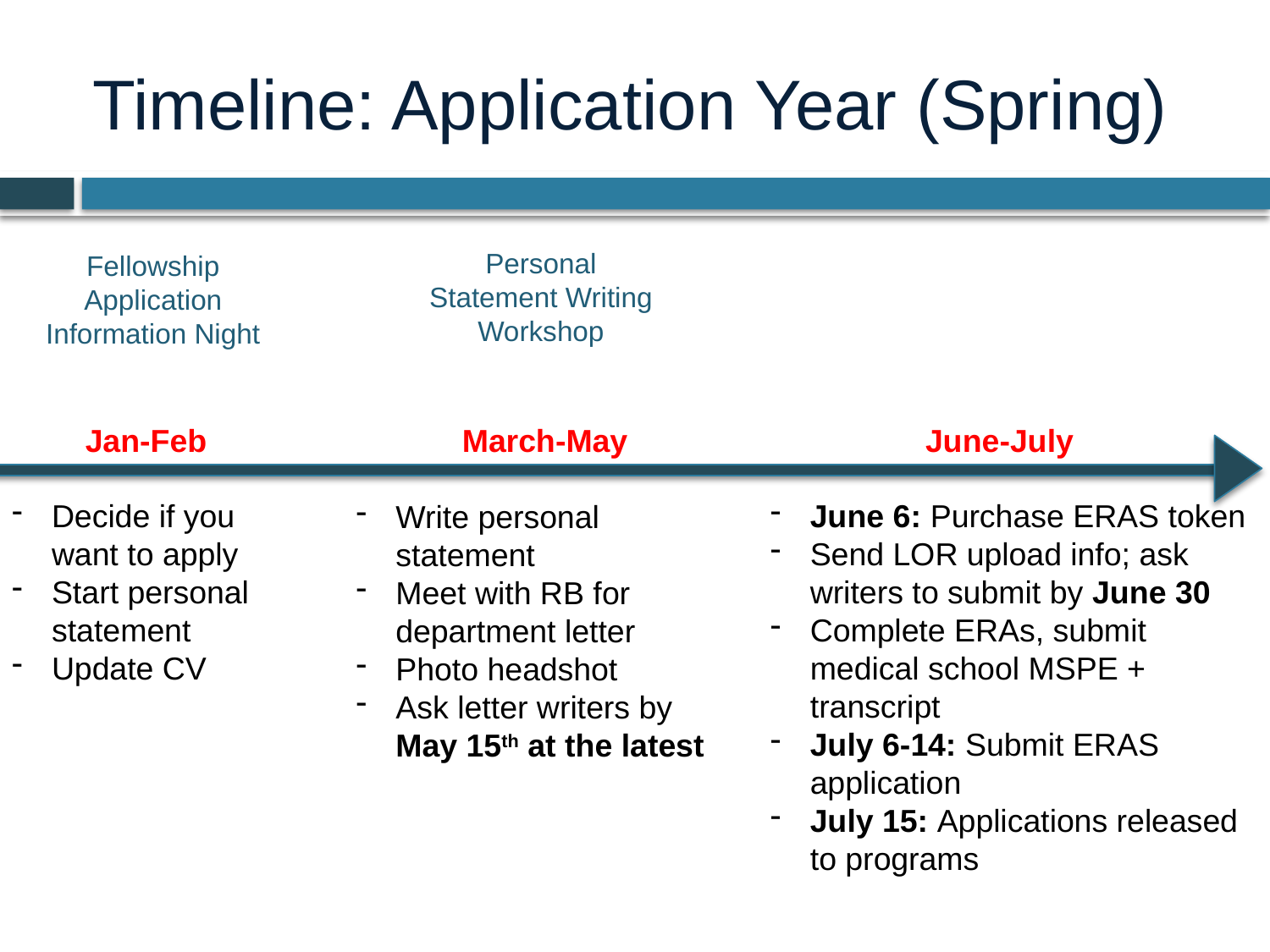

Timeline: Application Year (Spring)
Personal Statement Writing Workshop
Fellowship Application Information Night
Jan-Feb
March-May
June-July
Decide if you want to apply
Start personal statement
Update CV
June 6: Purchase ERAS token
Send LOR upload info; ask writers to submit by June 30
Complete ERAs, submit medical school MSPE + transcript
July 6-14: Submit ERAS application
July 15: Applications released to programs
Write personal statement
Meet with RB for department letter
Photo headshot
Ask letter writers by May 15th at the latest

## Slide 8
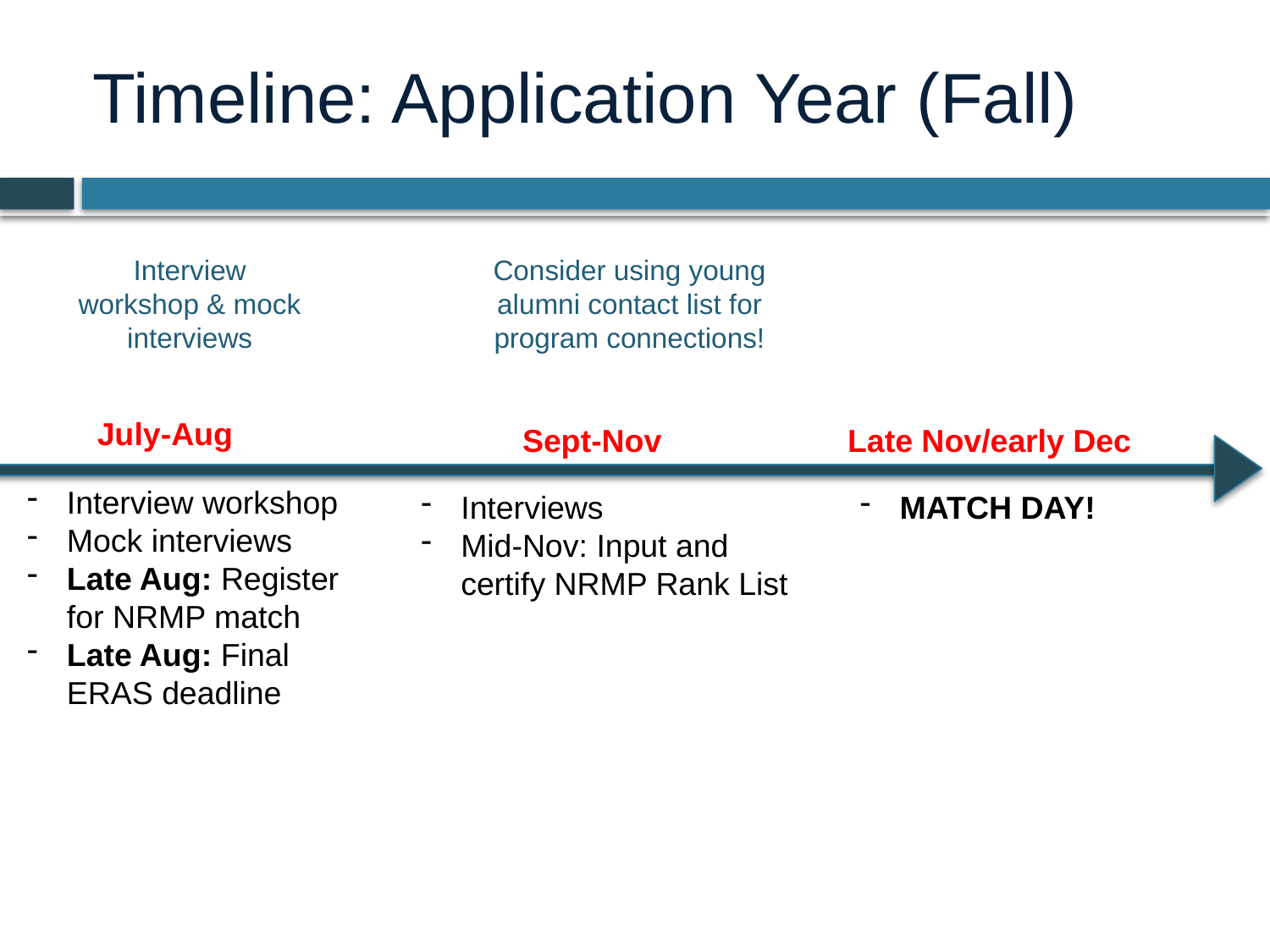

Timeline: Application Year (Fall)
Interview workshop & mock interviews
Consider using young alumni contact list for program connections!
July-Aug
Sept-Nov
Late Nov/early Dec
Interview workshop
Mock interviews
Late Aug: Register for NRMP match
Late Aug: Final ERAS deadline
Interviews
Mid-Nov: Input and certify NRMP Rank List
MATCH DAY!

## Slide 9
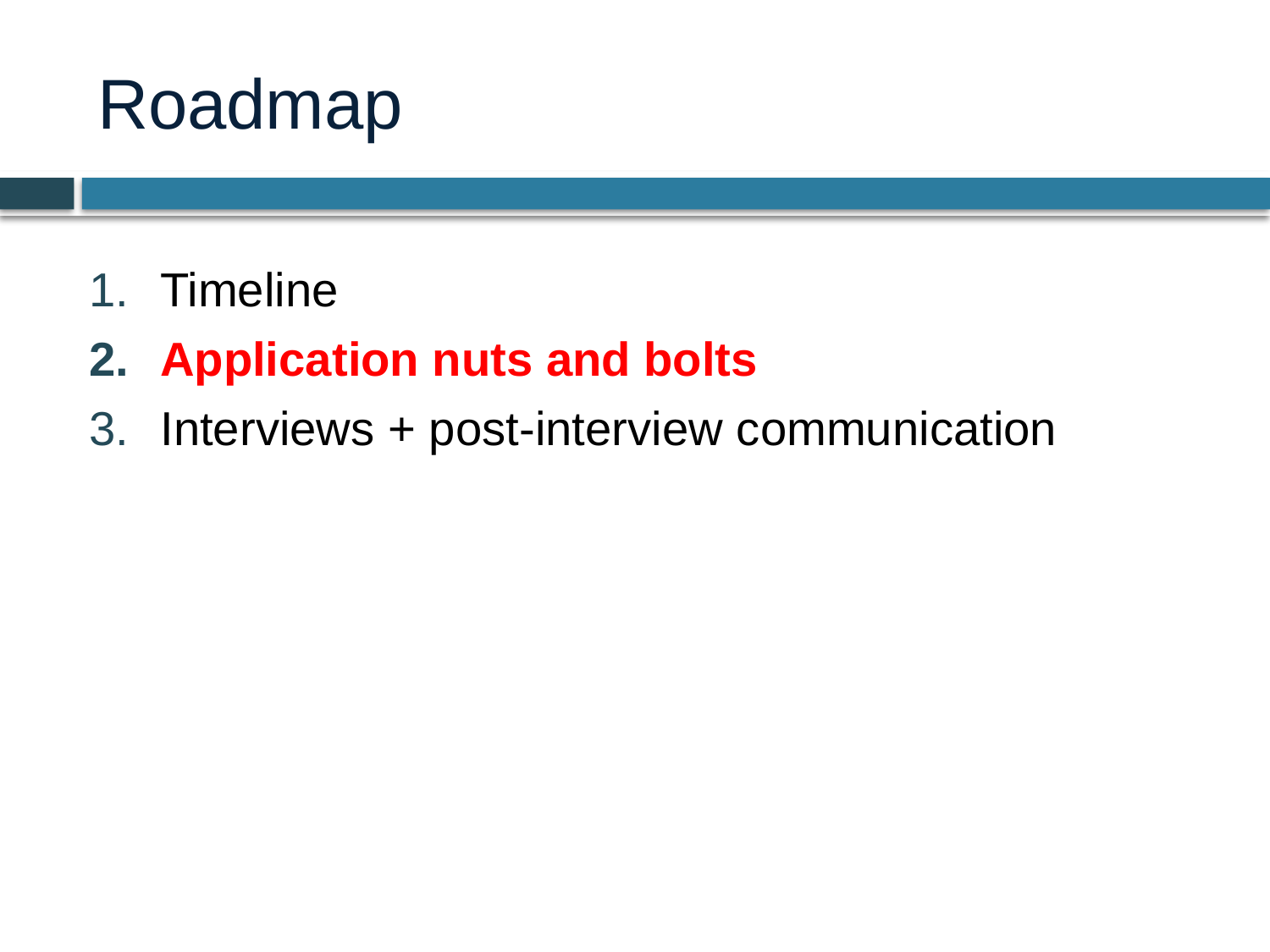

# Roadmap
Timeline
Application nuts and bolts
Interviews + post-interview communication

## Slide 10
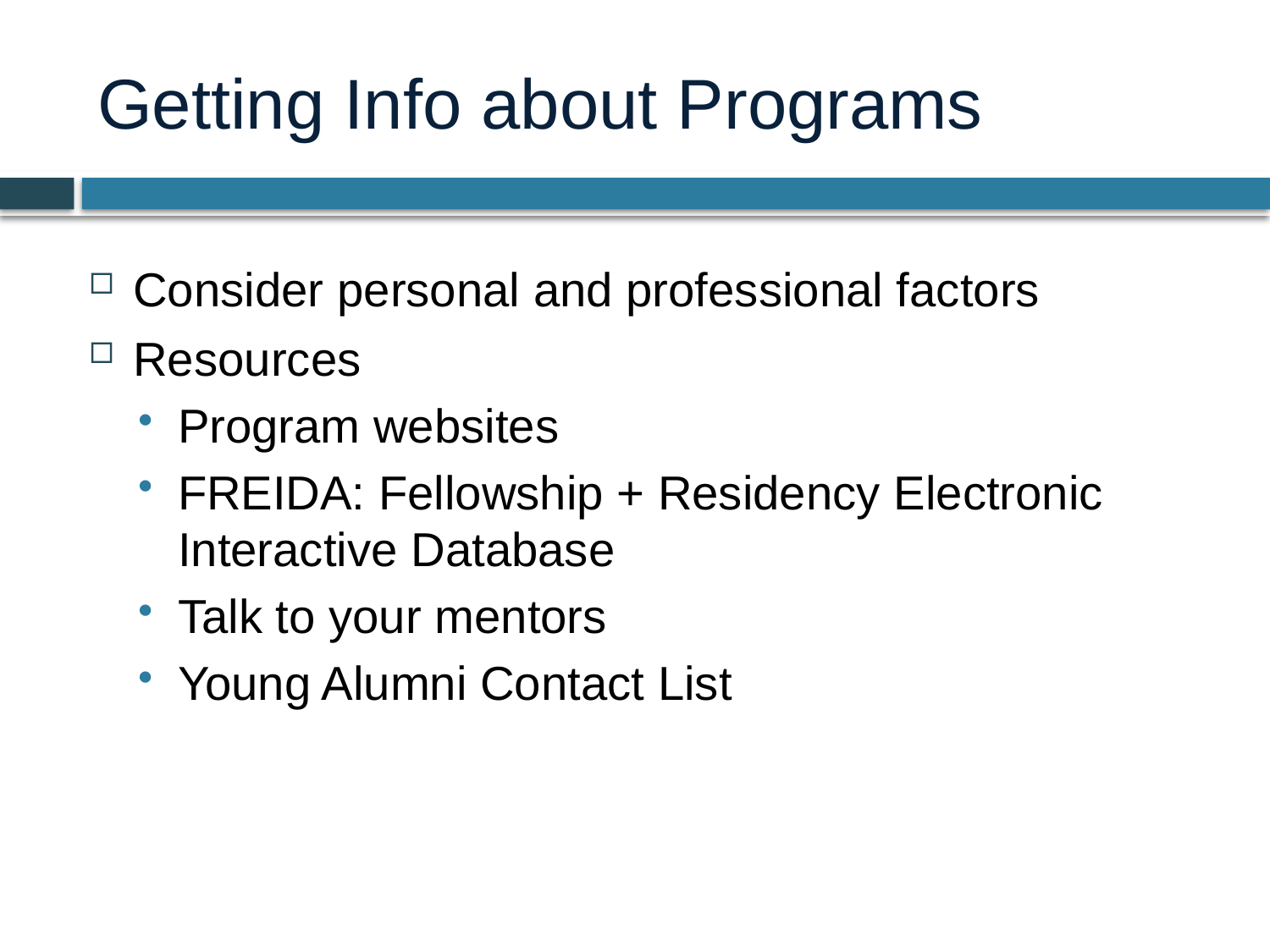

# Getting Info about Programs
Consider personal and professional factors
Resources
Program websites
FREIDA: Fellowship + Residency Electronic Interactive Database
Talk to your mentors
Young Alumni Contact List

## Slide 11
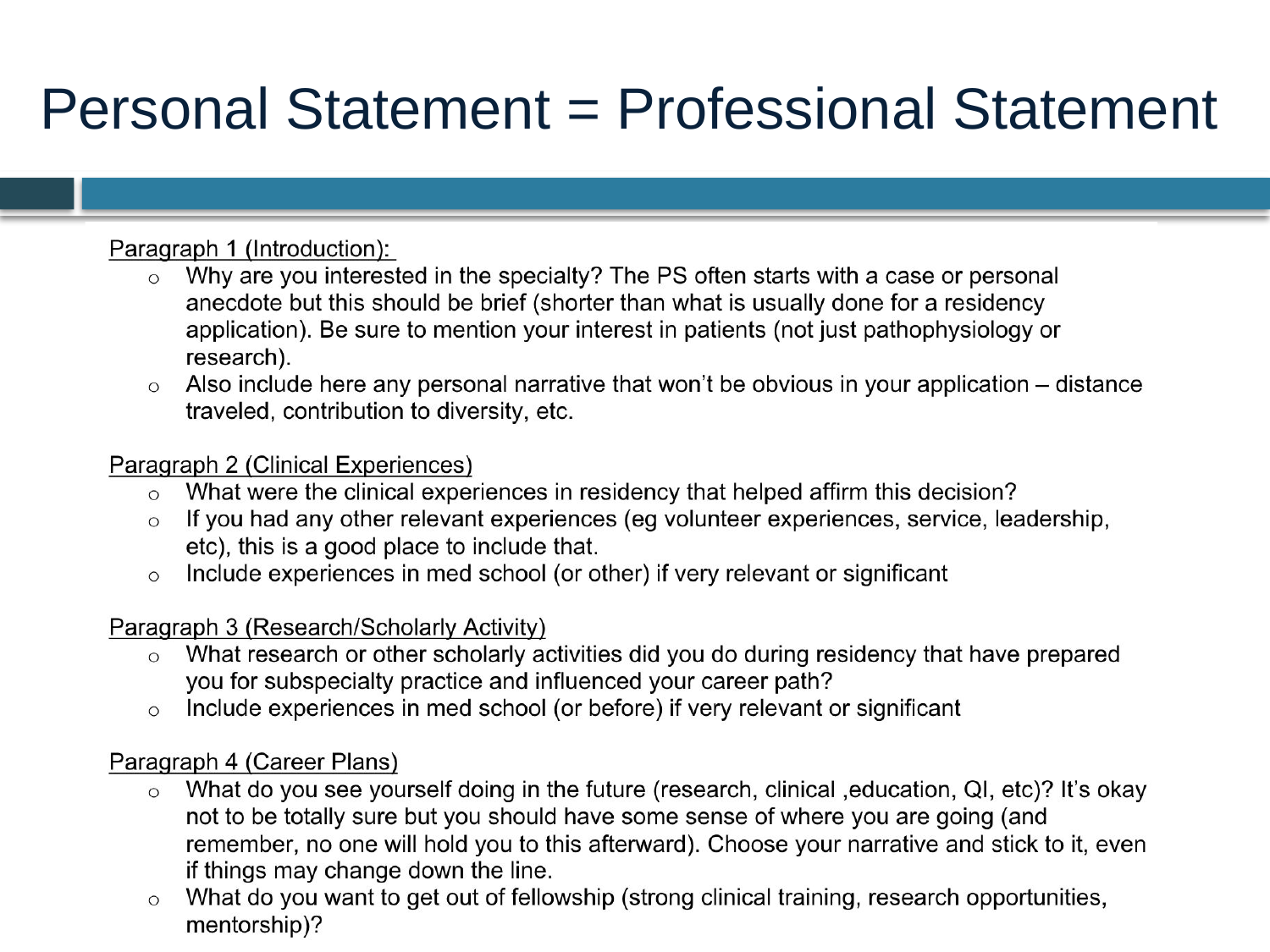

# Personal Statement = Professional Statement

## Slide 12
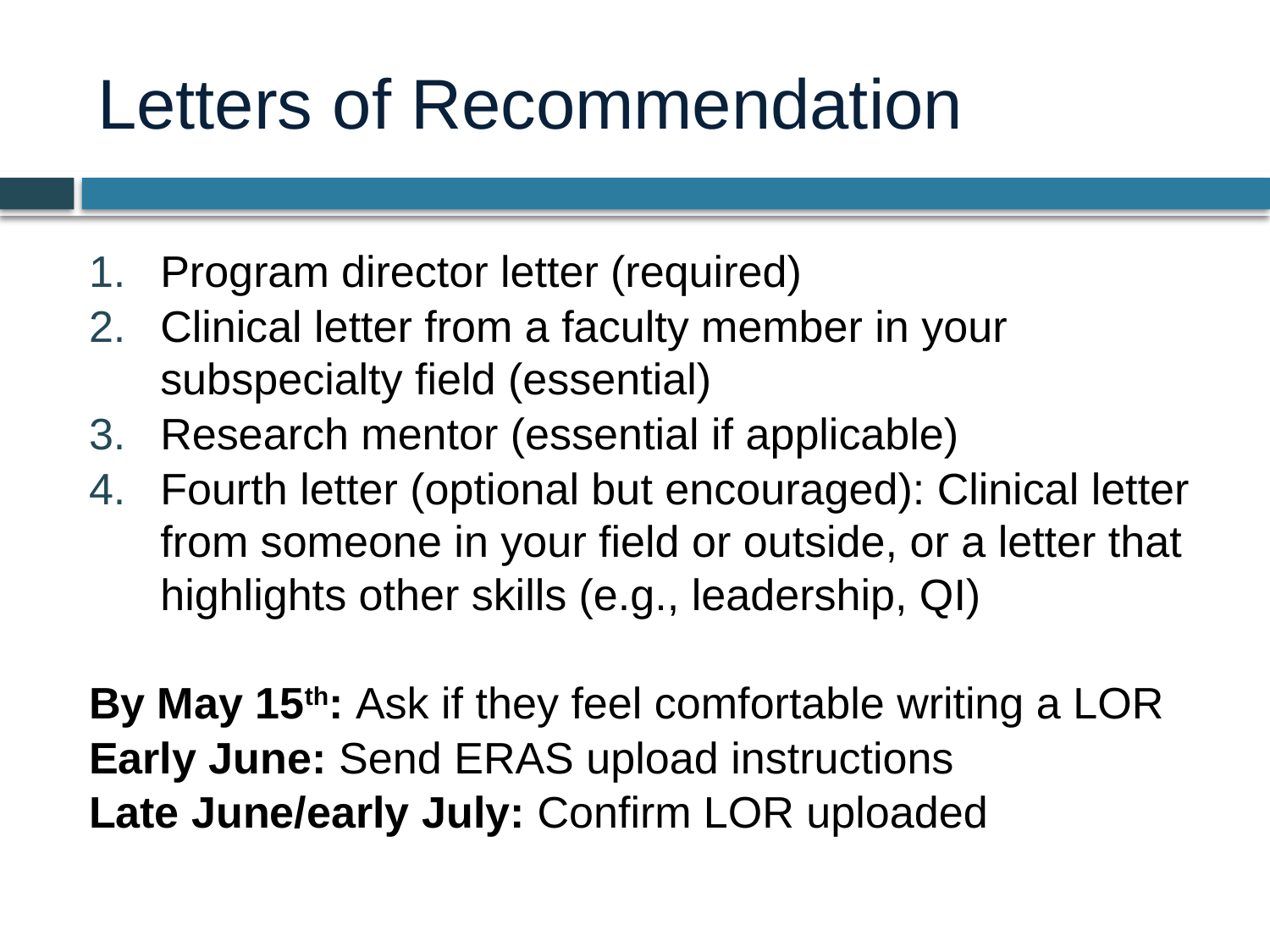

# Letters of Recommendation
Program director letter (required)
Clinical letter from a faculty member in your subspecialty field (essential)
Research mentor (essential if applicable)
Fourth letter (optional but encouraged): Clinical letter from someone in your field or outside, or a letter that highlights other skills (e.g., leadership, QI)
By May 15th: Ask if they feel comfortable writing a LOR
Early June: Send ERAS upload instructions
Late June/early July: Confirm LOR uploaded

## Slide 13
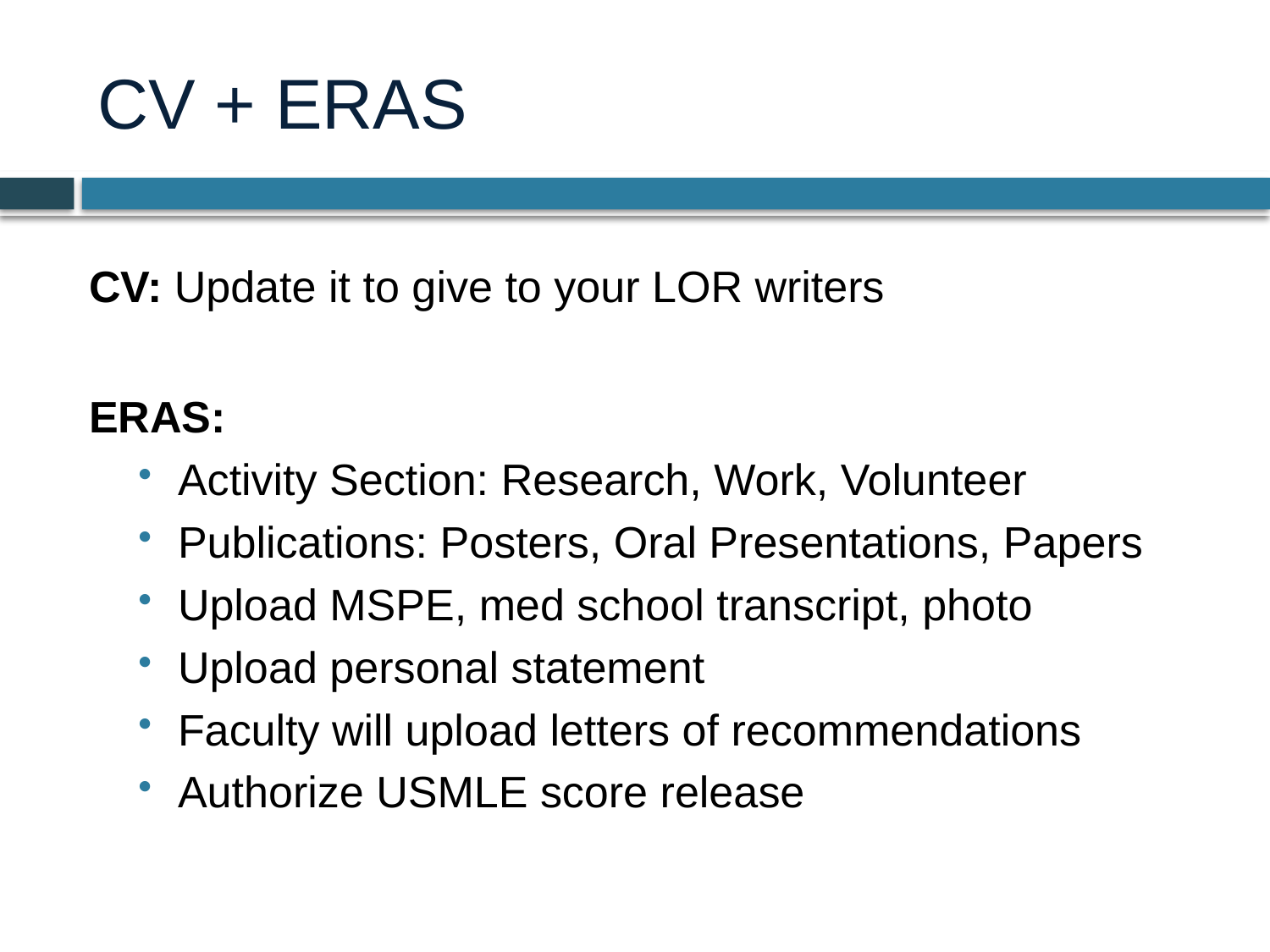

# CV + ERAS
CV: Update it to give to your LOR writers
ERAS:
Activity Section: Research, Work, Volunteer
Publications: Posters, Oral Presentations, Papers
Upload MSPE, med school transcript, photo
Upload personal statement
Faculty will upload letters of recommendations
Authorize USMLE score release

## Slide 14
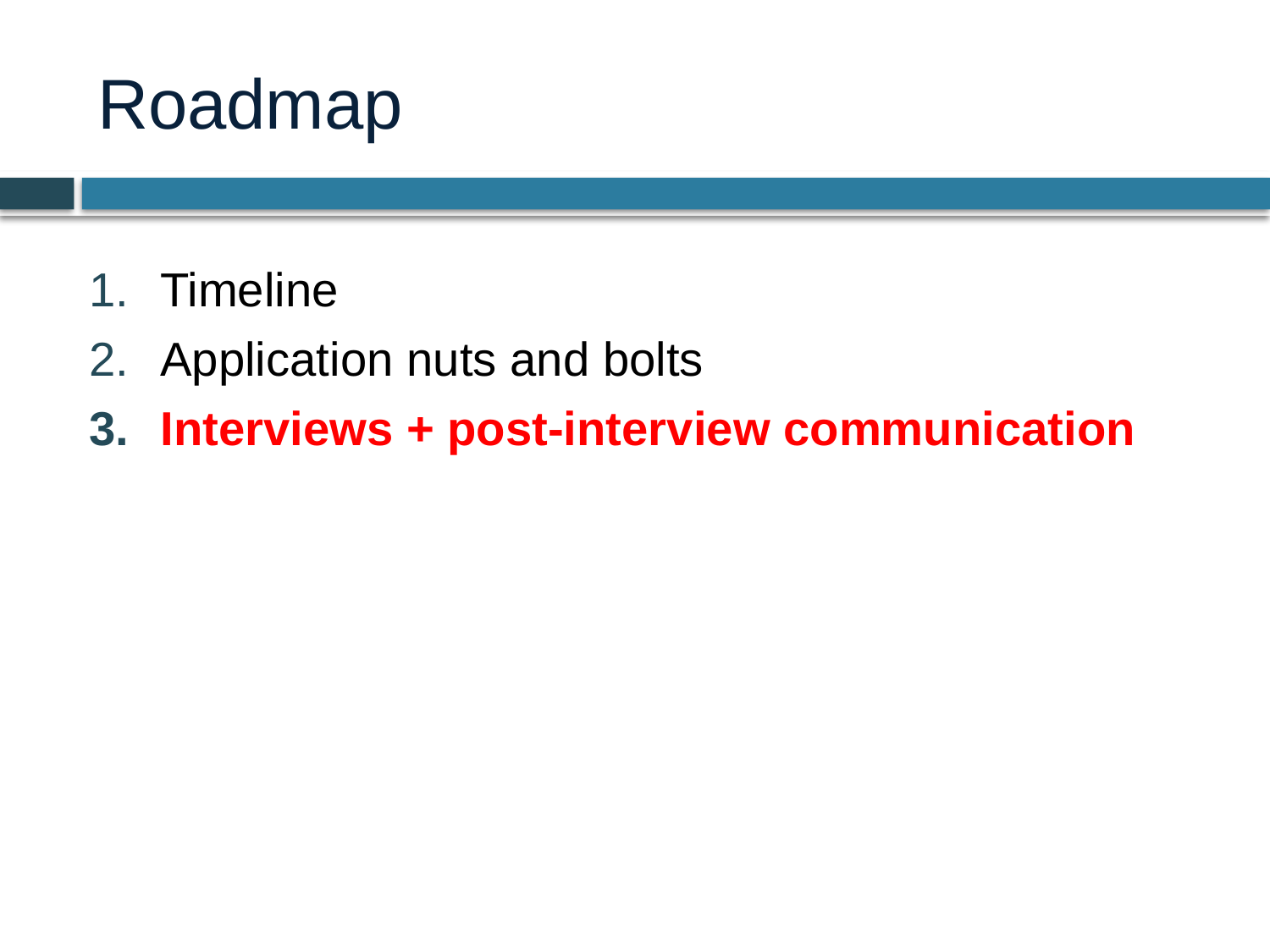

# Roadmap
Timeline
Application nuts and bolts
Interviews + post-interview communication

## Slide 15
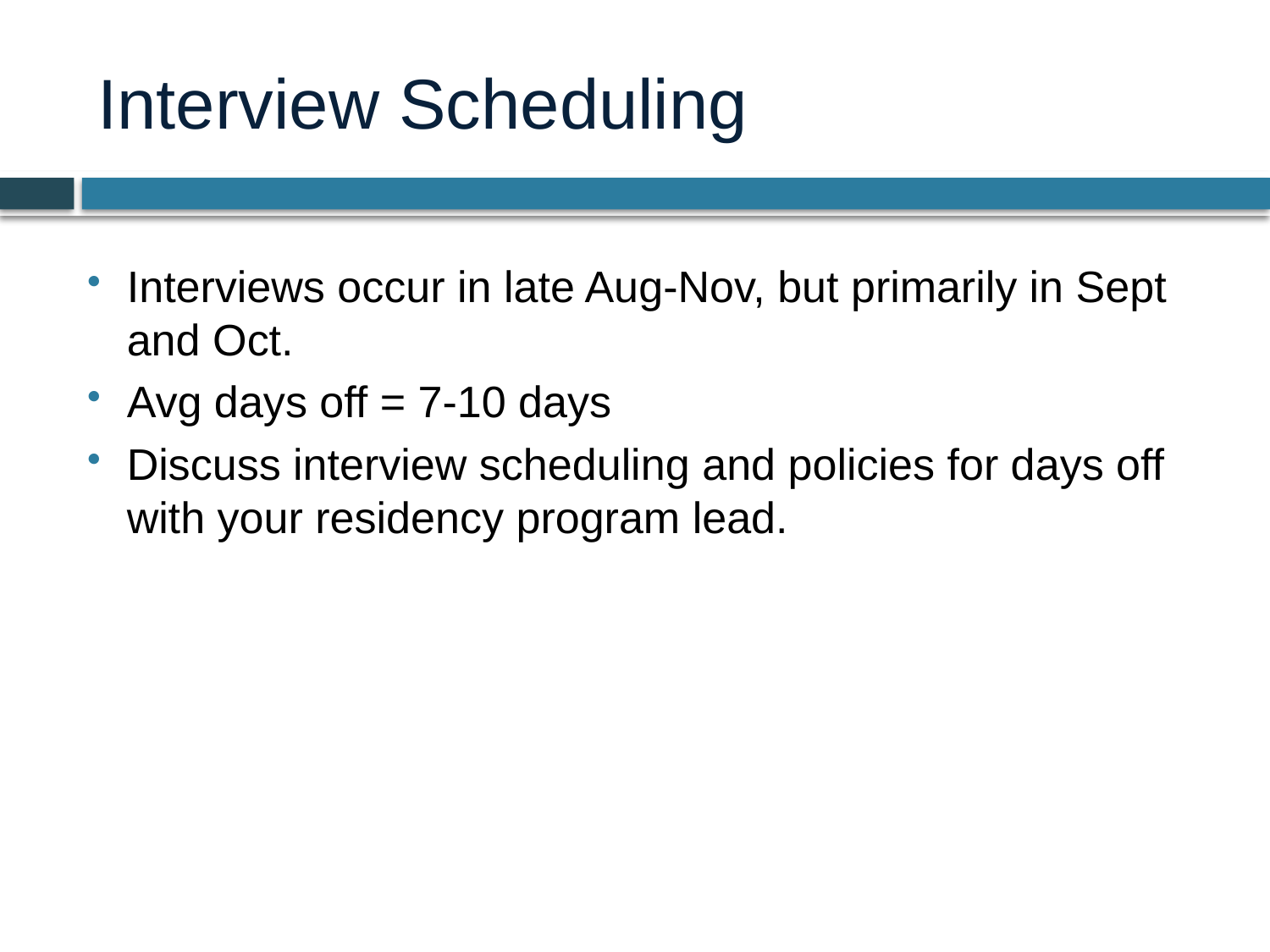

# Interview Scheduling
Interviews occur in late Aug-Nov, but primarily in Sept and Oct.
Avg days off = 7-10 days
Discuss interview scheduling and policies for days off with your residency program lead.

## Slide 16
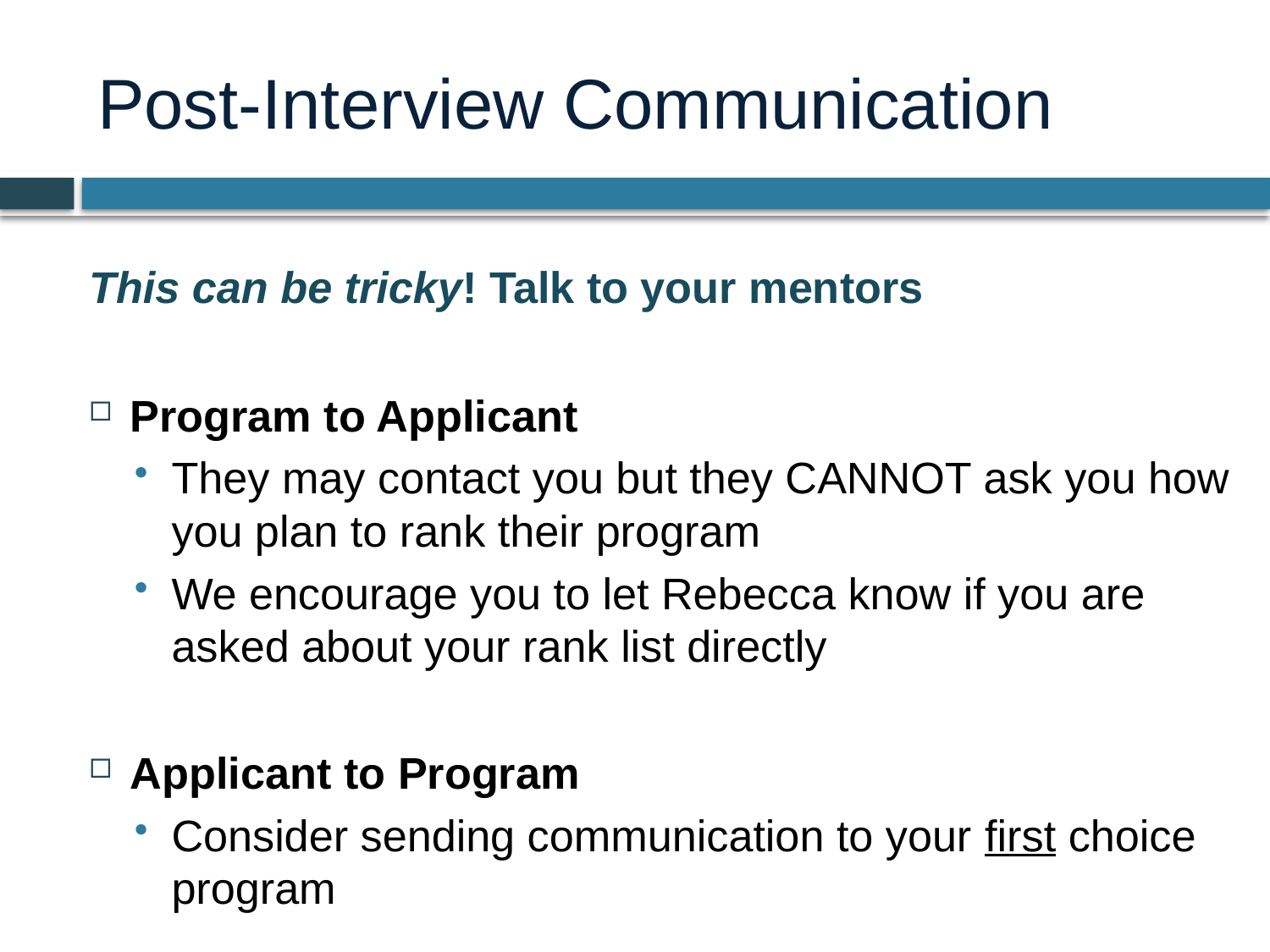

# Post-Interview Communication
This can be tricky! Talk to your mentors
Program to Applicant
They may contact you but they CANNOT ask you how you plan to rank their program
We encourage you to let Rebecca know if you are asked about your rank list directly
Applicant to Program
Consider sending communication to your first choice program

## Slide 17
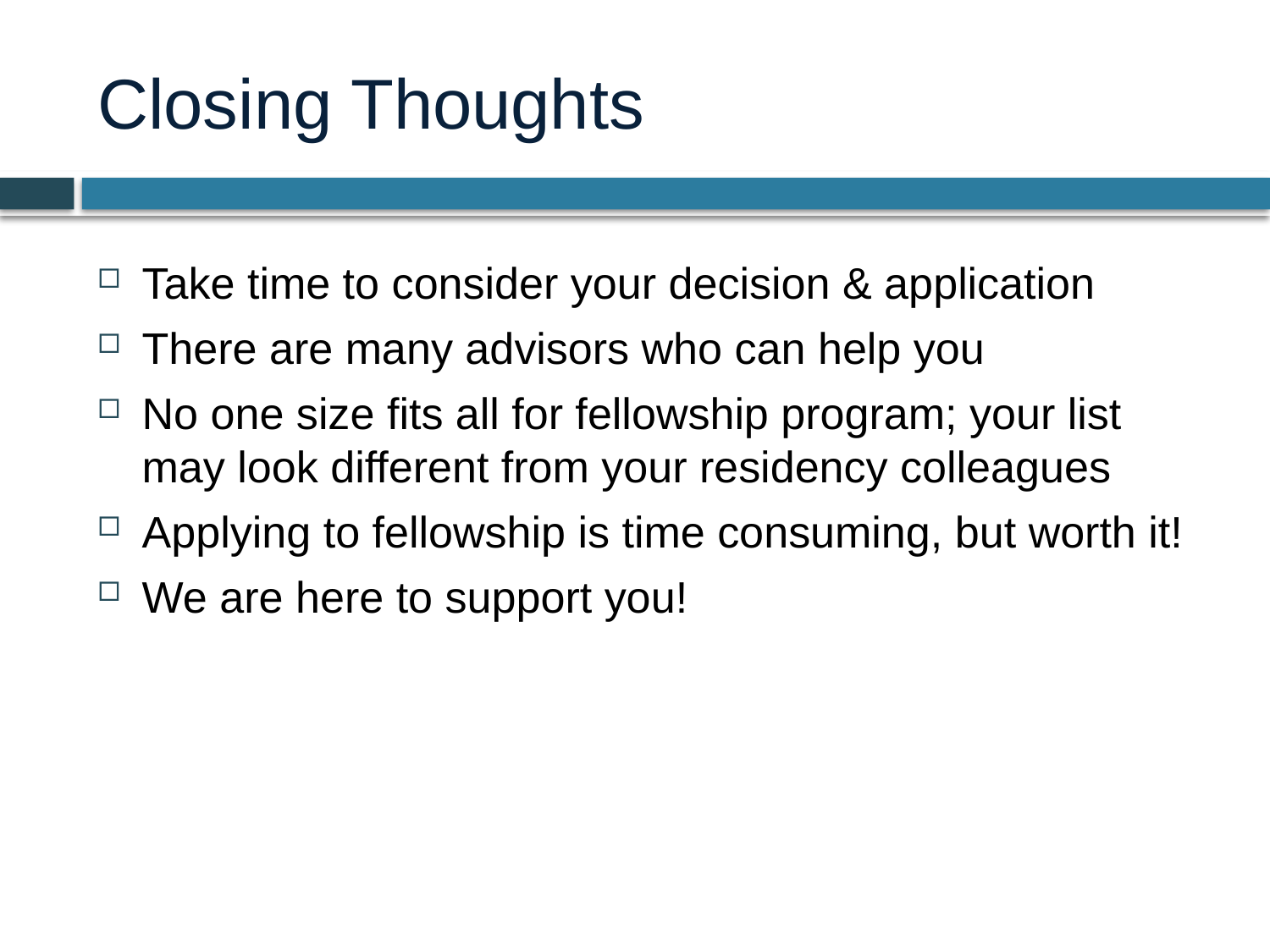

# Closing Thoughts
Take time to consider your decision & application
There are many advisors who can help you
No one size fits all for fellowship program; your list may look different from your residency colleagues
Applying to fellowship is time consuming, but worth it!
We are here to support you!

## Slide 18
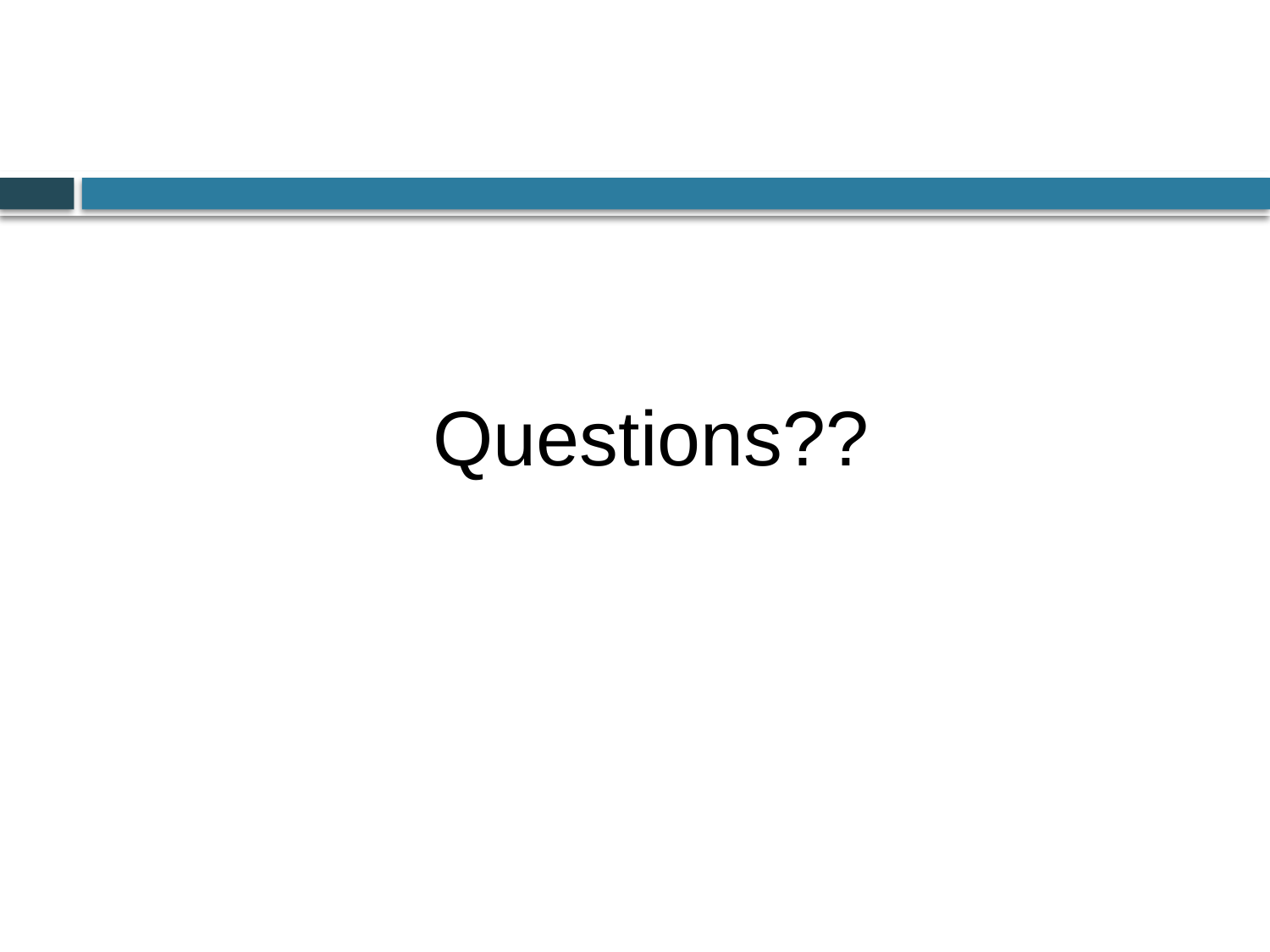

Questions??

## Slide 19
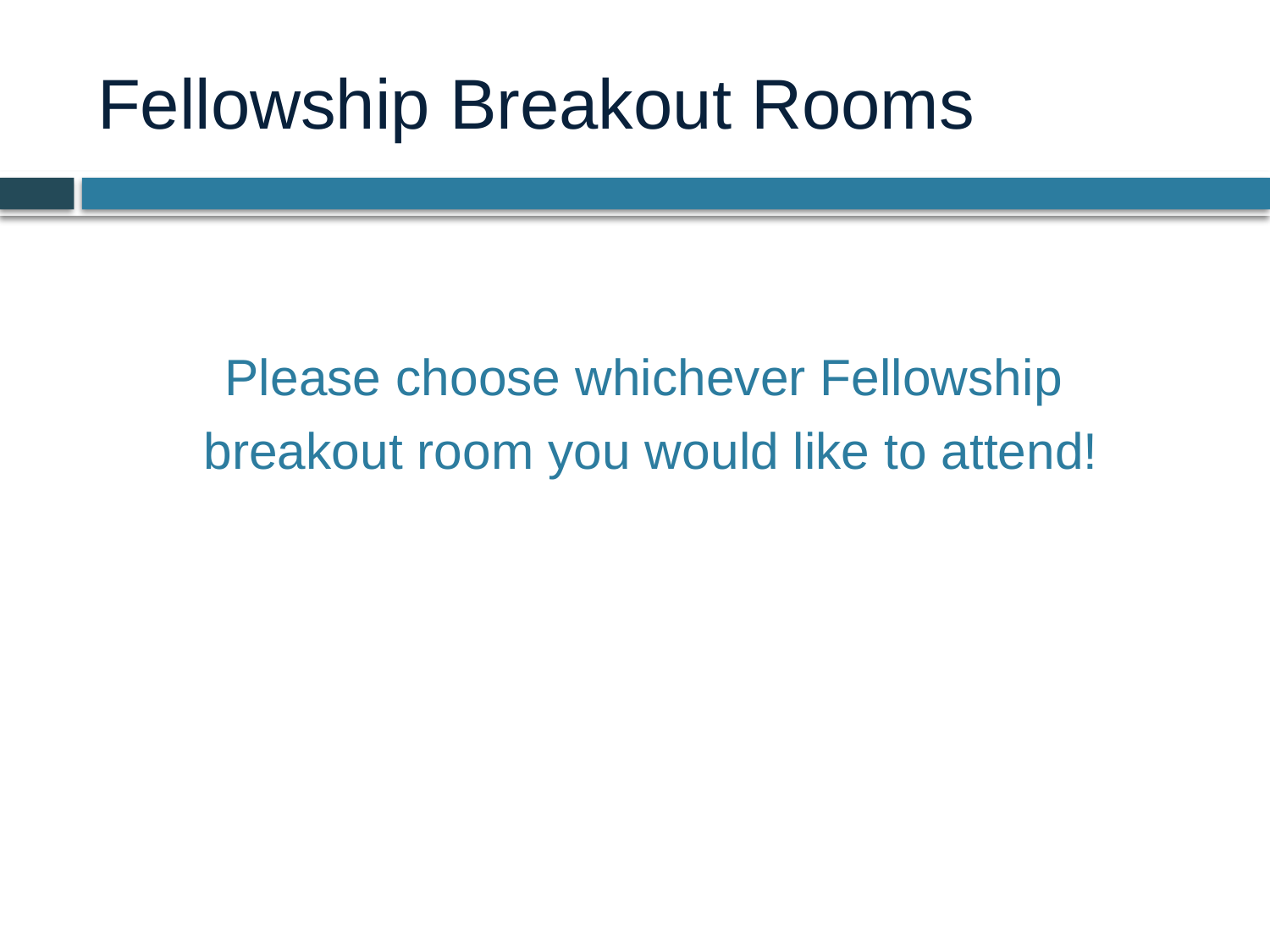

# Fellowship Breakout Rooms
Please choose whichever Fellowship
breakout room you would like to attend!
