# Supplementary material for: Development and Evaluation of a Five-Component Toolkit for Internal Medicine Residents Applying for Subspecialty Fellowships
Source: MedEdPORTAL. 2022 Mar 14;18:11228. doi: 10.15766/mep_2374-8265.11228 (PMC8918571; doi:10.15766/mep_2374-8265.11228)
Supplement: Supplementary file 1 — Elements of the Fellowship Application Toolkit.docxFellowship Application Guide.docxFellowship Application Information Night.pptxSubspecialty Breakout Room Questions.docxPreparing for Virtual Interviews.pptxMock Virtual Interview.docxSurvey Instrument.docx [file mep_2374-8265.11228-s001.zip › E. Preparing for Virtual Interviews.pptx]

## Slide 1
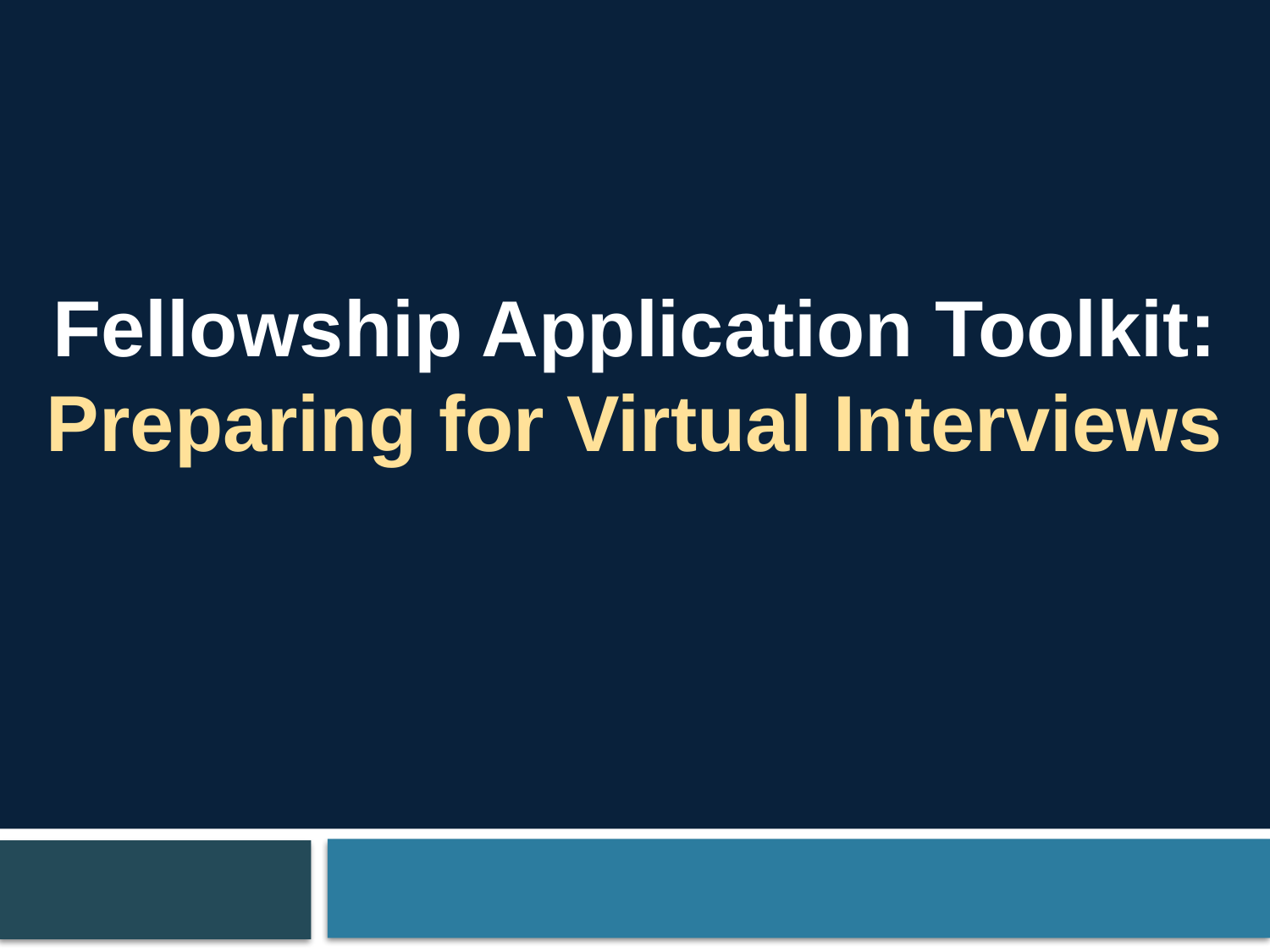

Fellowship Application Toolkit:
Preparing for Virtual Interviews

## Slide 2
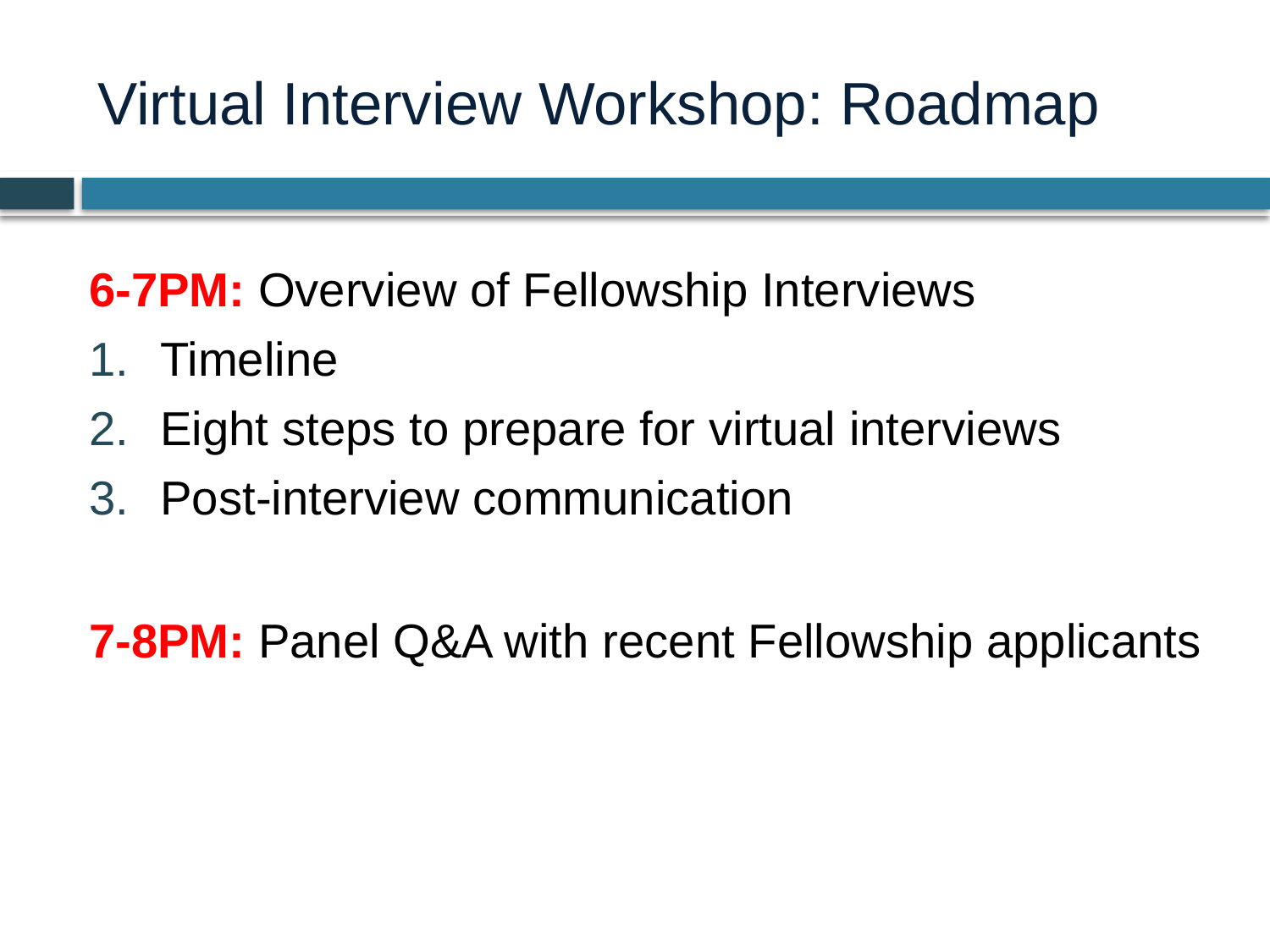

# Virtual Interview Workshop: Roadmap
6-7PM: Overview of Fellowship Interviews
Timeline
Eight steps to prepare for virtual interviews
Post-interview communication
7-8PM: Panel Q&A with recent Fellowship applicants

## Slide 3
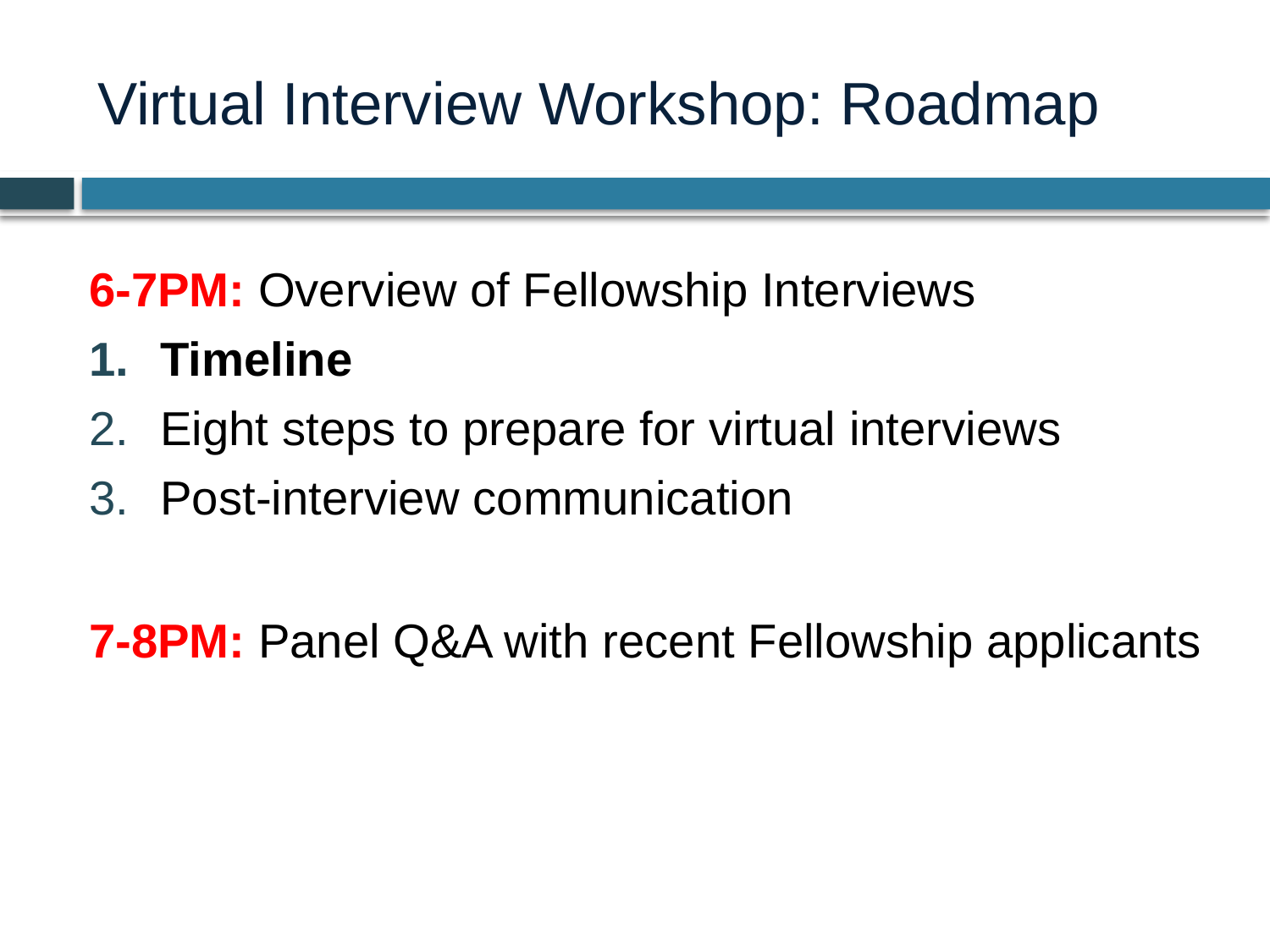

# Virtual Interview Workshop: Roadmap
6-7PM: Overview of Fellowship Interviews
Timeline
Eight steps to prepare for virtual interviews
Post-interview communication
7-8PM: Panel Q&A with recent Fellowship applicants

## Slide 4
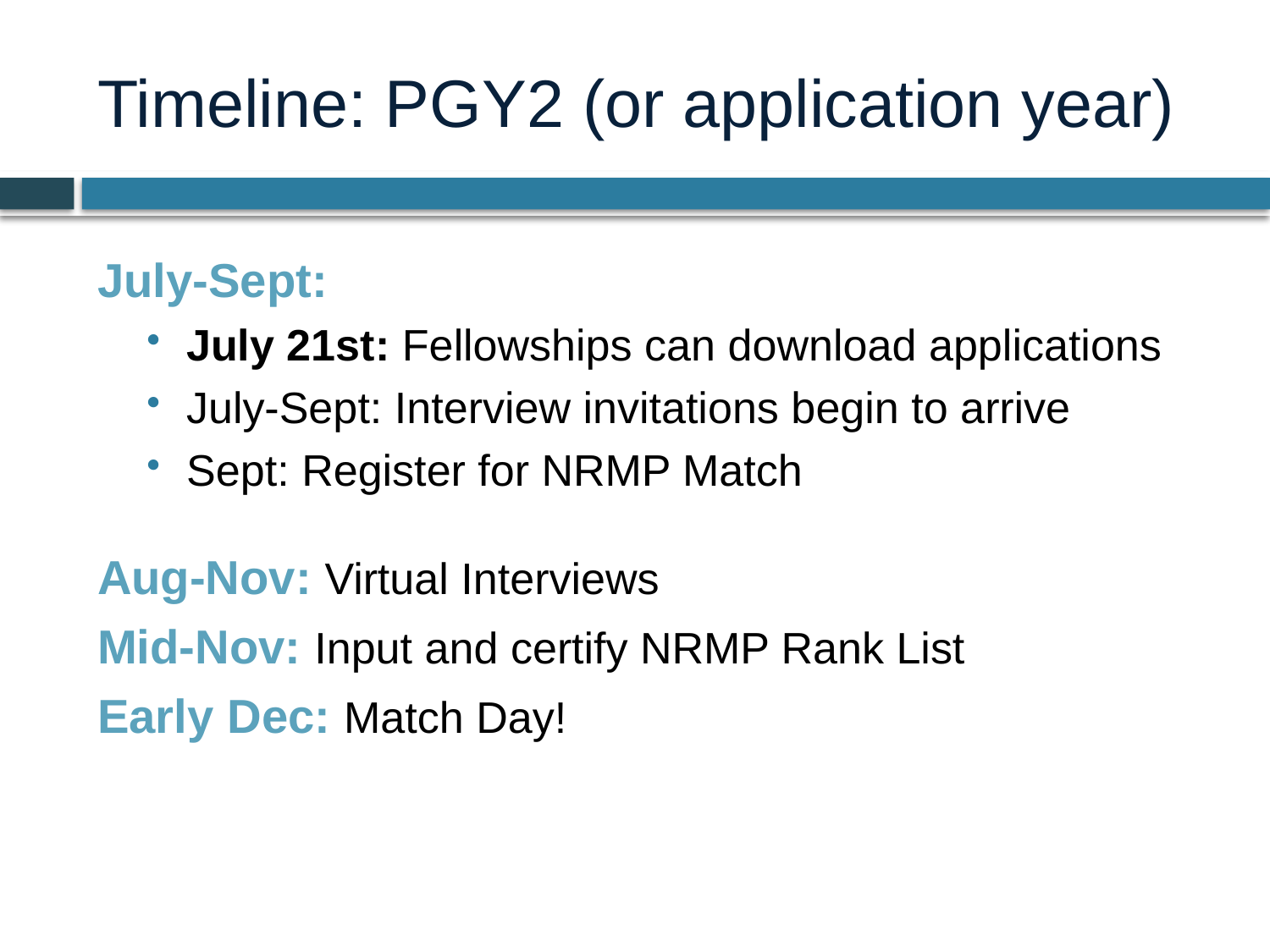

# Timeline: PGY2 (or application year)
July-Sept:
July 21st: Fellowships can download applications
July-Sept: Interview invitations begin to arrive
Sept: Register for NRMP Match
Aug-Nov: Virtual Interviews
Mid-Nov: Input and certify NRMP Rank List
Early Dec: Match Day!

## Slide 5
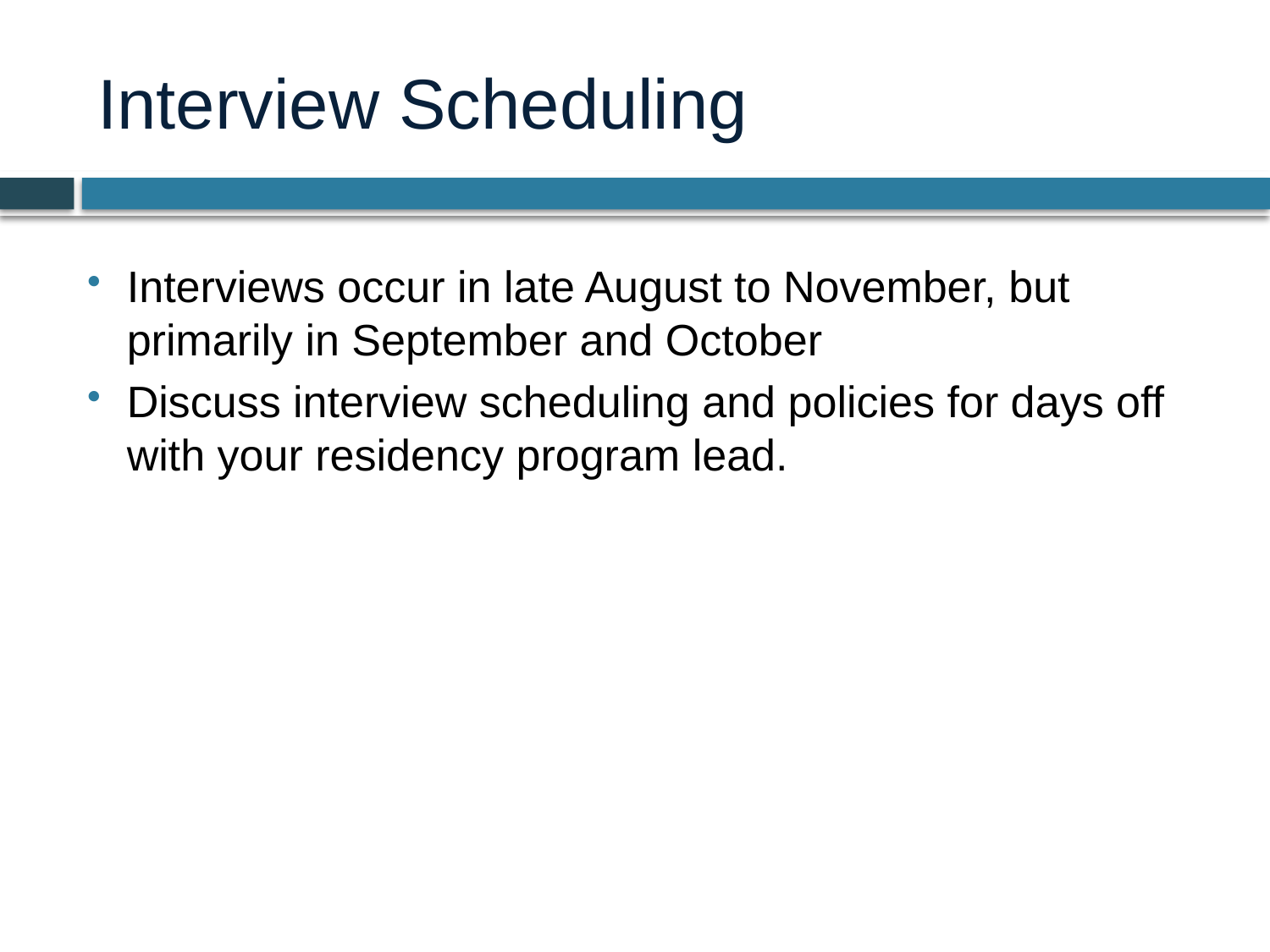

# Interview Scheduling
Interviews occur in late August to November, but primarily in September and October
Discuss interview scheduling and policies for days off with your residency program lead.

## Slide 6
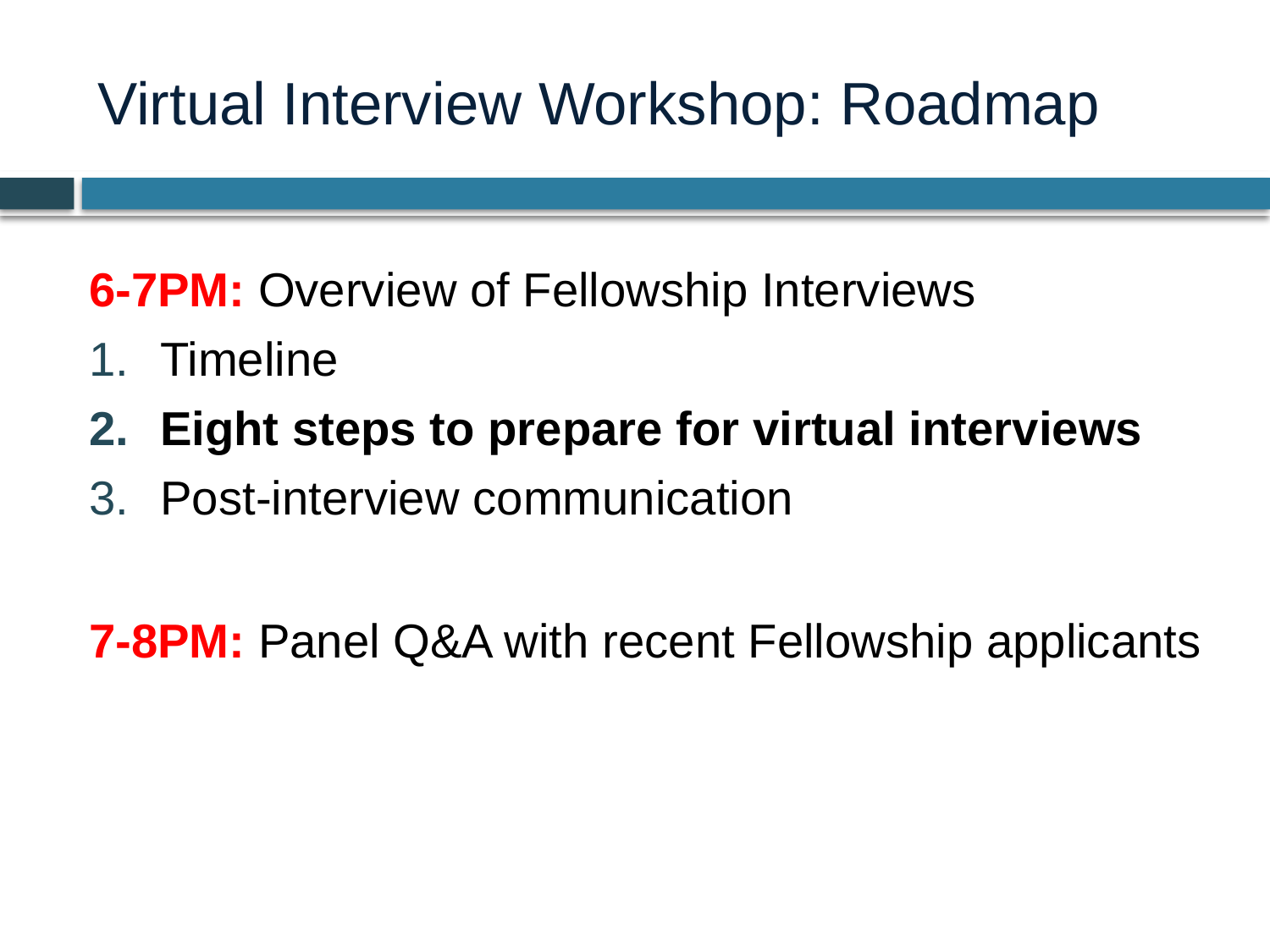

# Virtual Interview Workshop: Roadmap
6-7PM: Overview of Fellowship Interviews
Timeline
Eight steps to prepare for virtual interviews
Post-interview communication
7-8PM: Panel Q&A with recent Fellowship applicants

## Slide 7
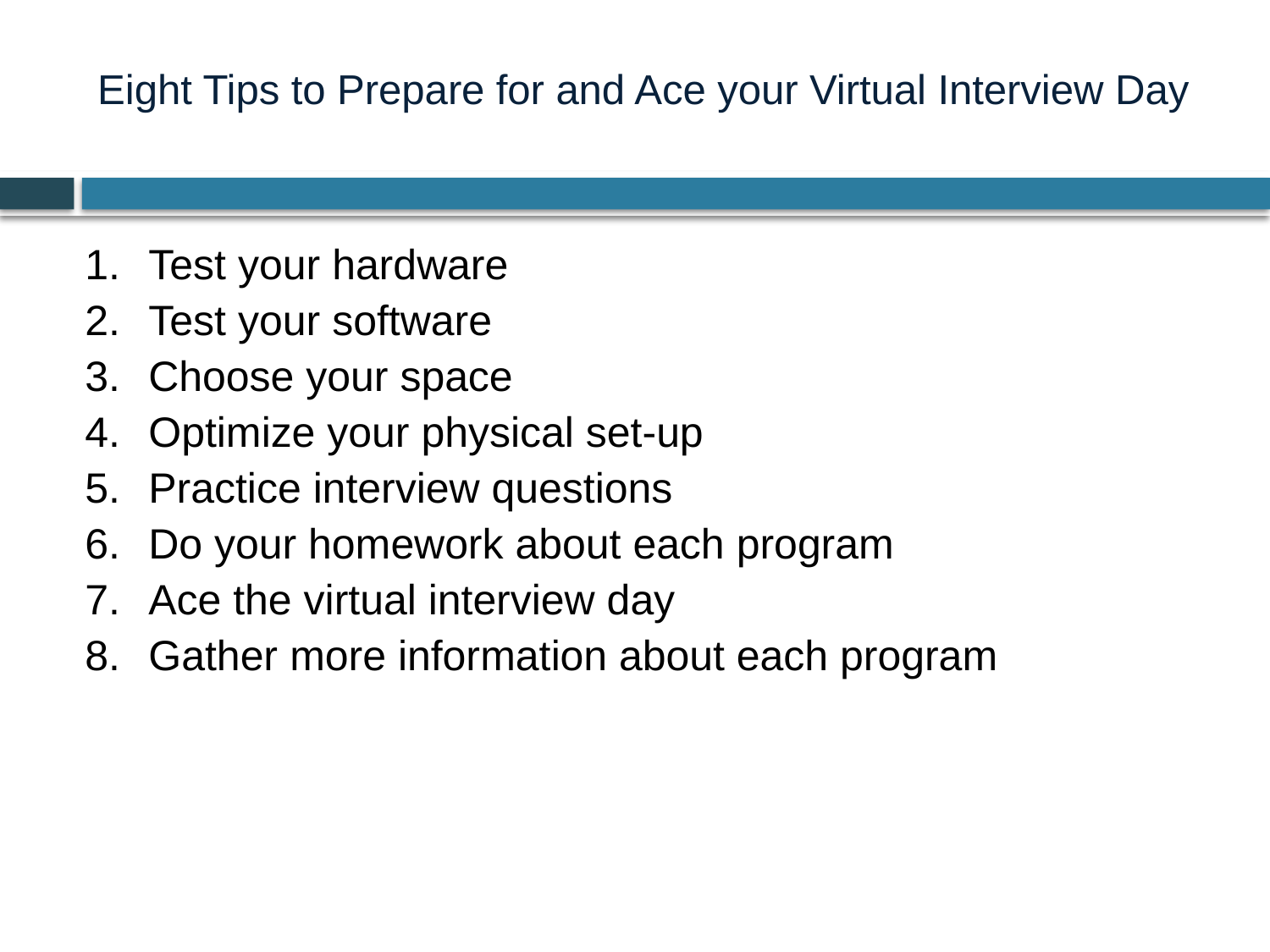

# Eight Tips to Prepare for and Ace your Virtual Interview Day
Test your hardware
Test your software
Choose your space
Optimize your physical set-up
Practice interview questions
Do your homework about each program
Ace the virtual interview day
Gather more information about each program

## Slide 8
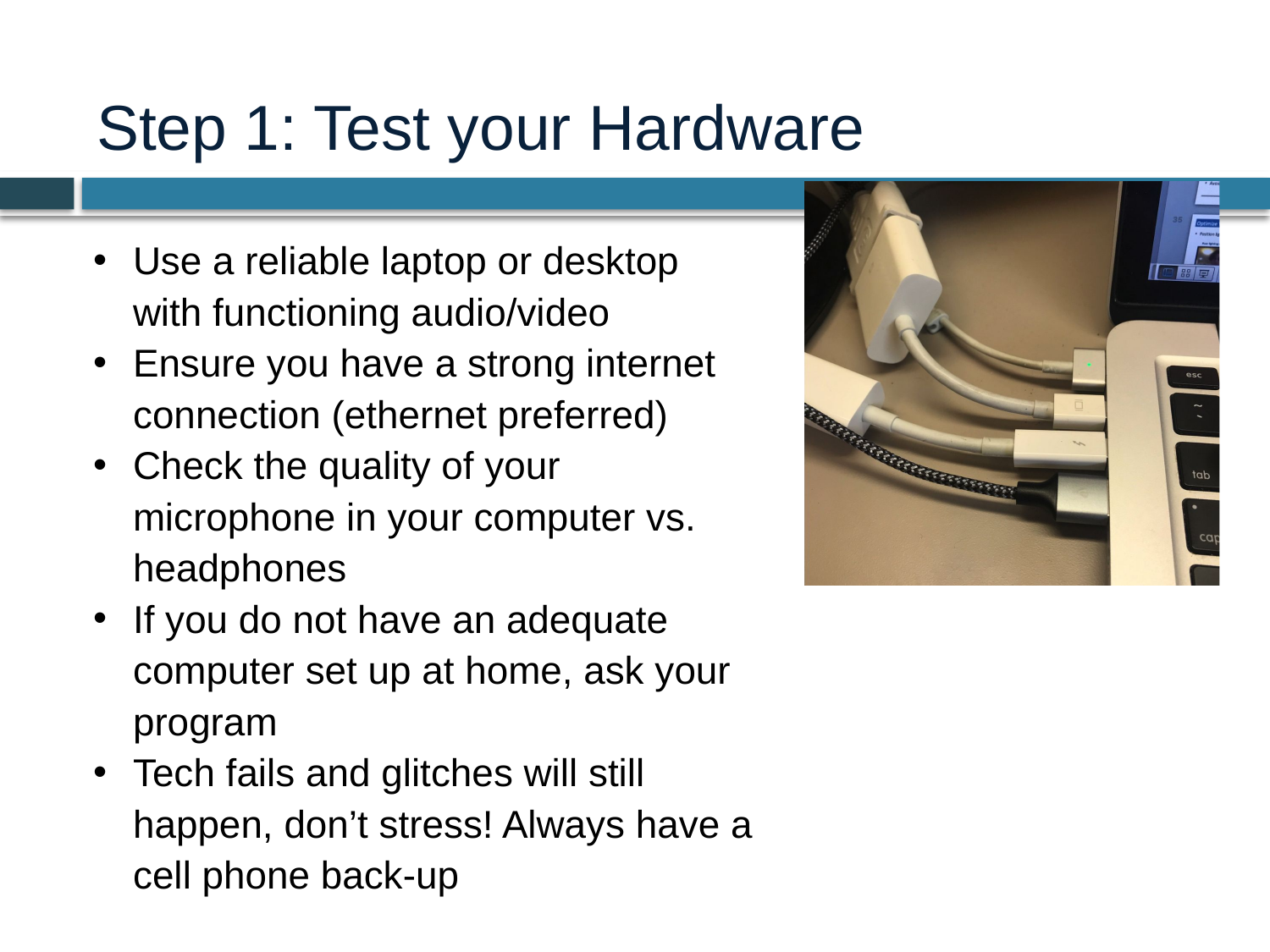

# Step 1: Test your Hardware
Use a reliable laptop or desktop with functioning audio/video
Ensure you have a strong internet connection (ethernet preferred)
Check the quality of your microphone in your computer vs. headphones
If you do not have an adequate computer set up at home, ask your program
Tech fails and glitches will still happen, don’t stress! Always have a cell phone back-up

## Slide 9
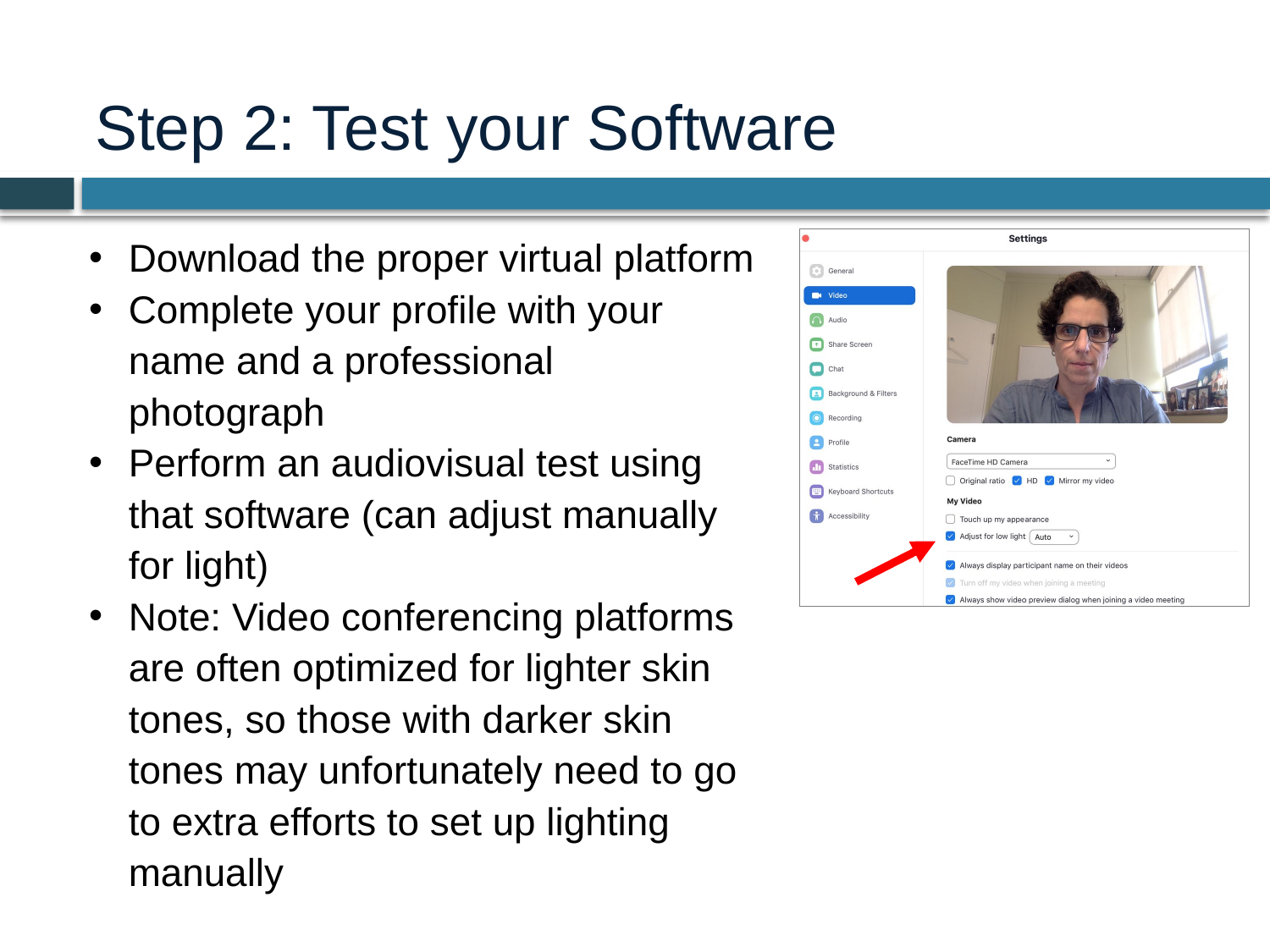

# Step 2: Test your Software
Download the proper virtual platform
Complete your profile with your name and a professional photograph
Perform an audiovisual test using that software (can adjust manually for light)
Note: Video conferencing platforms are often optimized for lighter skin tones, so those with darker skin tones may unfortunately need to go to extra efforts to set up lighting manually

## Slide 10
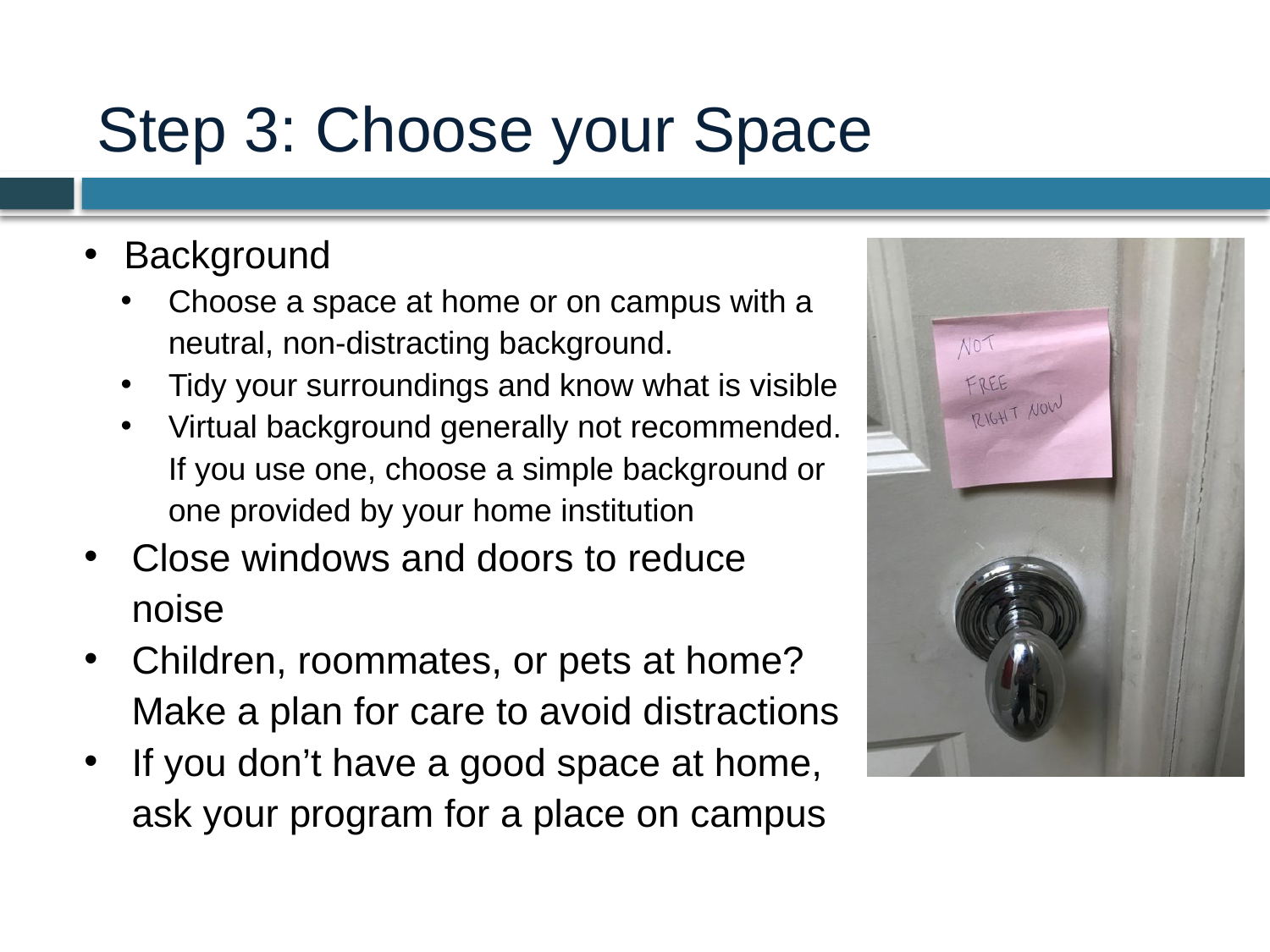

# Step 3: Choose your Space
Background
Choose a space at home or on campus with a neutral, non-distracting background.
Tidy your surroundings and know what is visible
Virtual background generally not recommended. If you use one, choose a simple background or one provided by your home institution
Close windows and doors to reduce noise
Children, roommates, or pets at home? Make a plan for care to avoid distractions
If you don’t have a good space at home, ask your program for a place on campus

## Slide 11
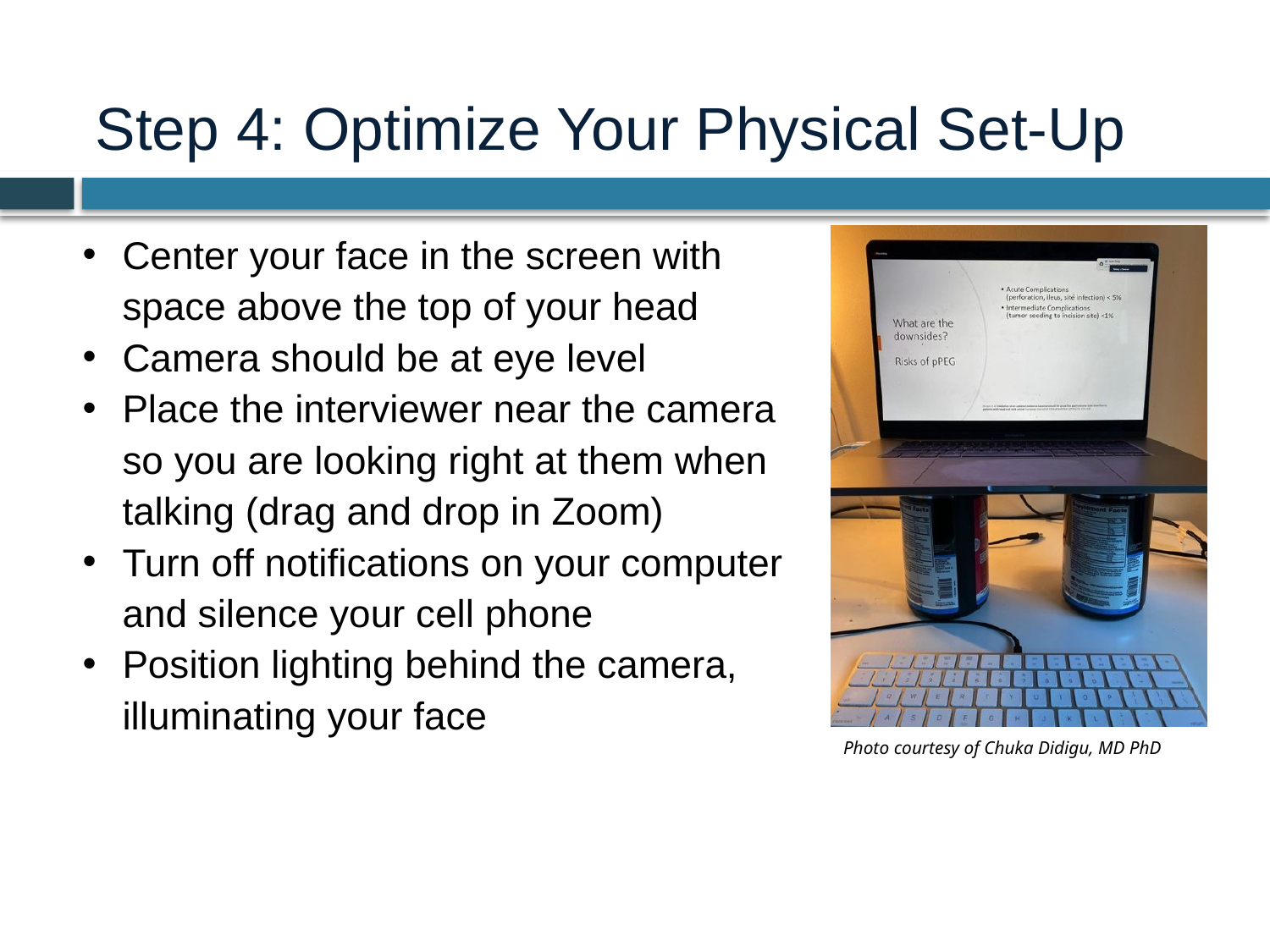

# Step 4: Optimize Your Physical Set-Up
Center your face in the screen with space above the top of your head
Camera should be at eye level
Place the interviewer near the camera so you are looking right at them when talking (drag and drop in Zoom)
Turn off notifications on your computer and silence your cell phone
Position lighting behind the camera, illuminating your face
Photo courtesy of Chuka Didigu, MD PhD

## Slide 12
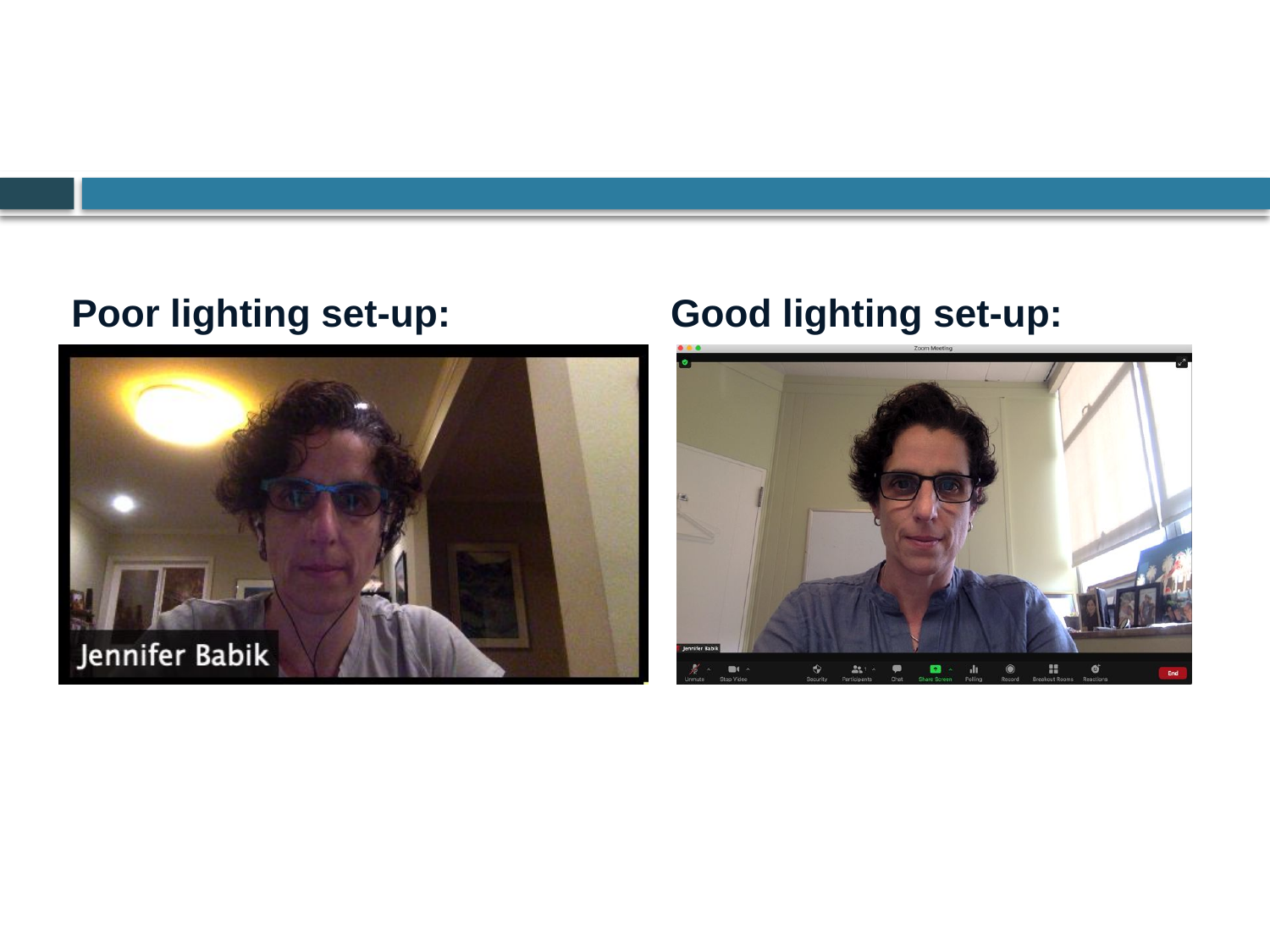

Poor lighting set-up:
Good lighting set-up:

## Slide 13
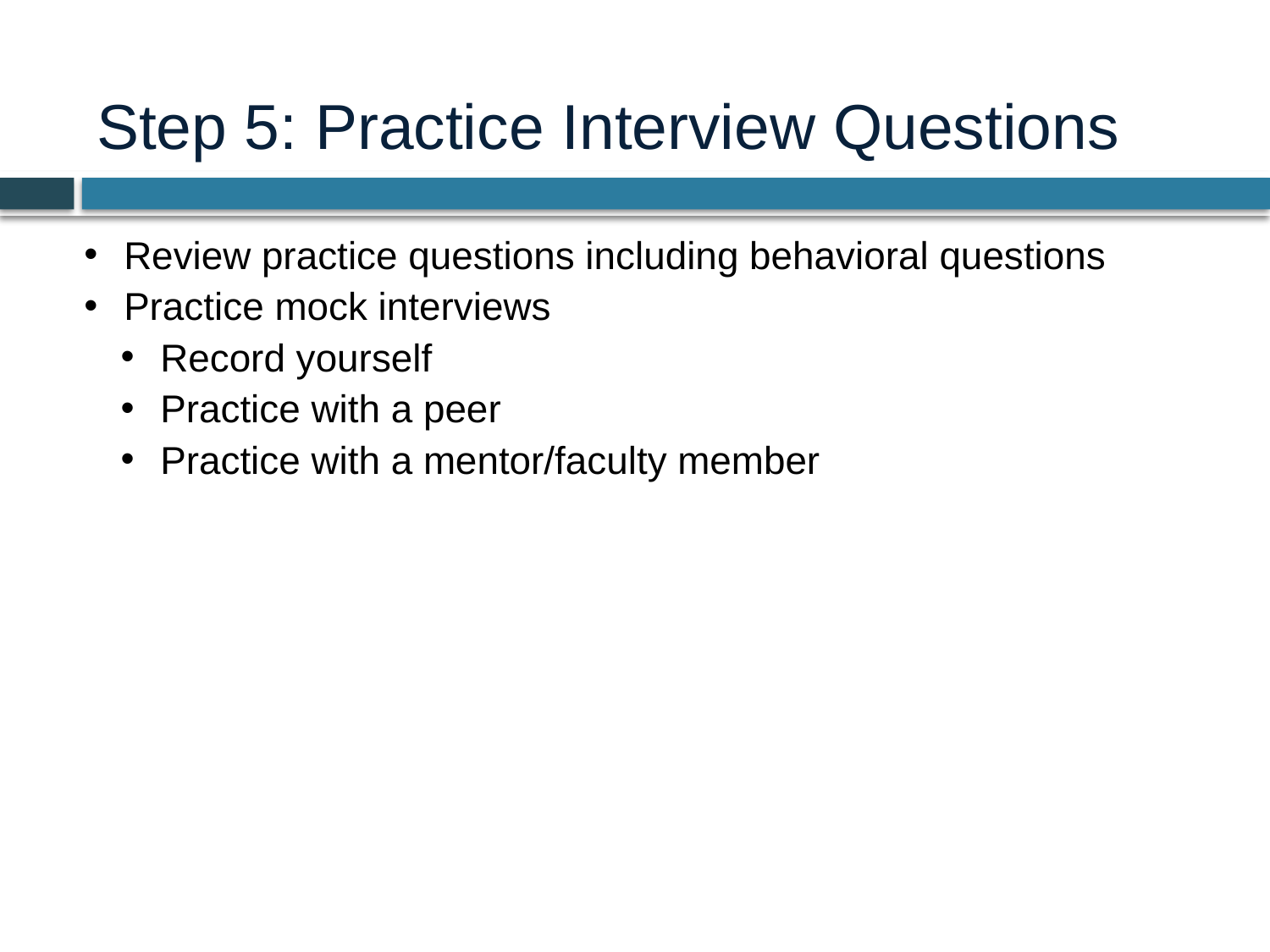

# Step 5: Practice Interview Questions
Review practice questions including behavioral questions
Practice mock interviews
Record yourself
Practice with a peer
Practice with a mentor/faculty member

## Slide 14
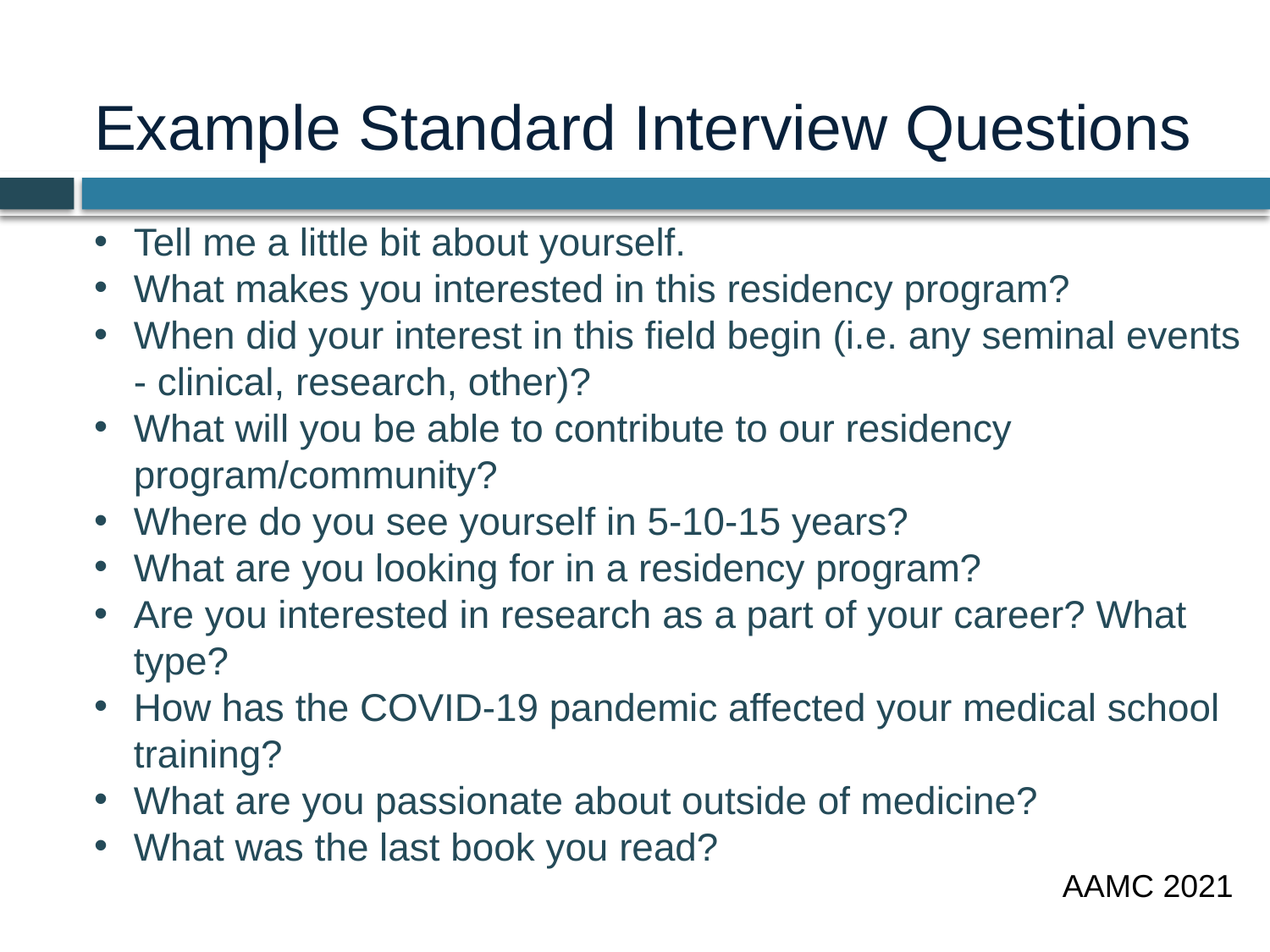

# Example Standard Interview Questions
Tell me a little bit about yourself.
What makes you interested in this residency program?
When did your interest in this field begin (i.e. any seminal events - clinical, research, other)?
What will you be able to contribute to our residency program/community?
Where do you see yourself in 5-10-15 years?
What are you looking for in a residency program?
Are you interested in research as a part of your career? What type?
How has the COVID-19 pandemic affected your medical school training?
What are you passionate about outside of medicine?
What was the last book you read?
AAMC 2021

## Slide 15
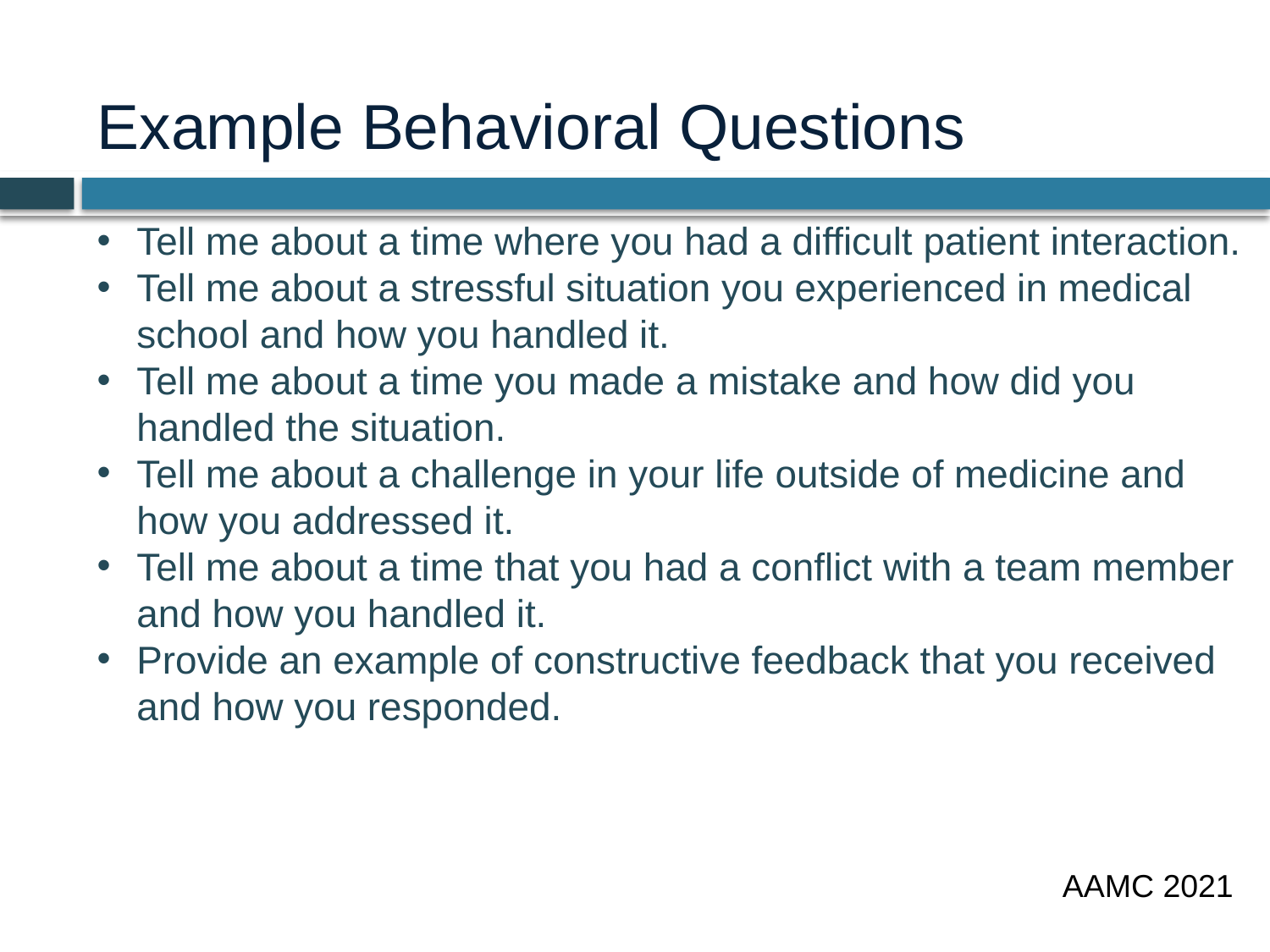

# Example Behavioral Questions
Tell me about a time where you had a difficult patient interaction.
Tell me about a stressful situation you experienced in medical school and how you handled it.
Tell me about a time you made a mistake and how did you handled the situation.
Tell me about a challenge in your life outside of medicine and how you addressed it.
Tell me about a time that you had a conflict with a team member and how you handled it.
Provide an example of constructive feedback that you received and how you responded.
AAMC 2021

## Slide 16
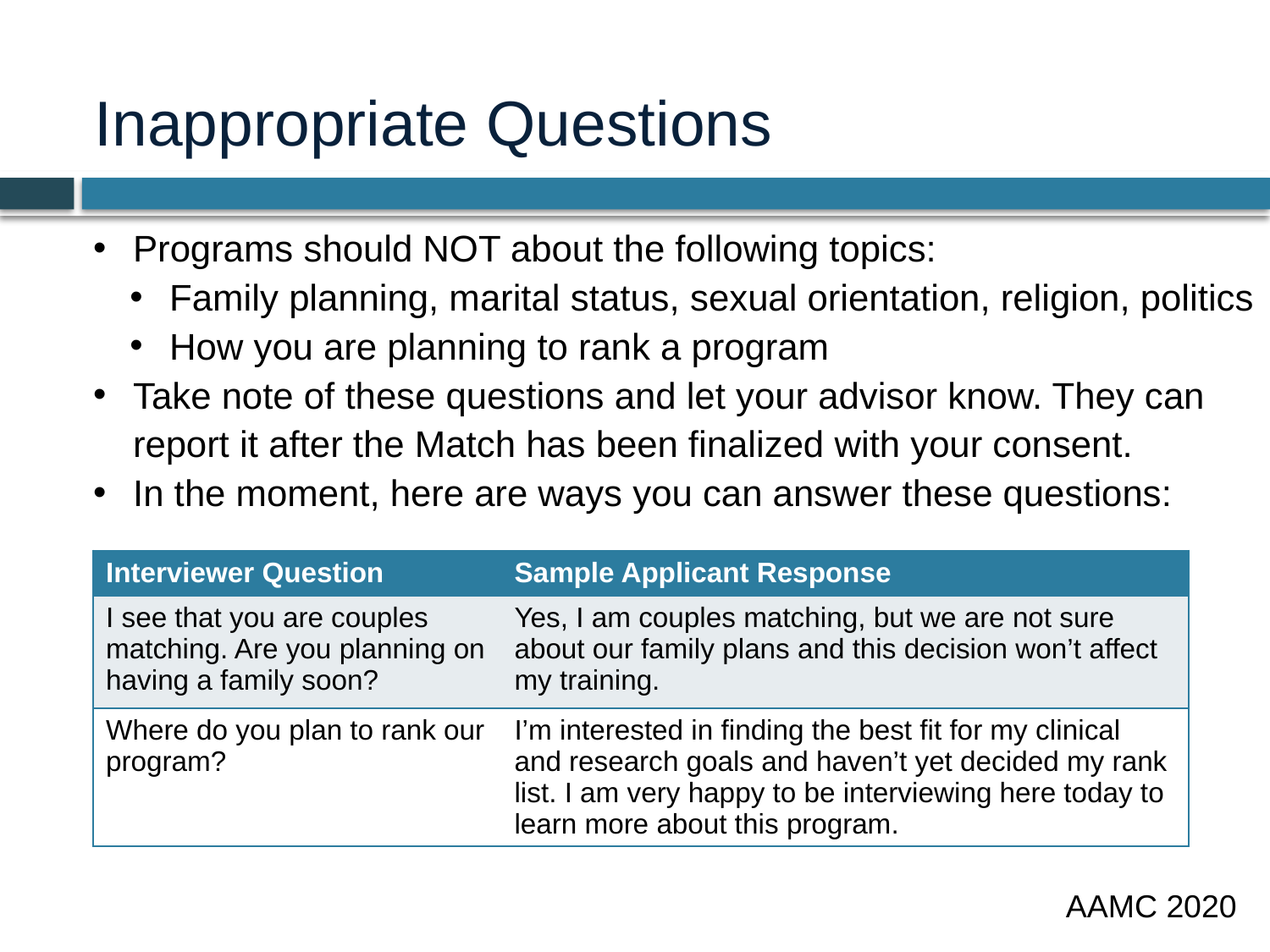

# Inappropriate Questions
Programs should NOT about the following topics:
Family planning, marital status, sexual orientation, religion, politics
How you are planning to rank a program
Take note of these questions and let your advisor know. They can report it after the Match has been finalized with your consent.
In the moment, here are ways you can answer these questions:
| Interviewer Question | Sample Applicant Response |
| --- | --- |
| I see that you are couples matching. Are you planning on having a family soon? | Yes, I am couples matching, but we are not sure about our family plans and this decision won’t affect my training. |
| Where do you plan to rank our program? | I’m interested in finding the best fit for my clinical and research goals and haven’t yet decided my rank list. I am very happy to be interviewing here today to learn more about this program. |
AAMC 2020

## Slide 17
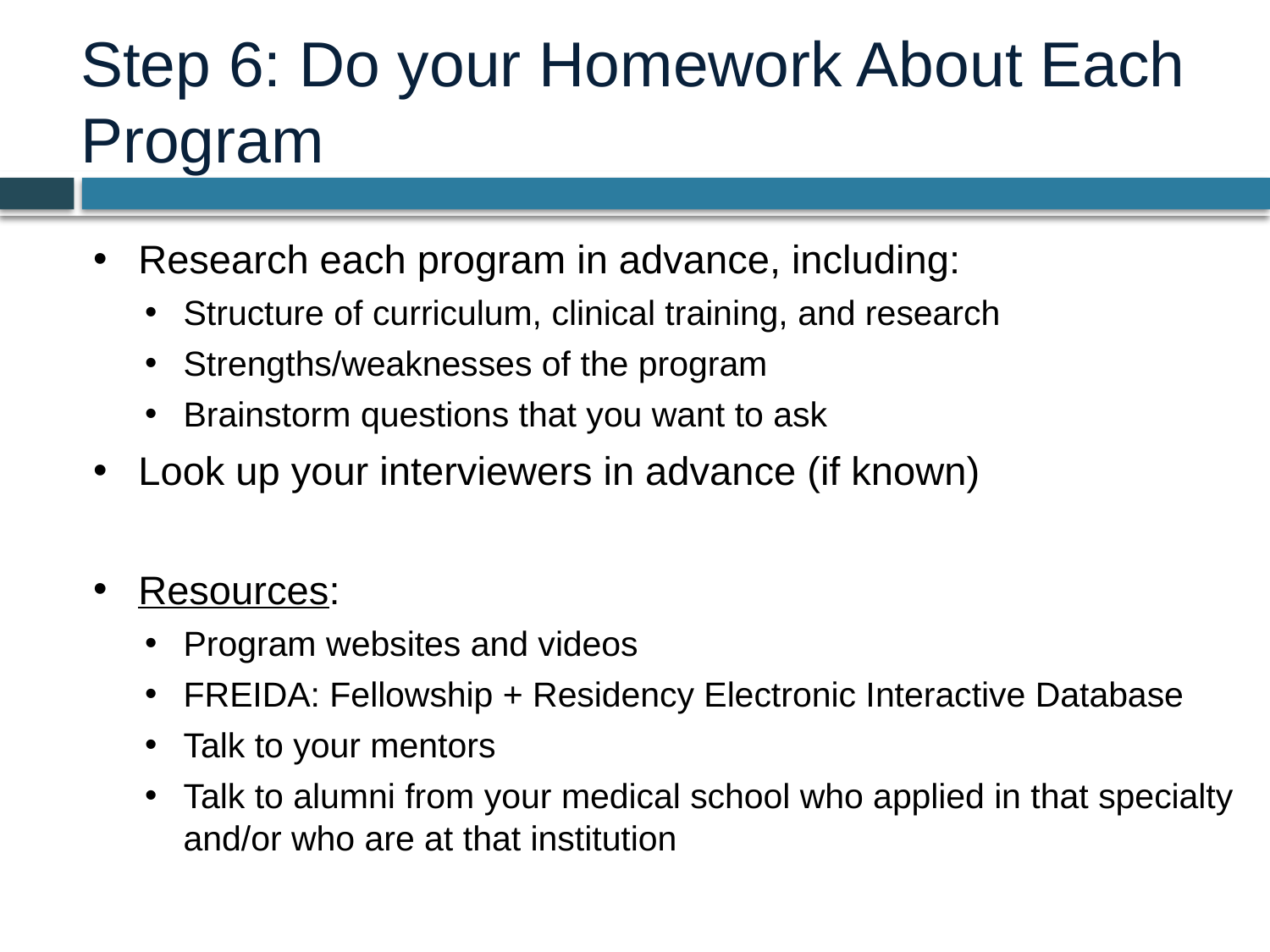

Step 6: Do your Homework About Each Program
Research each program in advance, including:
Structure of curriculum, clinical training, and research
Strengths/weaknesses of the program
Brainstorm questions that you want to ask
Look up your interviewers in advance (if known)
Resources:
Program websites and videos
FREIDA: Fellowship + Residency Electronic Interactive Database
Talk to your mentors
Talk to alumni from your medical school who applied in that specialty and/or who are at that institution

## Slide 18
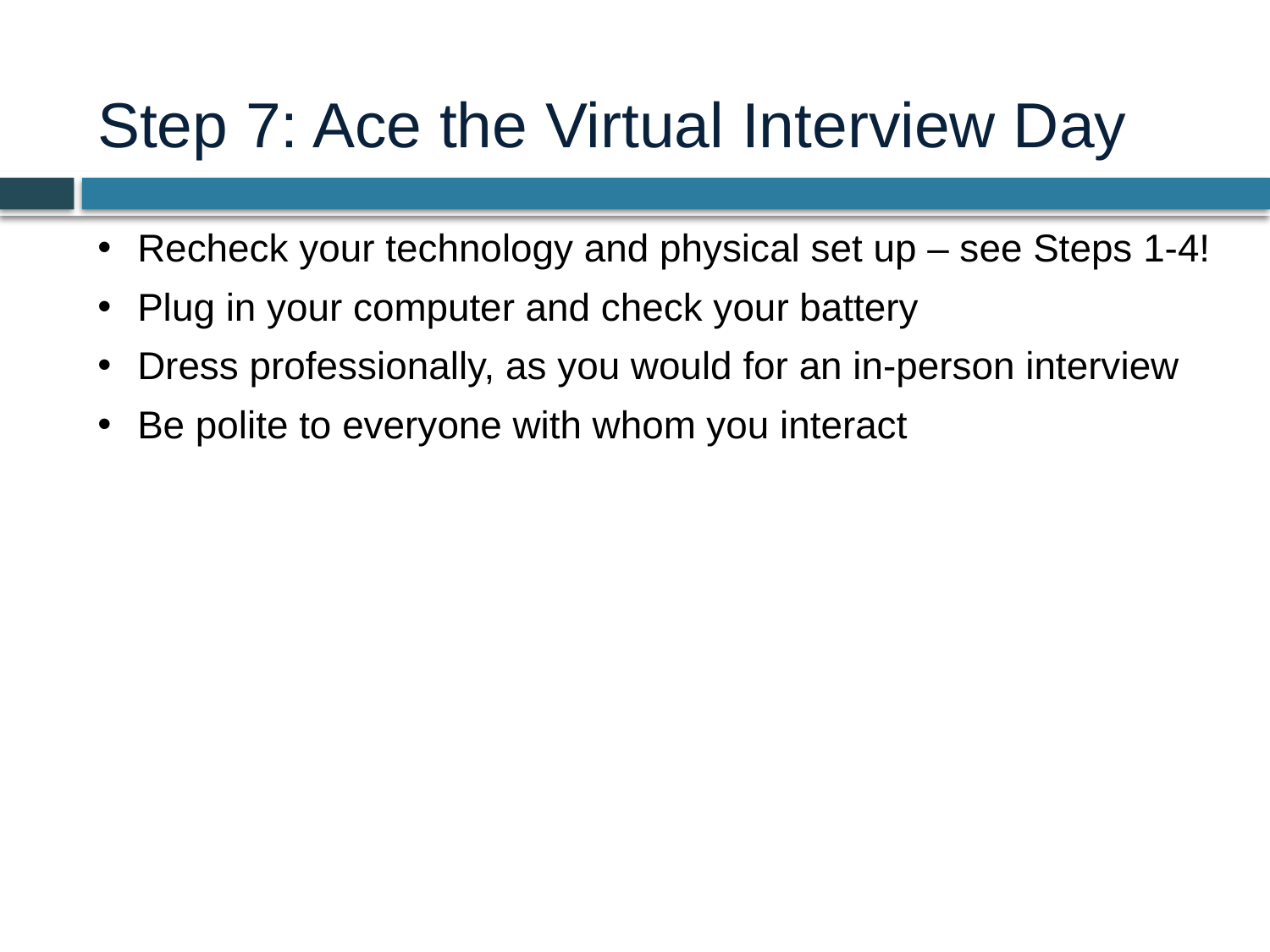

# Step 7: Ace the Virtual Interview Day
Recheck your technology and physical set up – see Steps 1-4!
Plug in your computer and check your battery
Dress professionally, as you would for an in-person interview
Be polite to everyone with whom you interact

## Slide 19
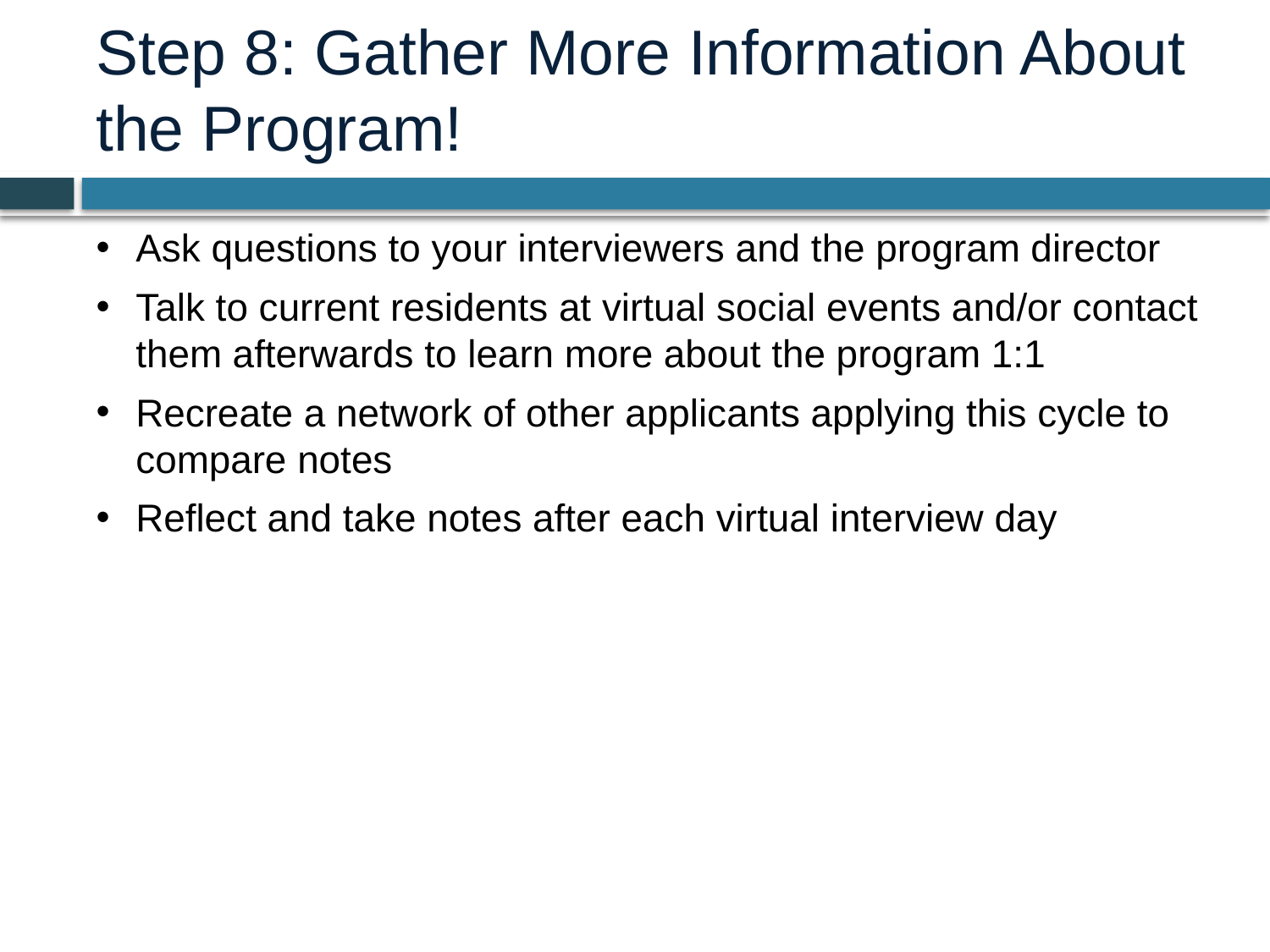

# Step 8: Gather More Information About the Program!
Ask questions to your interviewers and the program director
Talk to current residents at virtual social events and/or contact them afterwards to learn more about the program 1:1
Recreate a network of other applicants applying this cycle to compare notes
Reflect and take notes after each virtual interview day

## Slide 20
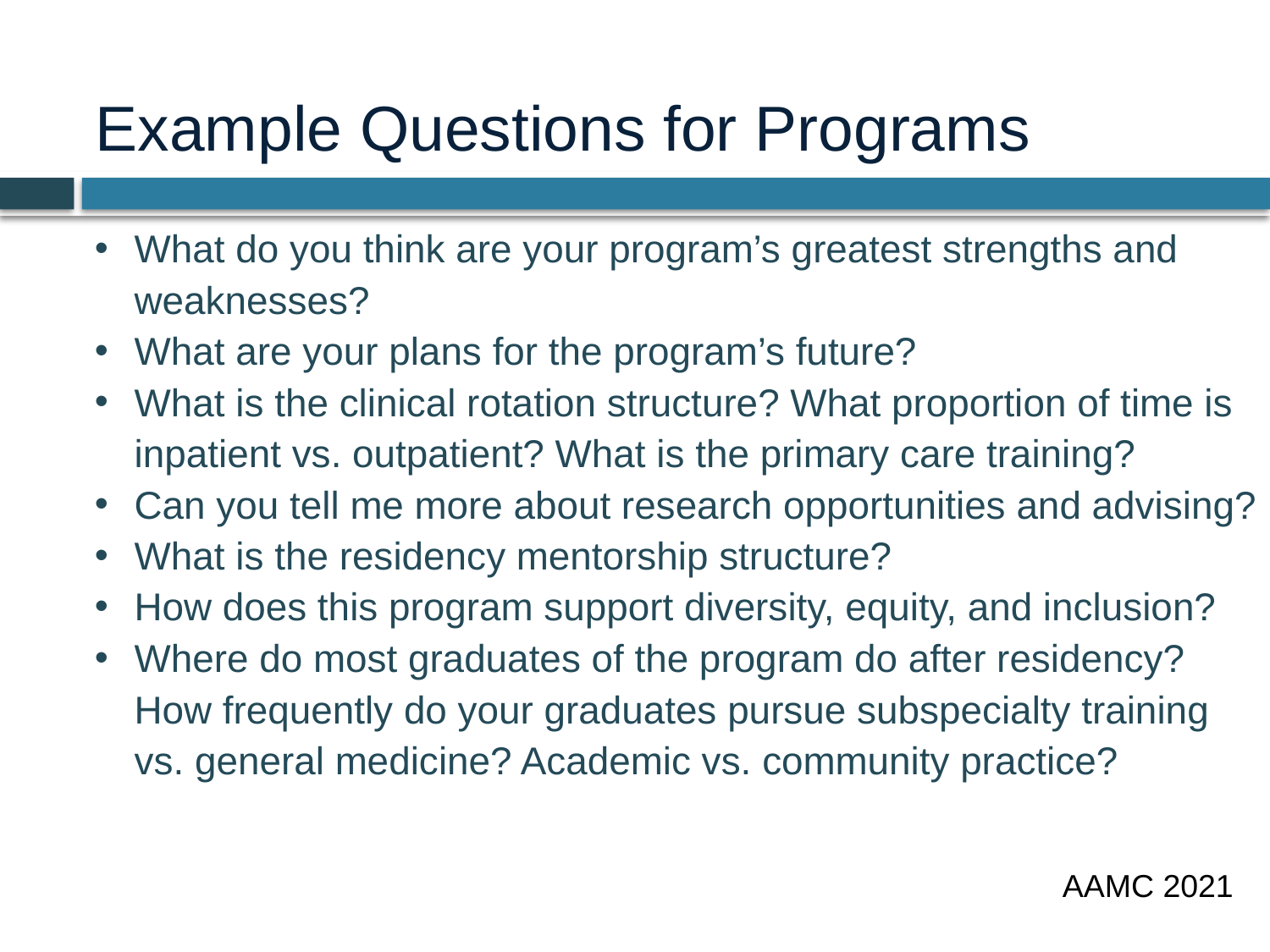

# Example Questions for Programs
What do you think are your program’s greatest strengths and weaknesses?
What are your plans for the program’s future?
What is the clinical rotation structure? What proportion of time is inpatient vs. outpatient? What is the primary care training?
Can you tell me more about research opportunities and advising?
What is the residency mentorship structure?
How does this program support diversity, equity, and inclusion?
Where do most graduates of the program do after residency? How frequently do your graduates pursue subspecialty training vs. general medicine? Academic vs. community practice?
AAMC 2021

## Slide 21
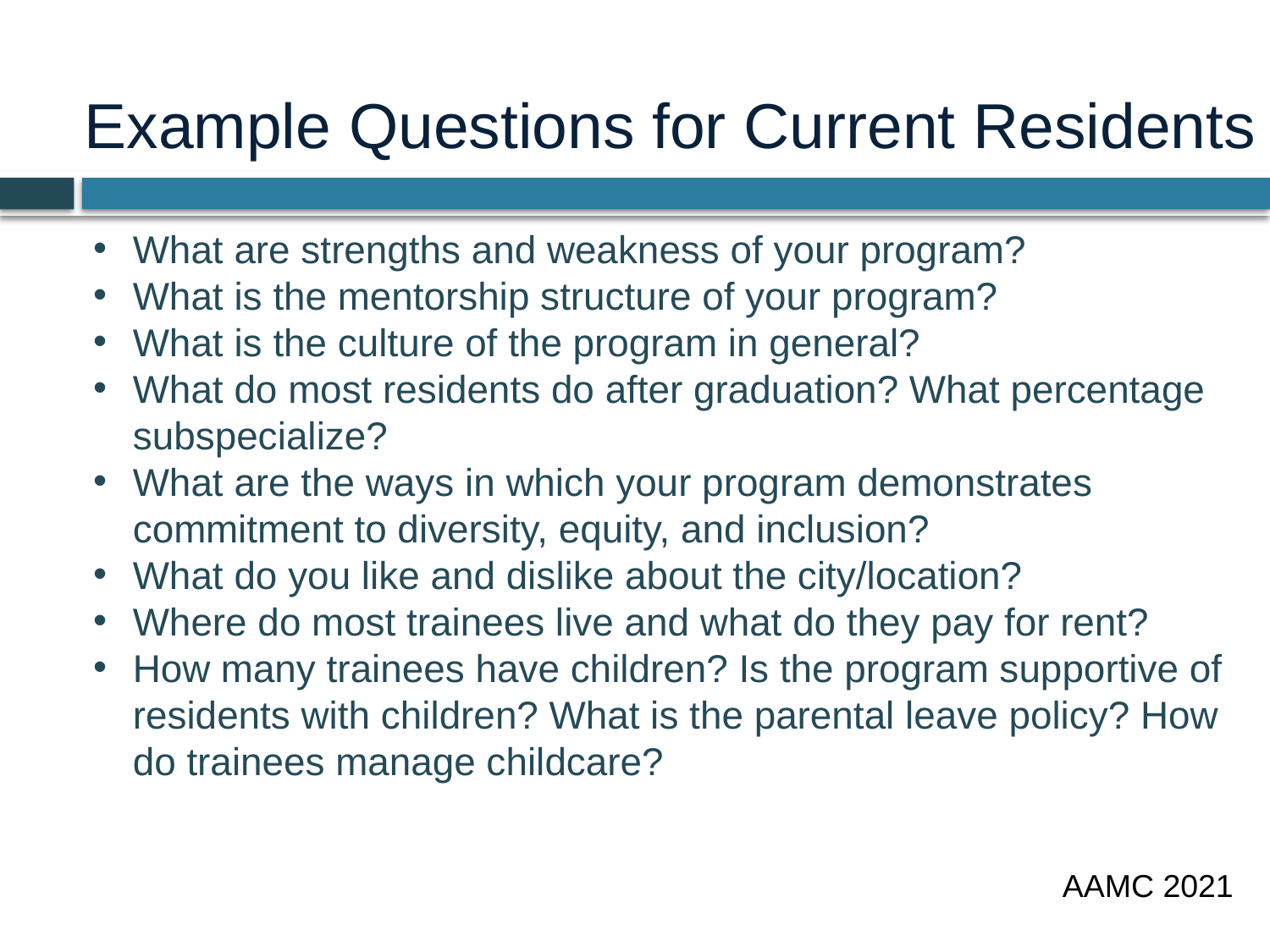

# Example Questions for Current Residents
What are strengths and weakness of your program?
What is the mentorship structure of your program?
What is the culture of the program in general?
What do most residents do after graduation? What percentage subspecialize?
What are the ways in which your program demonstrates commitment to diversity, equity, and inclusion?
What do you like and dislike about the city/location?
Where do most trainees live and what do they pay for rent?
How many trainees have children? Is the program supportive of residents with children? What is the parental leave policy? How do trainees manage childcare?
AAMC 2021

## Slide 22
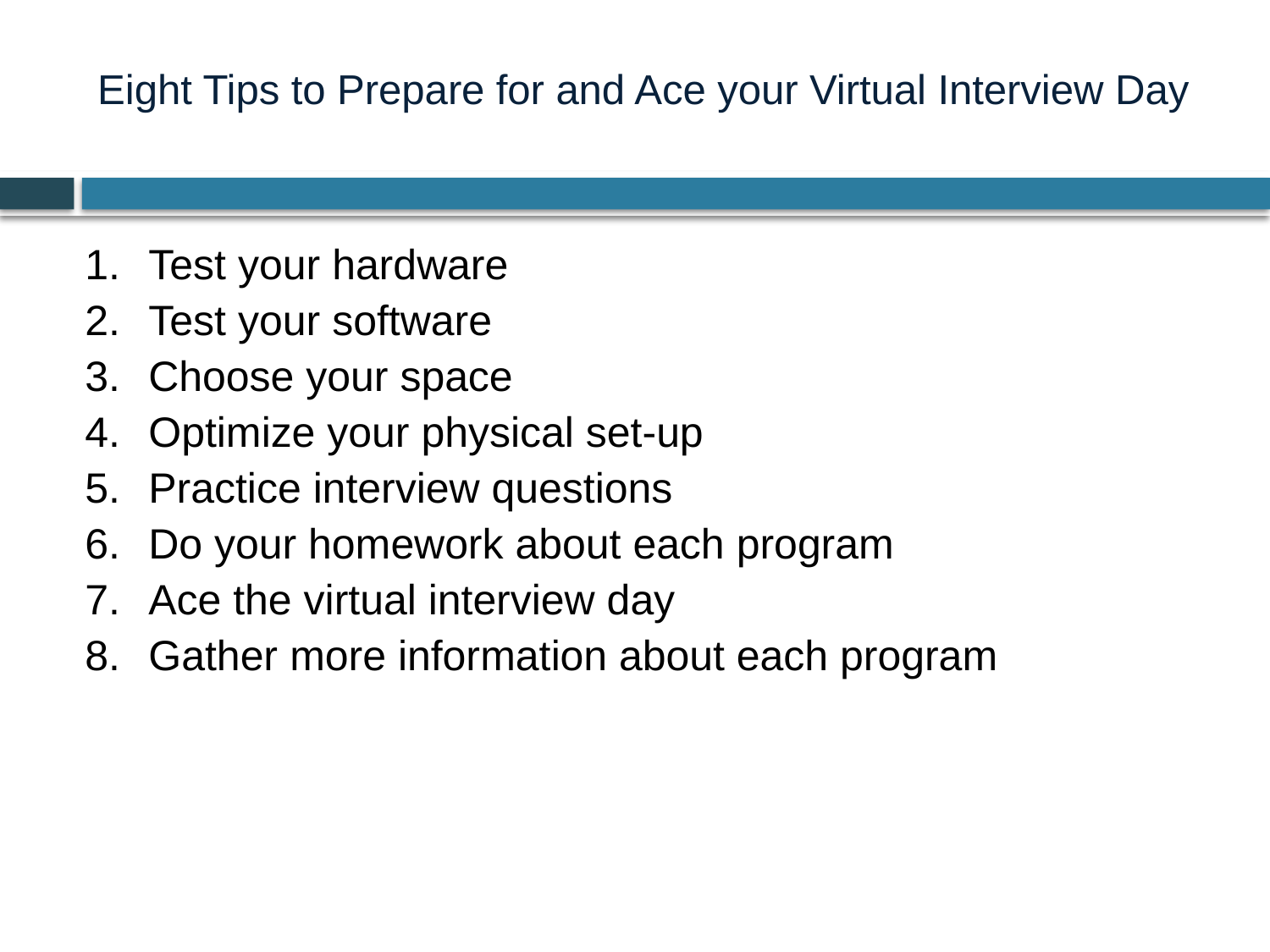

# Eight Tips to Prepare for and Ace your Virtual Interview Day
Test your hardware
Test your software
Choose your space
Optimize your physical set-up
Practice interview questions
Do your homework about each program
Ace the virtual interview day
Gather more information about each program

## Slide 23
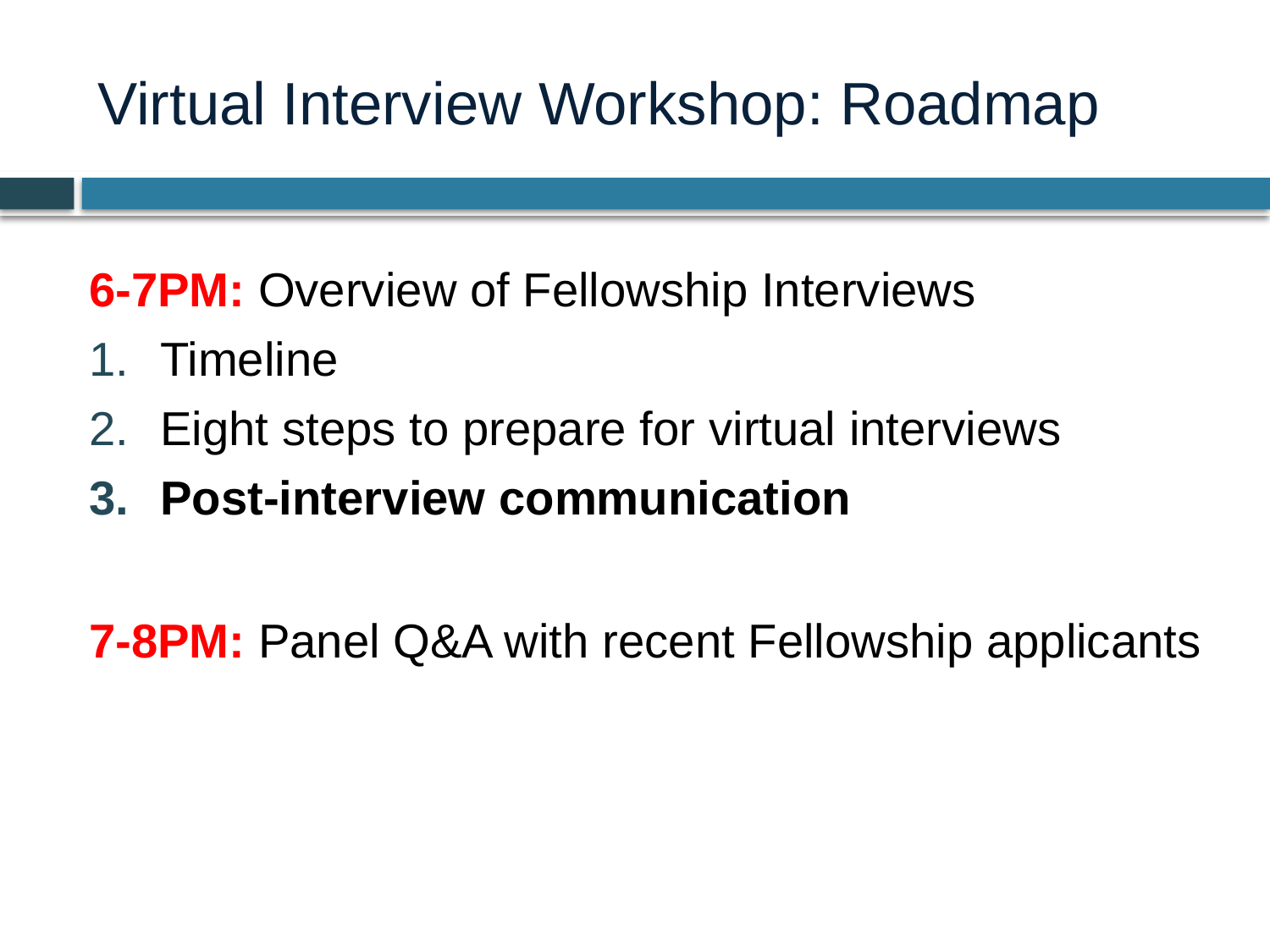

# Virtual Interview Workshop: Roadmap
6-7PM: Overview of Fellowship Interviews
Timeline
Eight steps to prepare for virtual interviews
Post-interview communication
7-8PM: Panel Q&A with recent Fellowship applicants

## Slide 24
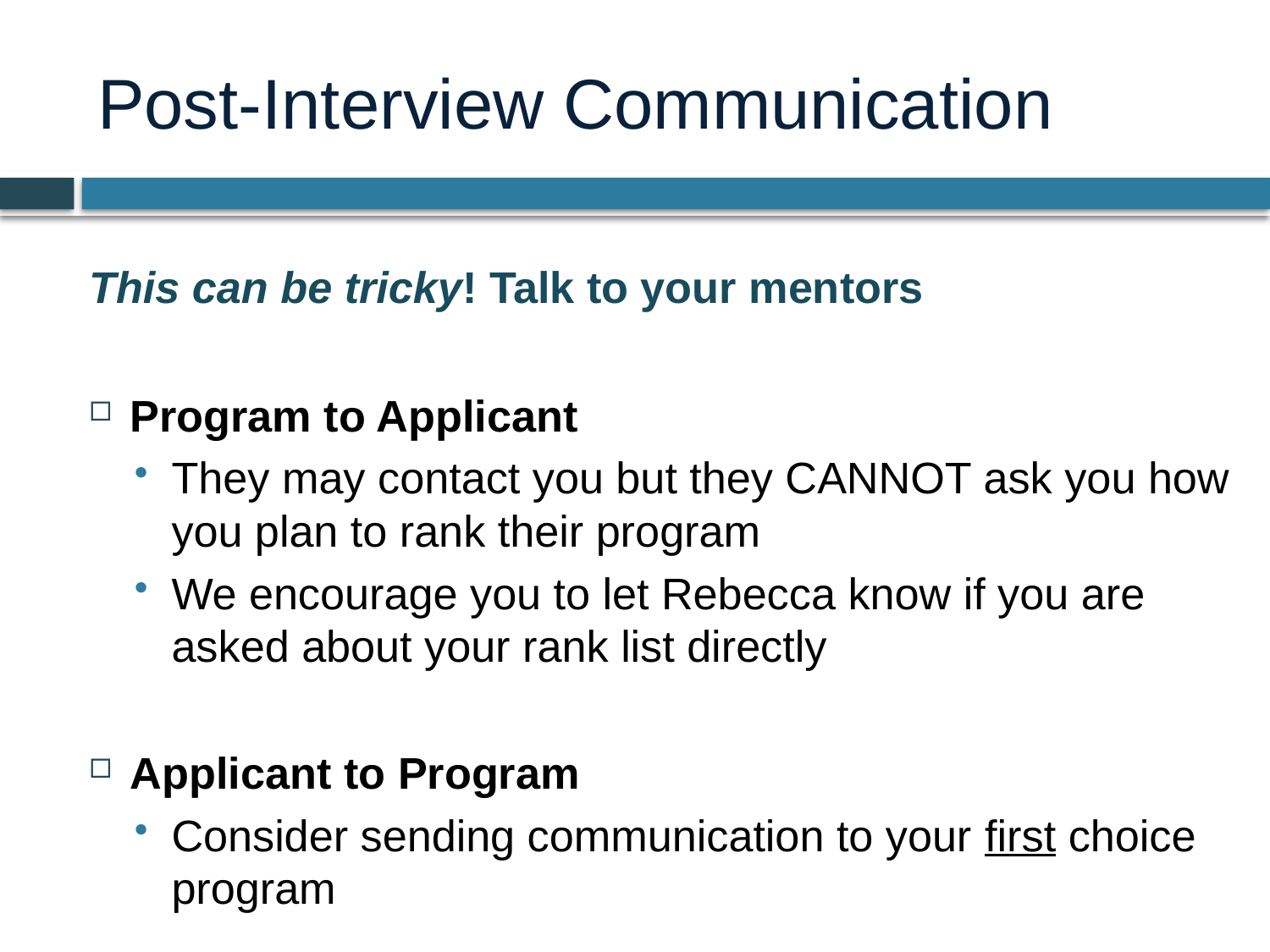

# Post-Interview Communication
This can be tricky! Talk to your mentors
Program to Applicant
They may contact you but they CANNOT ask you how you plan to rank their program
We encourage you to let Rebecca know if you are asked about your rank list directly
Applicant to Program
Consider sending communication to your first choice program

## Slide 25
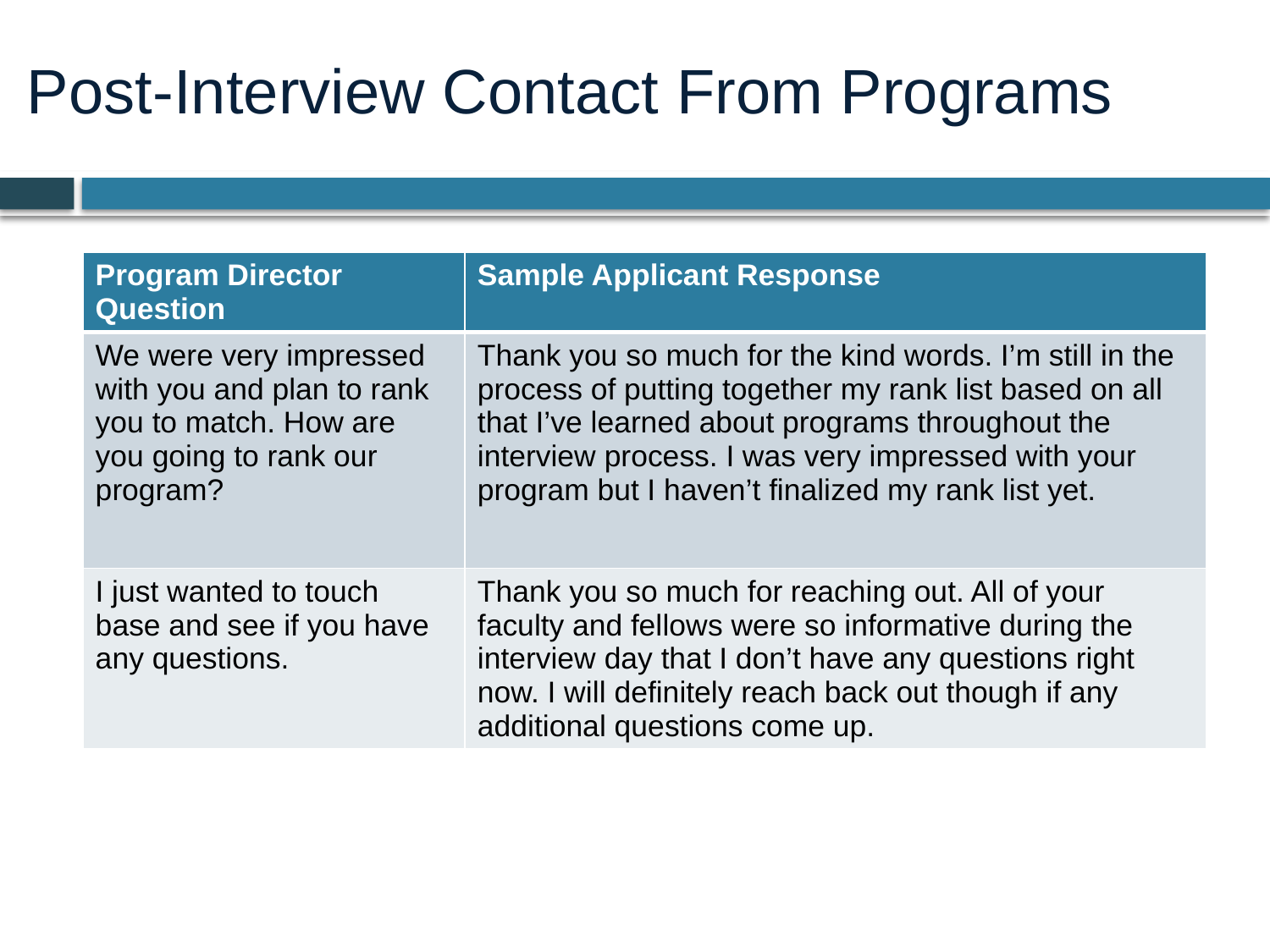

# Post-Interview Contact From Programs
| Program Director Question | Sample Applicant Response |
| --- | --- |
| We were very impressed with you and plan to rank you to match. How are you going to rank our program? | Thank you so much for the kind words. I’m still in the process of putting together my rank list based on all that I’ve learned about programs throughout the interview process. I was very impressed with your program but I haven’t finalized my rank list yet. |
| I just wanted to touch base and see if you have any questions. | Thank you so much for reaching out. All of your faculty and fellows were so informative during the interview day that I don’t have any questions right now. I will definitely reach back out though if any additional questions come up. |

## Slide 26
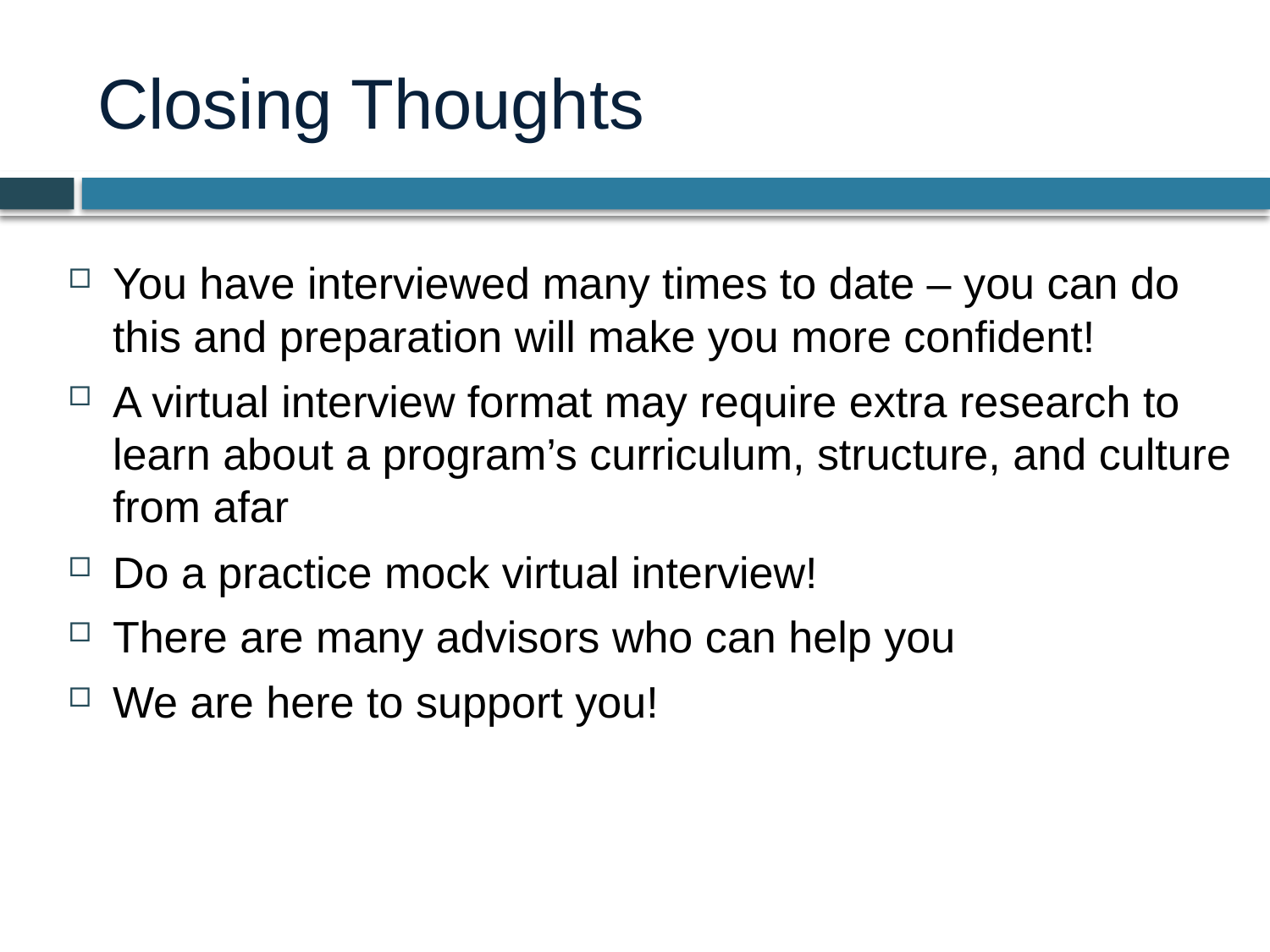

# Closing Thoughts
You have interviewed many times to date – you can do this and preparation will make you more confident!
A virtual interview format may require extra research to learn about a program’s curriculum, structure, and culture from afar
Do a practice mock virtual interview!
There are many advisors who can help you
We are here to support you!

## Slide 27
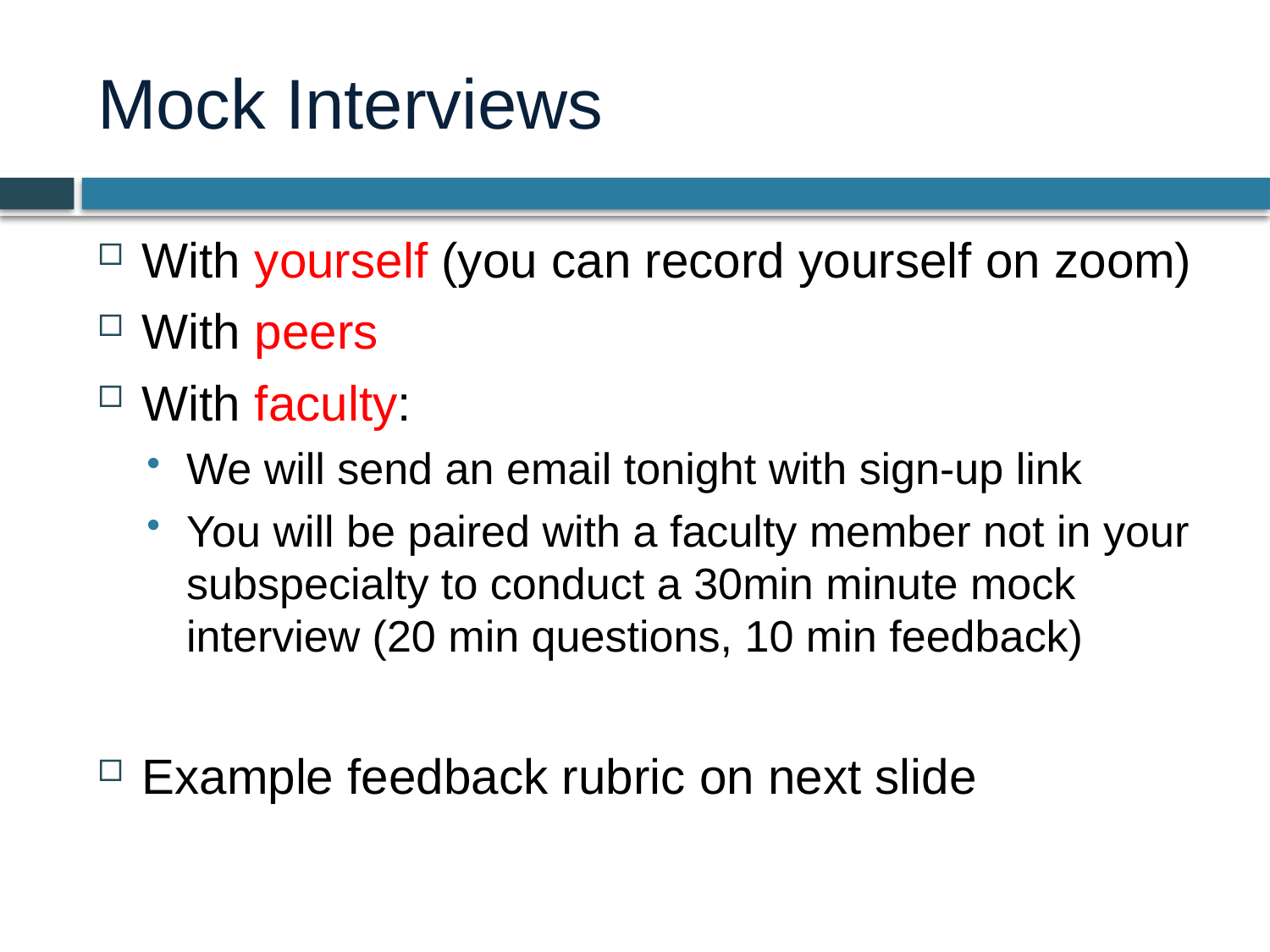

# Mock Interviews
With yourself (you can record yourself on zoom)
With peers
With faculty:
We will send an email tonight with sign-up link
You will be paired with a faculty member not in your subspecialty to conduct a 30min minute mock interview (20 min questions, 10 min feedback)
Example feedback rubric on next slide

## Slide 28
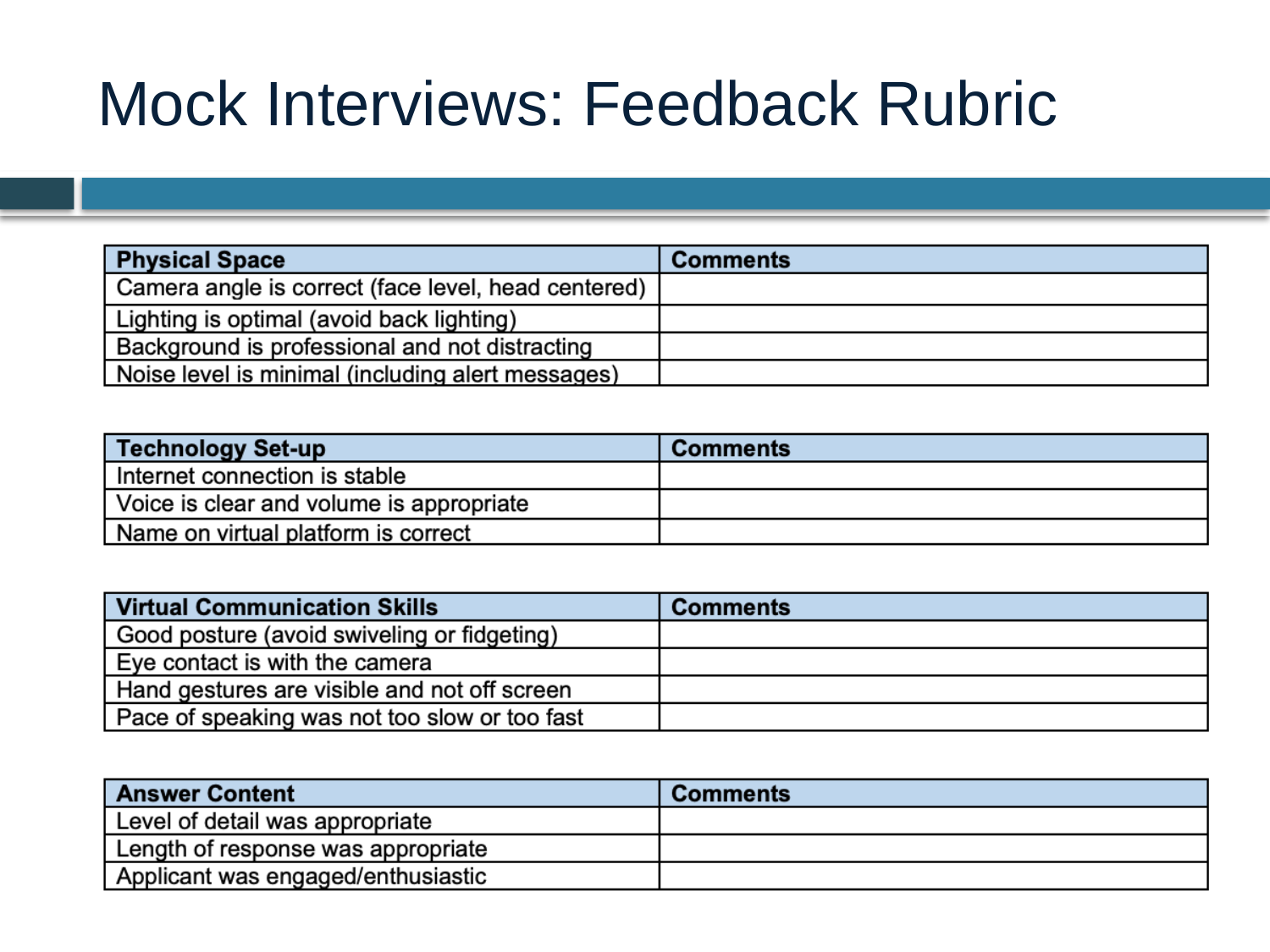

# Mock Interviews: Feedback Rubric

## Slide 29
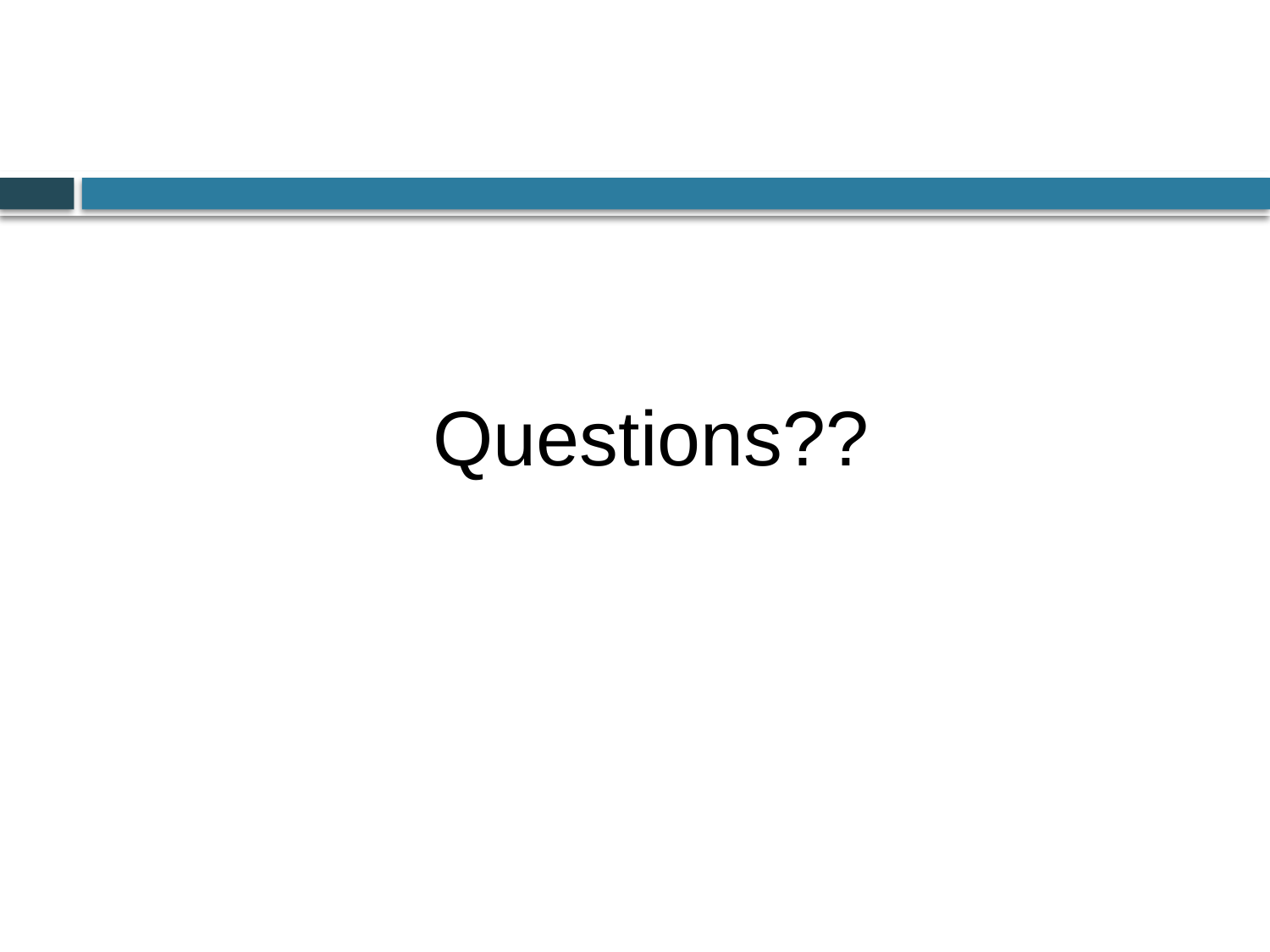

Questions??

## Slide 30
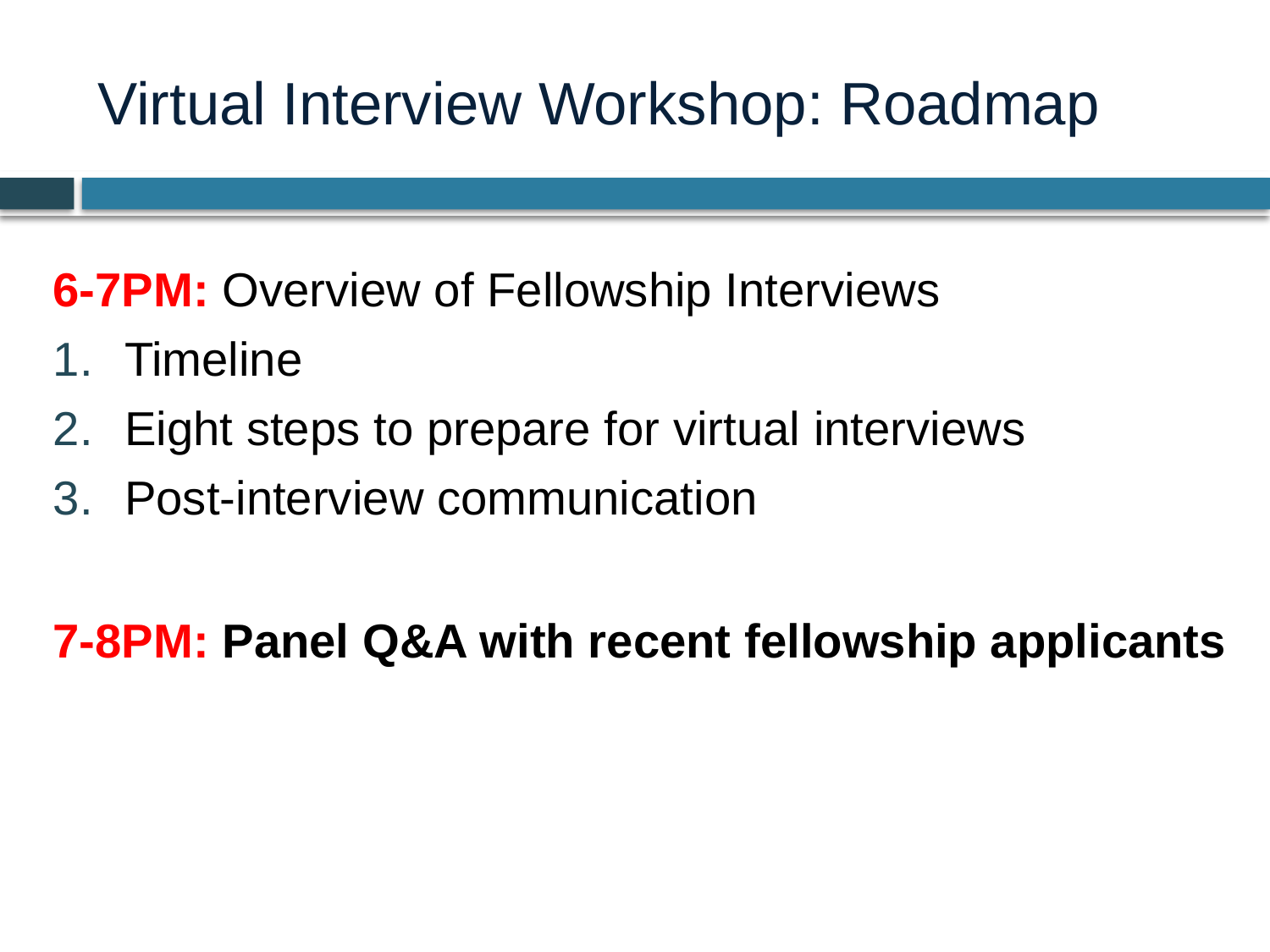

# Virtual Interview Workshop: Roadmap
6-7PM: Overview of Fellowship Interviews
Timeline
Eight steps to prepare for virtual interviews
Post-interview communication
7-8PM: Panel Q&A with recent fellowship applicants
