# Supplementary material for: Development and Evaluation of a Five-Component Toolkit for Internal Medicine Residents Applying for Subspecialty Fellowships
Source: MedEdPORTAL. 2022 Mar 14;18:11228. doi: 10.15766/mep_2374-8265.11228 (PMC8918571; doi:10.15766/mep_2374-8265.11228)
Supplement: Supplementary file 1 — Elements of the Fellowship Application Toolkit.docxFellowship Application Guide.docxFellowship Application Information Night.pptxSubspecialty Breakout Room Questions.docxPreparing for Virtual Interviews.pptxMock Virtual Interview.docxSurvey Instrument.docx [file mep_2374-8265.11228-s001.zip › Educational_Summary_Report_11228.pdf]

MedEdPORTAL® The AAMC Journal of Teaching and Learning Resources

Original Publication Open Access

Development and Evaluation of a Five-Component Toolkit for Internal Medicine Residents Applying for Subspecialty Fellowships

Laura A. Huppert, MD*, Jennifer M. Babik, MD, PhD

*Corresponding author: laura.huppert@ucsf.edu

Abstract

Introduction: Subspecialty fellowship is a common career path for internal medicine (IM) residents, but little is published on residency program curricula for guiding residents through the process of applying to subspecialty fellowships. We describe a toolkit to guide IM residents through this process. **Methods:** We developed and implemented the Fellowship Application Toolkit for IM residents at the University of California, San Francisco, from 2018 to 2020. Educational strategies included live workshops, written resources, and one-to-one coaching, consisting of five components: fellowship application guidebook, Fellowship Application Information Night, alumni contact list, personal statement resources and coaches, and virtual interview workshop and mock interviews. Residents were surveyed both pre- and postintervention to evaluate these resources' use and efficacy. **Results:** Survey response rates were 21 of 41 (51%) in 2018, 25 of 41 (61%) in 2019, and 24 of 43 (56%) in 2020. Most respondents indicated the resources were extremely or very effective, including 30 of 36 (83%) who used the guidebook, 31 of 37 (84%) who attended the Fellowship Application Information Night, 10 of 15 (67%) who used the alumni contact list, nine of 10 (90%) who used the personal statement resources, and 12 of 14 (86%) who attended the virtual interview workshop. Respondents strongly or somewhat agreed that the overall efficacy of the residency's fellowship advising improved from pre- to postintervention (four of 17 [24%] in 2018 vs. 17 of 21 [81%] in 2020, $p < .001$). **Discussion:** Our Fellowship Application Toolkit was effective at supporting IM residents applying to fellowships.

Keywords
Residency, Fellowship, Interview, Fellowship Application, Program Evaluation

Educational Objectives

By the end of this activity, learners will be able to:

1. Describe the timeline and components of the fellowship application process.
2. Compose a personal statement using a defined template and specialty-specific examples.
3. Prepare for interviews and negotiate postinterview communication.
4. Connect with an alumni network at other institutions.

Introduction

The majority of internal medicine (IM) residents in the United States report subspecialty career plans.[1] During the 2021 ERAS application cycle, over eight thousand residents applied to subspecialty fellowships, a number that has increased over the past 5 years.[2,3] Despite this common career choice for IM residents, there is little published about how residency programs can best guide residents through the fellowship application process.

There are only a few published resources offering guidance for residents about how to independently prepare for fellowship interviews. For example, Bosslet and colleagues have provided an excellent structured approach to help residents prepare for the pulmonary/critical care medicine fellowship application process.[4] In addition, several resources list common interview questions and provide interview tips.[5-7] However, residents desire more formal guidance on the process, preferably incorporated into the residency program curriculum.[8]

There is limited data about how programs can best support residents who are applying for subspecialty fellowship. Some medical schools have designed programs to help medical students applying to residency,[9,10] but the fellowship application

Citation:
Huppert LA, Babik JM. Development and evaluation of a five-component toolkit for internal medicine residents applying for subspecialty fellowships. *MedEdPORTAL*. 2022;18:11228.
https://doi.org/10.15766/mep_2374-8265.11228

Copyright © 2022 Huppert and Babik. This is an open-access publication distributed under the terms of the Creative Commons Attribution license.

process has important distinctions, including increased postinterview communication, the expectation for a more defined career vision, and additional career and/or personal considerations. A study by Sinclair and colleagues described the introduction of a didactic session and an objective structured teaching exercise to help residents prepare for fellowship interviews.[11] However, no study to our knowledge has described the implementation of a comprehensive fellowship application series to help guide residents through each step of the fellowship application process (e.g., writing a personal statement, applying through ERAS, preparing for interviews, and postinterview communication).

We used the Kern model of curriculum development in medical education[12] to create the Fellowship Application Toolkit, which consists of educational workshops and resources to support IM residents applying to subspecialty fellowship. We evaluated the impact of this toolkit using pre- and postintervention surveys.

Methods

Setting and Participants

We developed the Fellowship Application Toolkit for residents at the University of California, San Francisco, IM residency program. We included all residents (categorical and primary care) as well as recent alumni who were applying to fellowship programs in the IM subspecialties. Workshops and resources were created and implemented over a 3-year period from 2018 through 2020.

Interventions/Curriculum Development

We used the Kern model of curriculum development in medical education[12] to create our toolkit. We first conducted a general needs assessment by reviewing the primary literature to identify existing fellowship application resources for residents. Next, we completed a targeted needs assessment by conducting two baseline focus groups of residents who had applied for subspecialty fellowship in the prior year (18 residents participated in total). Residents indicated dissatisfaction with the current level of support (“We get pretty much no guidance from the residency program right now,” “[I felt] completely on my own”) and a desire for more support from the residency program. They also provided input on curricular content, including the recommendation to create an overview fellowship advising document, an organized subspecialty-specific Q&A, an alumni database, and a repository of personal statements.

The goal of the Fellowship Application Toolkit was to support and guide residents though the fellowship application process. Our educational strategies were a mix of live workshops (in person or virtual), written resources, and one-to-one coaching and included the five components (Appendix A) listed below. We created a career advising Box folder on the residency website and stored all materials in this folder, including the PDF and PowerPoint presentations as well as video recordings of the virtual events for those who could not attend in real time. Implementation of the five components of the toolkit was as follows:

1. Fellowship application guidebook: We created a fellowship application guidebook with detailed logistics of the fellowship application process, including sections on resources, timeline, cost, short tracking, getting information on programs, letters of recommendation, personal statements, ERAS components, interviews, and postinterview communication (Appendix B). We solicited input from residents and fellowship program directors (PDs) during the development of the content. We also kept a running list of things to add based on resident feedback and updated the guidebook in January of each year to ensure it was up to date for each application cycle. For example, we added sample language about how to respond to certain postinterview communications based on resident request.
2. Fellowship Application Information Night: We hosted a 2-hour Fellowship Application Information Night as an in-person event in February 2019 and 2020 and as a virtual event in February 2021. The first hour was a general overview of the fellowship application process delivered via a PowerPoint presentation to the entire group (Appendix C), and the second hour included subspecialty-specific breakout rooms with the fellowship PD, associate PD, and/or fellows to discuss subspecialty-specific questions/experiences. Residents were asked to RSVP in advance and include which fellowship breakout room they planned to attend so that we could ensure that faculty from all fellowships of interest were present. Each room had at least one faculty member/fellow (range: one to five) and at least one resident (range: one to 15). Residents also submitted questions in advance. We shared this identical list of anonymous questions with the fellowship PDs and residents prior to the event and recommended that each room use this list of questions to start the conversation (Appendix D).
3. Alumni contact list: We emailed all alumni of our program who graduated in the prior 10 years and asked if they would be willing to be contacted by current residents to get fellowship and career advice. If so, we asked that they complete a Qualtrics form with their name, residency

Copyright © 2022 Huppert and Babik. This is an open-access publication distributed under the terms of the Creative Commons Attribution license.

graduation year, fellowship specialty and location, current position, clinical interests, and email address. We used this information to create an alumni contact list in an Excel document, organized by subspecialty. We contacted alumni annually to update the list as needed and published an updated list in January or February of each year.

4. Personal statement resources: We developed personal statement resources comprising a personal statement template (structured guidance by paragraph; see personal statement section of Appendix B) and a repository of example personal statements representing different specialties and career goals. To collect the example personal statements, we emailed recent fellowship applicants and graduates and asked if they would be willing to share their personal statements anonymously (they could remove names of mentors or identifying details per their preference). We emailed these resources to residents in March and also offered the opportunity to sign up to get a personal statement coach. Then, we paired all interested residents with a personal statement coach (in a different subspecialty from the one in which the resident was applying, to preserve confidentiality) to review and give input on their personal statement between April and May of each year. Each coach was a faculty member who was already involved in the fellowship application process in their own specialty. Therefore, we did not require formal training for the coaches but offered to meet with them if additional training was desired.
5. Virtual interview workshop and mock interviews: We held a 2-hour Interview Information Night in July of each year that covered interview preparation, tips for virtual interviewing, postinterview communication, and a panel Q&A with recent fellowship applicants (Appendix E). After this event, we emailed residents to ask whether they were interested in participating in a mock interview and paired all interested residents with a faculty member (in a different subspecialty from the one in which the resident was applying) for mock interviews. Mock interviews were structured as 30-minute interviews, with 20 minutes for mock interview questions (at least one behavioral question) and 10 minutes for feedback. All faculty were provided with sample questions and an evaluation rubric (Appendix F). Sample interview questions were gathered by soliciting common interview questions from prior applicants and adapting questions from other sources.[13]

Outcomes Measured/Curriculum Evaluation

To evaluate the efficacy of the Fellowship Application Toolkit, we designed a 39-item questionnaire (Appendix G) with Qualtrics Survey Software using Artino and colleagues' survey design process.[14] Item types included multiple-choice, 5-point Likert-scale, and open-ended questions. Survey validity was assessed using pilot testing. Residents were surveyed during the 2 weeks after Match Day both preintervention (2018) and postintervention (2019 and 2020) via electronic Qualtrics survey. Surveys were anonymous, and no incentives were provided. Both complete and incomplete responses were included in calculating the response rate.

Analysis of the Outcomes

Data were analyzed using Prism Software (GraphPad). We used descriptive statistics to summarize numeric responses. Comparisons between groups were made using the unpaired t test or two-sided Fisher exact test for small numbers. A p value of less than .05 was considered statistically significant. Qualitative survey data were analyzed using inductive content analysis to identify themes.[15] Laura A. Huppert and Jennifer M. Babik each coded the data, and differences were reconciled via iterative discussion.

IRB Statement

This work was deemed exempt by the University of California, San Francisco, Institutional Review Board.

Results

Survey response rates by year were as follows: 21 of 41 (51%) in 2018, 25 of 41 (61%) in 2019, and 24 of 43 (56%) in 2020. Demographic characteristics of the participants are shown in Table 1.

Feasibility

The five elements of the Fellowship Application Toolkit, including the approximate time and costs required for development and implementation, are shown in Appendix A. All events were developed and implemented by the two authors, with administrative support to help maintain the alumni contact list. Survey respondents reported their use of each resource: Thirty-six of 49 (74%) used the fellowship application guidebook, 37 of 49 (76%) attended the Fellowship Application Information Night, 15 of 49 (31%) used the alumni contact list, 10 of 24 (42%) used the personal statement resources, and 14 of 23 (61%) attended the virtual interview workshop (Figure 1A). Participants who did not use the resource were asked why—reasons varied by resource but most commonly were lack of knowledge of the resource or conflicting personal or residency obligations during

Copyright © 2022 Huppert and Babik. This is an open-access publication distributed under the terms of the Creative Commons Attribution license.

Table 1. Demographic Information About the Survey Respondents

Demographic	No. (%) of Survey Respondents 2018 ($n = 21$)[a]	2019 ($n = 25$)[a]	2020 ($n = 24$)[a]
Gender			
Female	7 (33)	10 (40)	12 (50)
Male	6 (29)	10 (40)	10 (42)
Nonbinary	0 (0)	0 (0)	0 (0)
Prefer not to answer	2 (10)	1 (4)	0 (0)
No response[b]	6 (29)	4 (16)	2 (8)
Current year in training			
Second-year resident (PGY 2)	3 (14)	3 (12)	2 (8)
Third-year resident (PGY 3)	11 (52)	9 (36)	15 (62)
One year postresidency	1 (5)	7 (28)	5 (21)
Two or more years postresidency	0 (0)	2 (8)	0 (0)
No response[b]	6 (29)	4 (16)	2 (8)
Race and ethnicity (select all that apply)			
American Indian or Alaskan Native	1 (5)	0 (0)	1 (4)
Asian	5 (24)	2 (8)	5 (21)
Black or African American	0 (0)	0 (0)	0 (0)
Hispanic, Latino, or Spanish origin	0 (0)	1 (4)	3 (12)
Native Hawaiian or Pacific Islander	0 (0)	1 (4)	1 (4)
White	9 (43)	16 (64)	15 (62)
Prefer not to answer	2 (10)	2 (8)	0 (0)
No response[b]	6 (29)	4 (16)	2 (8)
Underrepresented in medicine			
Underrepresented in medicine	1 (5)	2 (8)	5 (21)
Not underrepresented in medicine	12 (57)	17 (68)	17 (71)
Prefer not to answer	2 (10)	2 (8)	0 (0)
No response[b]	6 (29)	4 (16)	2 (8)
Fellowship program applied to			
Addiction medicine	1 (5)	0 (0)	2 (8)
Allergy/immunology	1 (5)	0 (0)	0 (0)
Cardiology	1 (5)	7 (28)	3 (12)
Clinical informatics[c]	N/A	N/A	1 (4)
Endocrinology	0 (0)	0 (0)	0 (0)
Gastroenterology	3 (14)	4 (16)	3 (12)
Geriatrics	0 (0)	1 (4)	0 (0)
Hematology/oncology	3 (14)	4 (16)	3 (12)
HIV primary care	0 (0)	0 (0)	0 (0)
Hospice and palliative medicine	1 (5)	1 (4)	0 (0)
Infectious diseases	0 (0)	2 (8)	4 (16)
Nephrology	0 (0)	0 (0)	0 (0)
Pulmonary/critical care medicine	4 (19)	2 (8)	4 (16)
Rheumatology	0 (0)	0 (0)	2 (8)
No response[b]	7 (33)	4 (16)	2 (8)

[a]There were no statistically significant differences between groups (one-way analysis of variance).
[b]Survey respondents who did not respond to the demographic data were labeled as "no response."
[c]The clinical informatics fellowship was only included in 2020.

the events (data not shown). Acceptability of each resource for the residents was measured explicitly via survey assessment, as described below. Fellowship PDs/associate PDs and personal statement coaches were surveyed and reported a high degree of satisfaction with the Fellowship Information Night and personal statement coaching, respectively (data not shown).

Perceived Efficacy of Each Resource

Of the respondents who used each resource, most found that they were extremely or very effective, including 30 of 36 (83%) who used the fellowship application guidebook, 31 of 37 (84%) who attended the Fellowship Application Information Night, 10 of 15 (67%) who used the alumni contact list, nine of 10 (90%) who used the personal statement resources, and 12 of 14 (86%) who attended the virtual interview workshop (Figure 1B). Free-text responses about the strengths and weaknesses of each resource were coded, and representative quotations are provided in Table 2.

Perceived Efficacy of the Residency Program's Fellowship Application Advising

We assessed resident perception of the efficacy of the residency program's fellowship application advising both pre- and postintervention. Of note, the years considered pre- and postintervention differ based on the year the relevant resource was introduced, as noted below.

The perceived efficacy of the residency program's fellowship application advising improved from pre- to postintervention in most aspects of fellowship application advising (Figure 2A), with the following number of respondents indicating extremely or very effective advising for each domain:

- Timeline of the fellowship application process: six of 18 (33%) in 2018 versus 32 of 41 (78%) in 2019/2020, $p = .002$.
- Writing a personal statement: six of 35 (17%) in 2018/2019 versus 14 of 21 (67%) in 2020, $p < .001$.
- Providing subspecialty-specific advice about the fellowship application process: three of 16 (19%) in 2018 versus 18 of 32 (56%) in 2019/2020, $p = .02$.
- Connecting with current or recent fellows: one of 14 (7%) in 2018 versus 19 of 35 (54%) in 2019/2020, $p = .02$.
- Postinterview communication: five of 31 (16%) in 2018/2019 versus nine of 19 (47%) in 2020, $p = .02$.
- Overall efficacy: four of 17 (24%) in 2018 versus 17 of 21 (81%) in 2020, $p < .001$.

Overall Experience Applying to Fellowship

We also evaluated the general experience applying to fellowship pre- and postintervention. Since resources were introduced in both 2019 and 2020, we considered 2020 as the postintervention time point. Between 2018 and 2020, there was a significant increase in the percentage of respondents who strongly or somewhat agreed that they felt supported by the residency program during the fellowship application process (seven of 15 [47%] in 2018 vs. 18 of 22 [82%] in 2020, $p = .04$) and those who indicated that they knew who they could turn to if they had questions about the fellowship advising process

Copyright © 2022 Huppert and Babik. This is an open-access publication distributed under the terms of the Creative Commons Attribution license.

MedEdPORTAL® The AAMC Journal of Teaching and Learning Resources

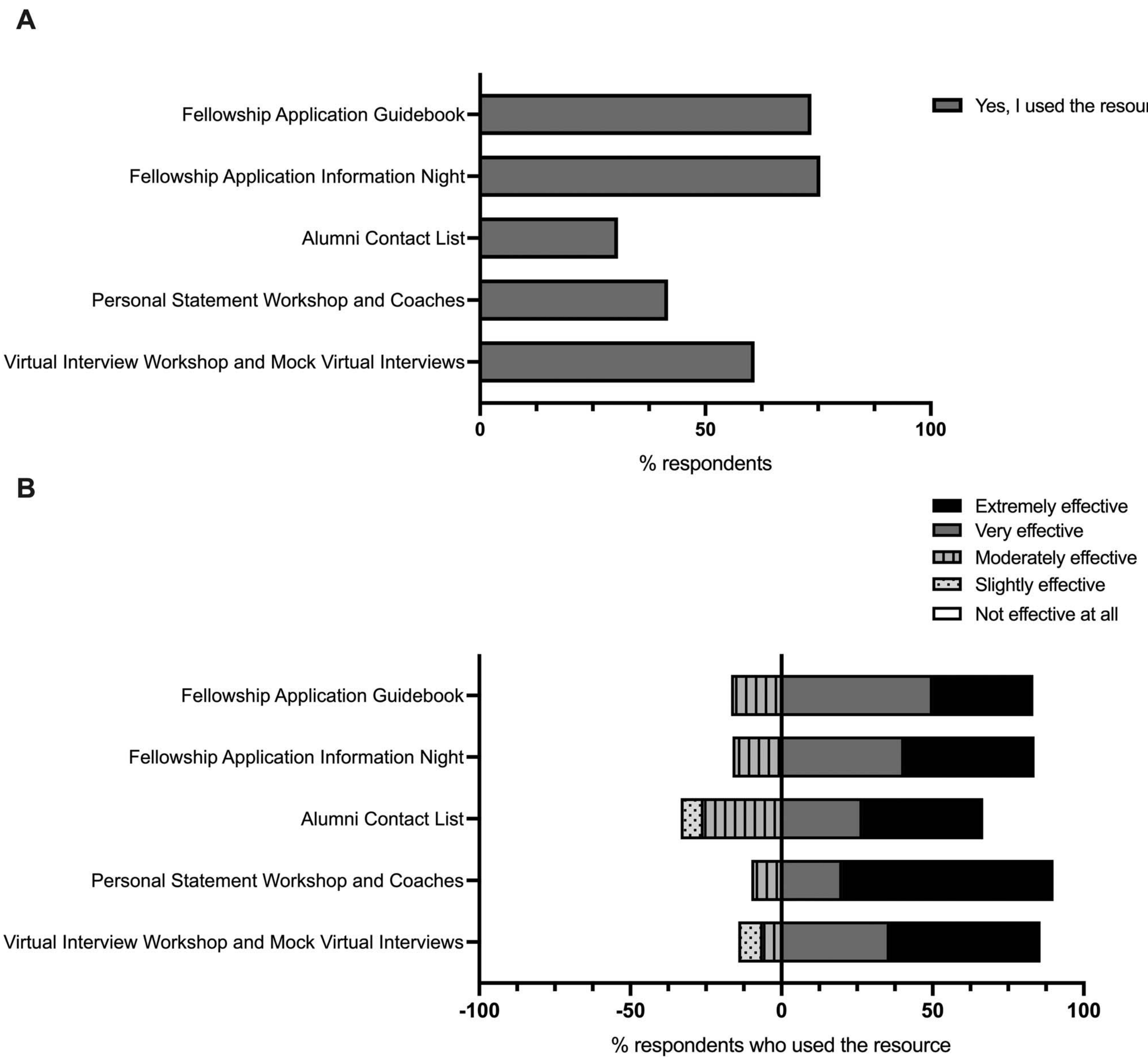

Figure 1. Use and efficacy of the fellowship application advising resources. A: The percentage of survey respondents who used each fellowship application resource. B: The respondents who used each resource indicated the perceived efficacy of each resource. Responses to the right of the vertical baseline (0% axis) show the percentage of respondents who answered *extremely effective* or *very effective*. Responses to the left of the vertical baseline show the percentage of respondents who answered *moderately effective, slightly effective,* or *not effective at all.*

(six of 15 [40%] in 2018 vs. 20 of 22 [91%] in 2020, $p = .002$). There was no change in the number of respondents who agreed that applying to fellowship was stressful (13 of 15 [87%] in 2018 vs. 18 of 22 [82%] in 2020, $p > 0.99$; Figure 2B).

Discussion

We describe the development, implementation, and evaluation of the five-component Fellowship Application Toolkit for IM residents. Despite the importance of the fellowship application process to career trajectory, to our knowledge this is the first published description of a curriculum to guide residents through this critical phase in their training, as well as being one that can be adapted for use at other programs.

The resources in our toolkit were widely used and deemed to be efficacious. Specifically, over 60% of respondents used the fellowship application guidebook, attended the Fellowship Application Information Night, and participated in the virtual

Copyright © 2022 Huppert and Babik. This is an open-access publication distributed under the terms of the Creative Commons Attribution license.

Table 2. Overall Strengths and Weaknesses of the Fellowship Application Advising Resources: Results From Content Analysis of Responses to Open-Ended Items

Fellowship Application Advising Resource	Strengths[a]	Weaknesses[a]
1. Fellowship application guidebook	"Provided a good timeline leading up to and the materials needed for the application."<br>"Really walked me through the application process in a way that was easy to understand and alleviated anxiety."<br>"Practical tips, advice from prior applicants, up to date."	"More comments from prior applicants would be helpful."<br>"One place where I could have used more guidance was on discussing rank lists."<br>"More fellowship-specific advice for very competitive subspecialties."
2. Fellowship Application Information Night	"Having actual fellowship program directors present to answer questions and dispel myths was particularly effective."<br>"I like being able to submit anonymous questions to the fellowship leadership in advance."	"It would be great if we could make this more personalized especially in specialty breakouts. Possibility for 1:1 time?"<br>"Email materials beforehand so we can review and think about questions to bring to the session."<br>"Would be great to have more than one hour with the fellowship [program directors]."<br>"Ensure clinical coverage so everyone can attend."
3. Alumni contact list	"Helped me talk to people at each of the programs I was interviewing at to obtain more information in advance."	"Continue to add more alumni! Helpful to have folks not in academia for those who are interested in non-academic paths."
4. Personal statement resources and coaches	"Rapidity of response from the coach, follow up by the coach, ideas to better articulate ideas and goals."<br>"It was so nice to have personalized advice and also have a set timeline to make me write my personal statement in advance to have enough time for edits."	"I didn't agree with the edits my coach provided."
5. Virtual interview workshop and mock virtual interviews	"All of the details about lighting, framing, etc. were helpful. On the interview trail, most people didn't get this from their programs and I feel like it gave us an advantage."<br>"I really appreciated the example questions to prepare. I referred to the list of questions a lot prior to my first interview to work out my answers, and found that particularly helpful. I also referred to the section on how to handle post-match communication quite a bit."<br>"I was frequently complimented on my set up during interviews which I attribute to tips from this session. It was also nice to run through the 'standard' questions so that I really had to prepare in advance, which helped me be less anxious during my actual interviews."	"A lot of this is really prep on our own, and doing a single interview with the faculty person may not be a great representation to them of our preparedness or what we need to focus on."<br>"Written feedback from interviewer could be helpful."

[a]Shown are representative quotations from respondents responding to the open-ended item "What were the overall strengths and weaknesses/areas for improvement of the given fellowship application advising resource?"

interview workshop. Four of the five resources were also rated as extremely or very effective by over 80% of survey respondents. Of the five components of the toolkit, the alumni contact list was utilized by the fewest number of respondents and also rated as the least effective. The main reason residents reported that they did not use it was because they already had contacts at other institutions. Interestingly, more respondents used the alumni contact list and felt it was effective during the 2020 virtual interview season than during the 2019 in-person application season (data not shown), so it may be particularly important if residents do not get to meet fellows in person on the interview trail. The free-text responses provided additional context about the use and efficacy of these events. For example, for the Fellowship Application Information Night, free-text responses highlighted specific strengths, such as the ability to submit anonymous questions, as well as specific recommendations for improvement, such as the desire to spend more time with fellowship PDs and a request to incorporate one-to-one time with fellowship PDs.

Not only were the individual resources deemed effective but, even more importantly, residents indicated that the overall efficacy of the residency program's fellowship advising improved after the introduction of these resources. Residents also felt more supported by the residency program and knew who they could turn to with questions postintervention. Prior studies indicated that residents desired more formal advising about the fellowship application process in their residency curriculum,[4] so our data demonstrate that these resources can achieve this goal.

Even though residents felt supported, most still felt that the fellowship application process was stressful both pre- and postintervention. Prior studies also found that IM residents reported stress associated with career planning[8]

Copyright © 2022 Huppert and Babik. This is an open-access publication distributed under the terms of the Creative Commons Attribution license.

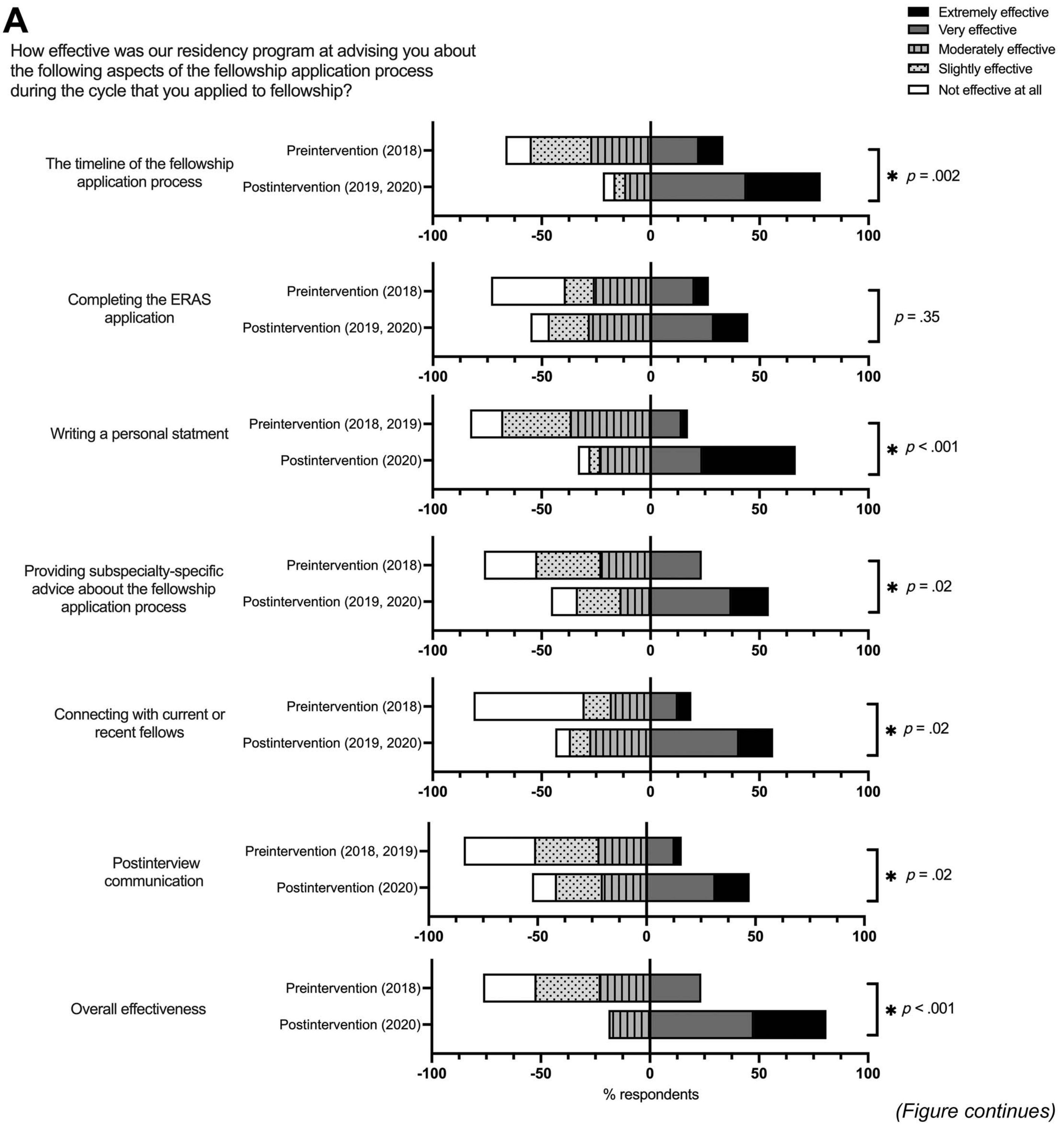

(Figure continues)

Copyright © 2022 Huppert and Babik. This is an open-access publication distributed under the terms of the Creative Commons Attribution license.

Figure 2 *(continued)*

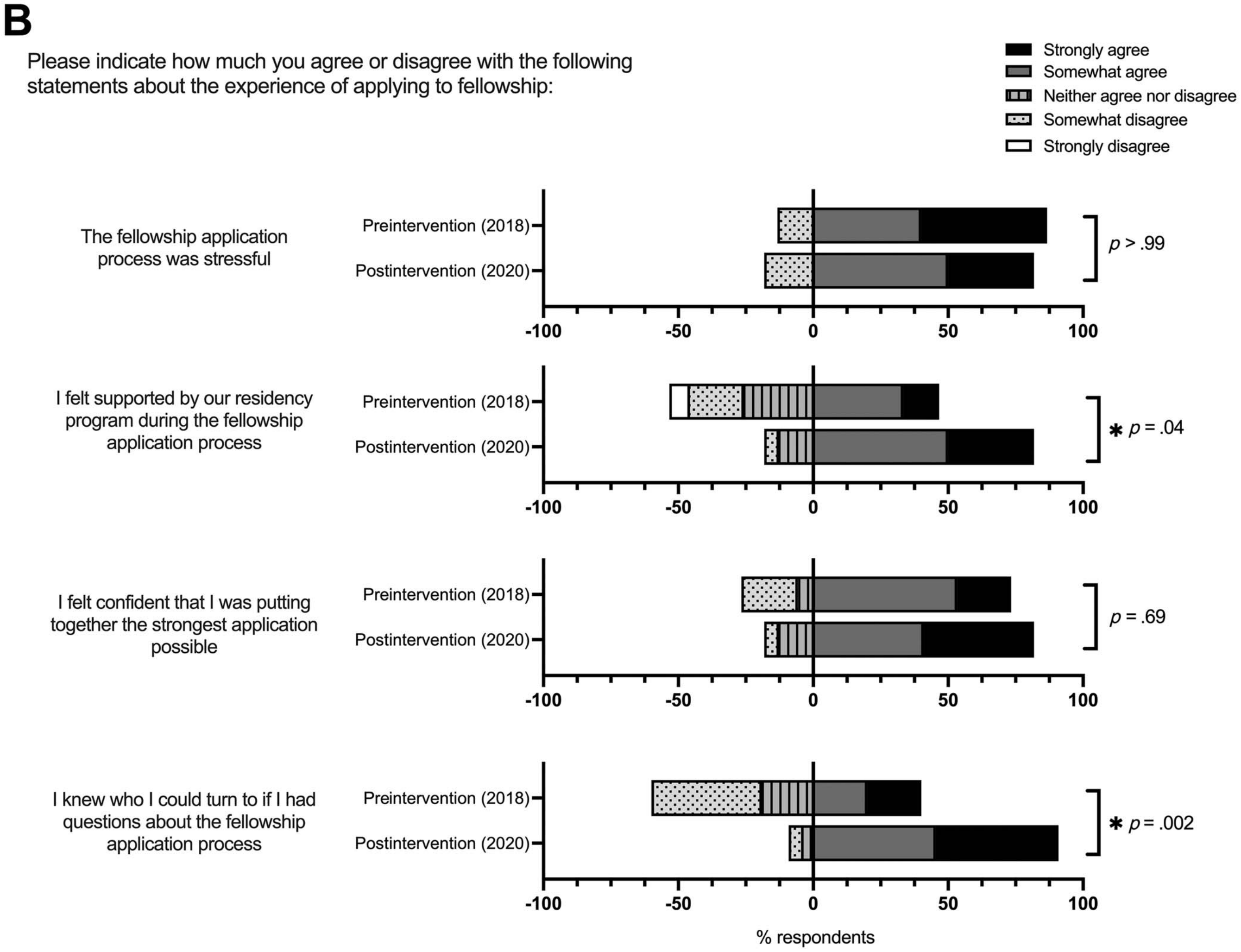

Figure 2. Efficacy of resident program advising and overall experience applying to fellowship pre- versus postintervention. A: Survey respondents were asked how effective the residency program was at providing advising about various aspects of the fellowship advising process. Responses to the right of the vertical baseline (0% axis) show the percentage of respondents who answered *extremely effective* or *very effective.* Responses to the left of the vertical baseline show the percentage of respondents who answered *moderately effective, slightly effective,* or *not effective at all. Extremely effective* and *very effective* were considered favorable responses, and the preintervention versus postintervention favorable responses were compared; statistical significance is indicated with an asterisk (*), and *p* values are shown for each item. Of note, preintervention and postintervention years differ depending on the time that the relevant resource was introduced, as indicated. B: Survey respondents were asked about their overall experience applying to fellowships. Responses to the right of the vertical baseline (0% axis) show the percentage of respondents answering that they *strongly agreed* or *somewhat agreed* with the statement shown. Responses to the left of the vertical baseline show the percentage of respondents who answered that they *neither agreed nor disagreed, somewhat disagreed,* or *strongly disagreed* with the statement shown. *Strongly agree* and *somewhat agree* were considered favorable responses, and the preintervention (2018) versus postintervention (2020) favorable responses were compared; statistical significance is indicated with an asterisk (*), and *p* values are shown for each item.

and subspecialty career choice.[16] Further research should evaluate why applying to fellowship is stressful and what countermeasures, if any, can be taken to mitigate the stress associated with this process.

In terms of feasibility, three of the five resources were introduced in 2019, and two additional resources were added in 2020, so it is possible to add events sequentially rather than trying to introduce them all in a single year. Some events were held in person and others virtually (especially in 2020 due to the COVID-19 pandemic), so both formats are possible. We found that the virtual format was ultimately easier to organize (we did not have to secure rooms, food, etc.) and also easier for both residents and faculty to attend after hours from home (or work).

Copyright © 2022 Huppert and Babik. This is an open-access publication distributed under the terms of the Creative Commons Attribution license.

Costs were minimal, but there were notable investments of time required to create these resources. We are hopeful that by sharing the details of our curriculum development and evaluation, it will be easier to adapt and implement similar resources at other residency programs.

There are several limitations to this work. First, the survey response rate was between 51% and 61% per year and may have been biased towards residents who had a positive view of the resources. Second, we developed these resources for IM residents at a large university medical center, so considerations may be different in smaller programs without associated fellowship programs to provide direct support. Third, almost all residents at our program are US medical graduates who apply to (mostly) university fellowship programs; our resources and guidance were thus aimed at this specific population and may not be generalizable to all residency programs. Therefore, programs might consider a local needs assessment based on the experiences of their recent fellowship applicants to determine unique stressors or challenges that should be addressed. In the future, it will be important to determine best practices for fellowship application advising across all types of residency programs.

Conclusions

We developed the five-component Fellowship Application Toolkit for IM residents applying to subspecialty fellowships. This toolkit was highly utilized, was very effective, and led to improvements in the residents' perceived level of support from the residency program. The toolkit can be adapted for use at other residency programs to help strengthen the advising for residents applying to subspecialty fellowships.

Appendices

A. Elements of the Fellowship Application Toolkit.docx

B. Fellowship Application Guide.docx

C. Fellowship Application Information Night.pptx

D. Subspecialty Breakout Room Questions.docx

E. Preparing for Virtual Interviews.pptx

F. Mock Virtual Interview.docx

G. Survey Instrument.docx

All appendices are peer reviewed as integral parts of the Original Publication.

Laura A. Huppert, MD: Fellow, Hematology/Oncology Division, Department of Medicine, University of California, San Francisco, School of Medicine; ORCID: https://orcid.org/0000-0002-8512-5608

Jennifer M. Babik, MD, PhD: Associate Professor, Infectious Disease Division, Department of Medicine, Associate Program Director, Internal Medicine Residency, and Associate Program Director, Infectious Disease Fellowship, University of California, San Francisco, School of Medicine

Acknowledgments

We thank Rebecca Berman, MD, and Nancy Li for administrative assistance in delivering this curriculum to the internal medicine residents.

Disclosures

None to report.

Funding/Support

None to report.

Prior Presentations

Huppert LA, Babik JM. Development of a toolbox for internal medicine residents applying for subspecialty residents applying for subspecialty fellowships. Presented virtually at: APDIM Spring Virtual Meeting, AAIM Online; April 12, 2021.

Raj J, Lai C, Babik JM, Huppert LA, Schaeffer S. Best practices for virtual interviewing for the 2021-22 internal medicine residency application process: promoting equity and opportunities. Presented virtually at: Alliance for Academic Internal Medicine Webinar; September 9, 2021.

Ethical Approval

The University of California, San Francisco, Institutional Review Board deemed further review of this project not necessary.

References

1. West CP, Dupras DM. General medicine vs subspecialty career plans among internal medicine residents. *JAMA*. 2012;308(21):2241-2247. https://doi.org/10.1001/jama.2012.47535
2. ERAS statistics. Association of American Medical Colleges. Accessed January 11, 2021. https://www.aamc.org/data-reports/interactive-data/eras-statistics-data
3. Huppert LA, Santhosh L, Babik JM. Trends in US internal medicine residency and fellowship applications during the COVID-19 pandemic vs previous years. *JAMA Netw Open*. 2021;4(4):e218199. https://doi.org/10.1001/jamanetworkopen.2021.8199
4. Bosslet GT, Burkart KM, Miles MC, Lenz PH, Huebert CA, McCallister JW. Preparing for fellowship in internal medicine: steps for success with a focus on pulmonary and/or critical care programs. *Ann Am Thorac Soc*. 2015;12(4):567-573. https://doi.org/10.1513/AnnalsATS.201501-033AS

Copyright © 2022 Huppert and Babik. This is an open-access publication distributed under the terms of the Creative Commons Attribution license.

5. Hamnvik OPR. Applying for fellowship: what you need to know. NEJM Resident 360. July 21, 2017. Accessed May 4, 2021. https://resident360.nejm.org/expert-consult/applying-for-fellowship-what-you-need-to-know-2
6. Pomerantz RM. Applying for a sub-specialty fellowship: some tips and advice from a former program director. *J Community Hosp Intern Med Perspect*. 2011;1(3):8087. https://doi.org/10.3402/jchimp.v1i3.8087
7. Joshi AA. Tips and tricks for fellowship interviews. American College of Cardiology. October 21, 2020. Accessed November 10, 2021. https://www.acc.org/Membership/Sections-and-Councils/Fellows-in-Training-Section/Section-Updates/2020/10/20/20/42/Tips-and-Tricks-For-Fellowship-Interviews
8. Garcia RL, Windish DM, Rosenbaum JR. Resident career planning needs in internal medicine: a qualitative assessment. *J Grad Med Educ*. 2010;2(4):518-522. https://doi.org/10.4300/JGME-D-10-00086.1
9. Englander R, Carraccio C, Zalneraitis E, Sarkin R, Morgenstern B. Guiding medical students through the Match: perspectives from recent graduates. *Pediatrics*. 2003;112(3):502-505. https://doi.org/10.1542/peds.112.3.502
10. Bradner M, Crossman SH, Vanderbilt AA, Gary J, Munson P. Career advising in family medicine: a theoretical framework for structuring the medical student/faculty advisor interview. *Med Educ Online*. 2013;18(1):21173. https://doi.org/10.3402/meo.v18i0.21173
11. Sinclair M, Raimo J, Wong K, Kline M, Friedman K. A novel curriculum to prepare internal medicine residents for fellowship interviews. *J Med Educ Curric Dev*. 2019;6:2382120519855939. https://doi.org/10.1177/2382120519855939
12. Kern DE, Thomas PA, Hughes MT, eds. *Curriculum Development for Medical Education: A Six-Step Approach.* 2nd ed. Johns Hopkins University Press; 2009.
13. Interviewing for residency: frequently asked interview questions. Association of American Medical Colleges. Accessed January 26, 2022. https://students-residents.aamc.org/media/11096/download
14. Artino AR Jr, La Rochelle JS, Dezee KJ, Gehlbach H. Developing questionnaires for educational research: AMEE Guide no. 87. *Med Teach*. 2014;36(6):463-474. https://doi.org/10.3109/0142159X.2014.889814
15. Hsieh HF, Shannon SE. Three approaches to qualitative content analysis. *Qual Health Res*. 2005;15(9):1277-1288. https://doi.org/10.1177/1049732305276687
16. Smith LG, Feit E, Muller D. Internal medicine residents' assessment of the subspecialty fellowship application process. *Acad Med*. 1997;72(2):152-154. https://doi.org/10.1097/00001888-199702000-00026

Received: August 13, 2021
Accepted: December 29, 2021
Published: March 14, 2022

Copyright © 2022 Huppert and Babik. This is an open-access publication distributed under the terms of the Creative Commons Attribution license.
